# Genome assembly, Full-length transcriptome, and isoform diversity of Red Snapper, *Lutjanus argentimaculatus*

**DOI:** 10.1038/s41597-024-03633-1

**Published:** 2024-07-18

**Authors:** Mudagandur S. Shekhar, Vinaya Kumar Katneni, Ashok Kumar Jangam, Karthic Krishnan, Sudheesh K. Prabhudas, Roja Jayaraman, Jesudhas Raymond Jani Angel, Muniyandi Kailasam

**Affiliations:** 1 Centre for Bioinformatics, ICAR-Central Institute of Brackishwater Aquaculture, No 75, Santhome High Road, MRC Nagar, Chennai, 600028 Tamil Nadu India; 2 Crustacean Culture Division, ICAR-Central Institute of Brackishwater Aquaculture, No 75, Santhome High Road, MRC Nagar, Chennai, 600028 Tamil Nadu India; 3 Finfish Culture Division, ICAR-Central Institute of Brackishwater Aquaculture, No 75, Santhome High Road, MRC Nagar, Chennai, 600028 Tamil Nadu India

**Keywords:** Functional genomics, Genomics, Genome informatics

## Abstract

The mangrove red snapper, *Lutjanus argentimaculatus*, is a marine food fish of economic and aquaculture importance. The application of genomic selection-based breeding programs for this species is limited by the absence of a reference genome and transcriptome profiles. The current study attempted to fill this void by generating genomic and transcriptomic resources for red snapper. Using PacBio long reads, and Arima Hi-C linked reads, a scaffold-level genome assembly was generated for *L. argentimaculatus*. The assembly is of 1.03 Gb comprising of 400 scaffolds with N50 of 33.8 Mb and was assessed to be 97.2% complete upon benchmarking with BUSCO. Full-length transcriptome generated with PacBio Iso-Sequencing strategy using six tissues (muscle, gills, liver, kidney, stomach, and gonad) contained 56,515 isoforms belonging to 18,108 unique genes with N50 length of 3,973 bp. The resources generated will have potential applications in the functional studies, conservation, broodstock management and selective breeding programmes of *L. argentimaculatus*.

## Background & Summary

**Fig. 1:**
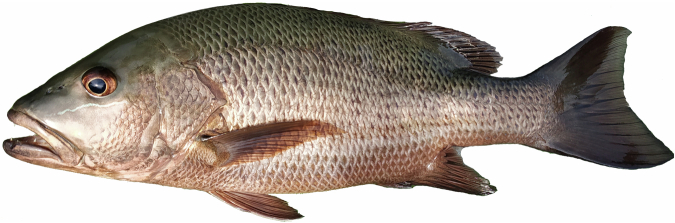
Image of Red Snapper collected from ICAR-Central Institute of Brackishwater aquaculture experimental station, for whole genome and transcriptome sequencing project.

*Lutjanus argentimaculatus* (Forsskål, 1775), also commonly known as mangrove jack or mangrove red snapper belongs to species of marine ray-finned fish, of the family Lutjanidae (Fig. 1). It is subdivided into four subfamilies and 17 genera with around 110 species. It is an important high value commercial food fish as it is a fast-growing species, with high consumer demand. This fish is a potential candidate species for aquaculture as it is a euryhaline, able to grow in fresh, brackish, and marine habitats and can be reared easily in captive conditions. Its habitat has a wide tropical and sub-tropical Indo-Pacific range including Greece^1^ Middle East, Southeast Asia, and Southern China. The assessment of the population genetic structure of *L. argentimaculatus* from Indian waters using mitochondrial regions^2^ and microsatellite markers^3^ indicate probable panmixia. *L. argentimaculatus* is considered as a potential candidate species for aquaculture in India, due to the recent advancement made in seed production technology of this fish^4^. For sustainable seed production and aquaculture practice, a prior knowledge of the genetic resources of fishes is vital. Deciphering the genome and transcriptome of fish plays an important role in captive breeding for stock enhancement, brood stock management and selective breeding.

Currently, very few attempts have been made to generate draft genomes for lutjanid fish. Very little genomic and transcriptomic information is available for any species belonging to the Lutjanidae family. Based on short read sequencing technology, the first draft genome of a closest species was assembled for *Lutjanus campechanus*, representing 770 Mb, and 69% of the genome in 67,254 scaffolds (N50 = 16,803 bp)^5^. The other assembly for *Lutjaus campechanus* was found to be more fragmented with 140,690 scaffolds (N50 = 20,750 bp)^6^. Later Lai *et al*.^7^ assembled the crimson snapper (*Lutjanus erythropterus*) using Single-Tube Long Fragment Read sequencing technology and Hi-C data representing 973 Mb and 12,709 scaffolds (N50 = 40.64 Mb). In the present study, PacBio Sequel II long read sequencing data was generated to assemble the genome and Iso-Seq data was used to assist with gene annotation. This resulted in a superior genome assembly quality with high N50 values both at contig and scaffold levels. The Iso-Seq based full length transcriptome revealed large isoform diversity presence in *L. argentimaculatus*.

## Methods

### Specimen of *Lutjanus argentimaculatus*

**Fig. 2:**
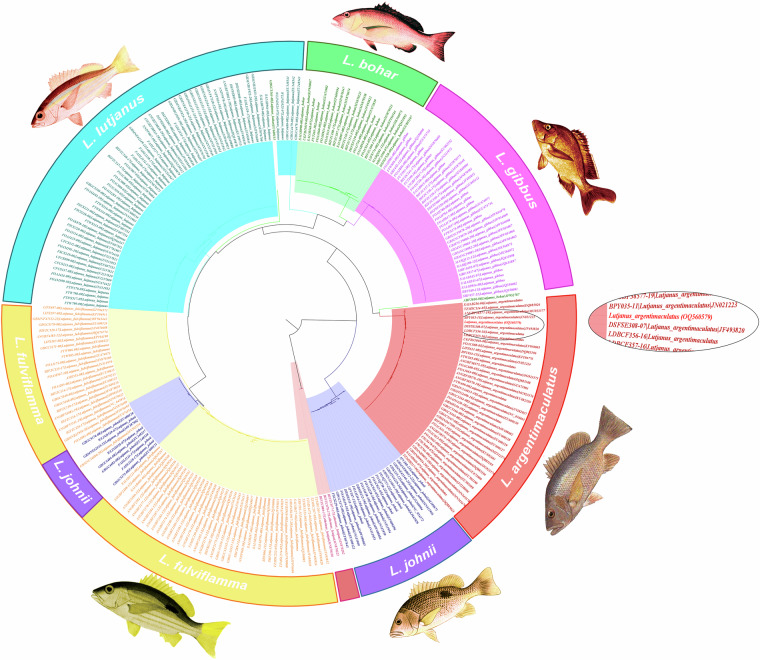
Maximum likelihood phylogenetic tree based on COI gene sequence of *Lutjanus argentimaculatus* and other Lutjanus species and *Lethrinus lentjan* as an outgroup. Fish images were obtained from FAO species catalogue and modified^65^.

A specimen of the *L. argentimaculatus* being maintained at Muthukadu Experimental Station of ICAR – CIBA (Chennai, India) was used for generating the necessary sequence data for building genome assembly. The species identity of the mangrove red snapper was confirmed based on the partial sequence of the barcode gene, Cytochrome C Oxidase I (COI) using primers, F2 - 5′TCGACTAATCACAAAGACATCGGCAC3′ and R1 - 5′TAGACTTCTGGGTGGCCAAAGAATCA3′^8^. The partial sequence of COI gene was obtained by Sanger sequencing method and submitted to GenBank under the accession number, OQ560579. The phylogenetic analysis to assess the lineage of *L. argentimaculatus* sample used in the current study was performed using the COI sequence. Multiple accessions of COI sequence were obtained from the Barcode of life data systems, (bold database) for different species of *Lutjanus*, and *Lethrinus lentjan* was used as an out group. Supplementary Table 1 summarizes the number of accessions used for generating the phylogenetic tree. MEGA X software^9^ was used to generate the Maximum Likelihood tree with a 1000 bootstrap iterations implementing the Hasegawa-Kishino-Yano model with Gamma and Invariant site distribution rates (HKY + G + I). The final tree was visualized using FigTree v1.4.32^10^. The phylogenetic analysis for the COI sequence (OQ560579) of *L. argentimaculatus* sample used for genome sequencing revealed clustering with other accessions of *L. argentimaculatus* (Fig. 2).

### Genomic data generation

The genomic DNA was extracted from muscle tissue of *L. argentimaculatus* using Genomic-tip 100/G kit (Qiagen, Hilden, Germany). DNA quantification carried out using Qubit 3.0 fluorometer (Thermofisher Scientific, Massachusetts, USA) using DNA HS assay kit (Thermofisher Scientific, Massachusetts, USA). DNA purity was checked using NanoDrop 2000 (Thermofisher Scientific, Massachusetts, USA). The integrity of DNA was evaluated on 1% agarose gel. Approximately 48 µg of total DNA with a concentration of 322 ng/µl was obtained.

PacBio long read generation was performed using the high molecular weight genomic DNA. The genomic DNA was sheared using Megaruptor (Diagenode Inc, NJ, USA) for library preparation. The gel electropherogram of Femto pulse run (Agilent Technologies, California, USA) showed average size of 42,046 bp and 31,506 bp of sheared DNA and size selected DNA respectively. The library preparation was performed with SMRTbell® Express Template Prep Kit 2.0 (Pacific Biosciences, California, USA) and size selection was carried out using BluePippinTM (Sage Science, Massachusetts, USA). The library was sequenced on PacBio Sequel II platform (Pacific Biosciences, California, USA) and the raw data processing was carried out using SMRTlink v10.1.0.119588 software. (Pacific Biosciences, California, USA). A total of 134.6 Gb PacBio Sequel II Continuous Long Read (CLR) sequence data was generated with 8,515,294 number of sequences, 117.2 Gb unique molecular yield, maximum length of 180,279 bp and a mean subread length per ZMW of 15,808 bp (Supplementary Table S2).

The Illumina genomic DNA sequencing library was constructed using KAPA HyperPlus kit (Basel, Switzerland) as per manufacturers’ protocol. The library size was assessed using TapeStation (Agilent Technologies, California, USA). The libraries with average insert size 316 bp analysed through tapestation were later sequenced in Novaseq 6000 (Illumina) platform. About 461 million reads/ 69.70 Gb data was generated. The Illumina paired-end reads were used to assess the genome length.

For Hi-C data generation, tissue crosslinking and proximity ligation was followed by library preparation using the Proximo Hi-C Kit (Animal) (Phase genomics, Washington, USA). The library was sequenced on Illumina NovaSeq 6000 platform in 150 bp paired-end mode to generate 449 million paired reads (67.44 Gb) of which 93.19% bases have Q30 quality score and 16.88% inter-contig high quality read pairs Supplementary Table S3. The Hi-C reads were used in scaffolding of assembly contigs.

### Transcriptome data generation

Six tissues (muscle, gills, liver, kidney, stomach, and gonad) collected from adult *L. argentimaculatus* fish were flash frozen in liquid nitrogen and stored at −80 °C. The total RNA was extracted using TRIzol (DSS Takara, CA, USA) method and purified using Nucleospin RNA cleanup kit (Machery Nagel, Germany). RNA quantification was carried out using Qubit 3.0 fluorometer using RNA HS kit (Thermofisher Scientific, Massachusetts, USA). RNA integrity was checked using Bioanalyzer 2100 (Agilent Technologies, California, USA) and the quantity was measured using Nanodrop 2000 (Thermofisher Scientific, Massachusetts, USA). The total RNA was enriched for mRNA using NEBNext® Poly(A) mRNA Magnetic isolation module (NEB Inc., France) and cDNA was prepared using NEBNext® Single Cell/Low Input cDNA Synthesis and Amplification Module (New England Biolabs, Hitchin, UK). The cDNA was purified using pronex beads (Promega, Madison, USA).

To generate Illumina compatible RNAseq libraries, the cDNA was used according to the manufacturer’s instruction as per NEBNext II RNA Library Prep Kit for Illumina® (NEB Inc., France). The libraries were quantified with qubit 3.0 fluorometer (Thermo Fisher Scientific, USA) using DNA HS assay kit (Thermo Fisher Scientific, USA). The libraries were subjected to fragment analysis using Femto Pulse pulsed field capillary electrophoresis system using Ultra Sensitivity NGS kit, and High Sensitivity D1000 ScreenTape (Agilent Technologies, California, USA) following manufacturer’s protocol to evaluate the insert sizes. The insert sizes obtained for the libraries were ranging from 295 bp to 362 bp. Sequencing data was obtained from the qualified libraries using Illumina NovaSeq 6000, S4 Flow Cell (2x150bp read lengths). Sequencing data (total reads and total bases) was obtained from gills (18.47 Gb), kidney (18.23 Gb), liver (17.41 Gb), muscles (17.07 Gb), stomach (23.07 Gb), and from gonad (14.54 Gb) is shown in (Supplementary Table 4). The RNAseq data were quality trimmed using trimmomatic v0.39^11^

To generate the Long-read Iso-Seq data, the cDNA from individual tissues were pooled in equimolar ratio and then subjected to Library preparation. The library preparation from the pooled cDNA was performed with SMRTbell® Express Template Prep Kit 2.0 (Pacific Biosciences, California, USA). Library size was checked using bioanalyzer 2100 (Agilent Technologies, California, USA) and the observed average insert size was 4,564 bp. Sequence data was generated using Pacific Biosciences (PacBio) long-read RNA sequencing (Iso-Seq). A total of 42.64 million subreads were generated by Iso-Seq with subreads summing upto 138.56 Gb. (Supplementary Table 5).

### Genome size estimation

**Fig. 3:**
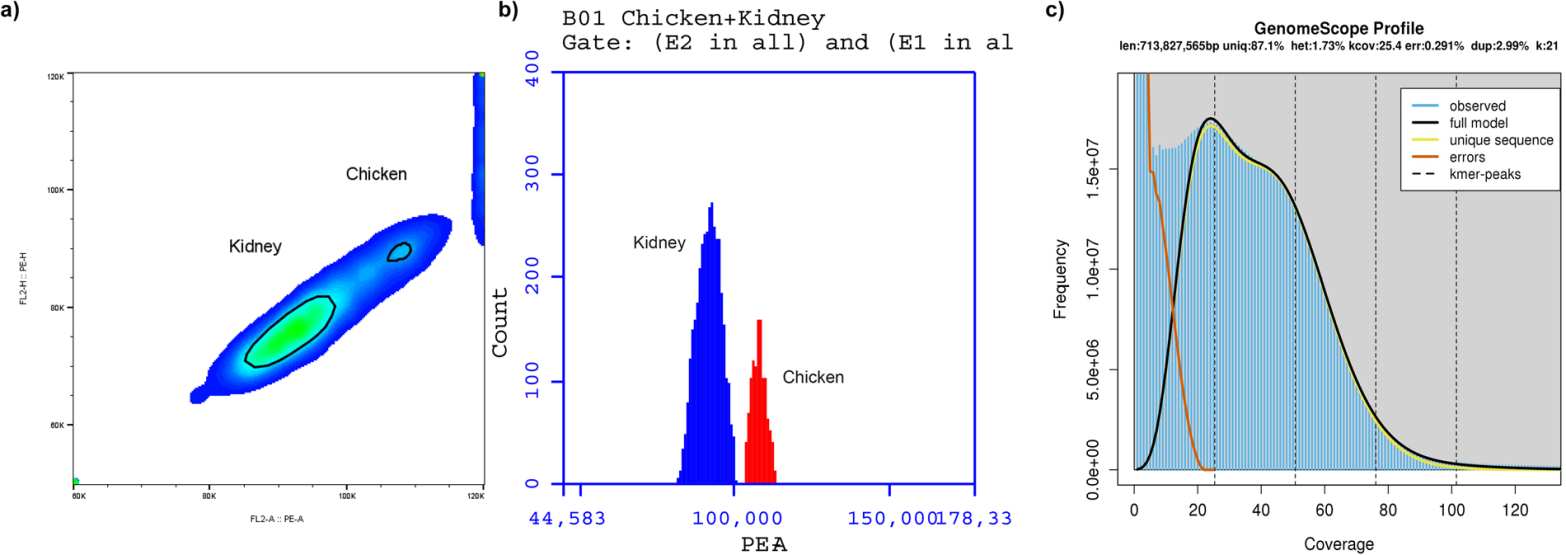
Genome estimation profiles of *L. argentimaculatus*. (**a**) Density plot and histogram from kidney tissue of *L. argentimaculatus* and chicken erythrocytes (control). (**b**) Histogram depicting the count of events for Kidney tissue of *L. argentimaculatus* and chicken erythrocytes. (**c**) Genome length assessment of *L. argentimaculatus* by K-mer frequency generated using Jellyfish and Genomescope.

Kidney tissue sample of *L. argentimaculatus* was homogenized with 750 µl of LB01 lysis buffer (15 mM Tris, 2 mM disodium-EDTA, 0.5 mM spermine tetrahydrochloride, 80 mM KCl, 20 mM NaCl and 0.1% v/v TritonX-100). The homogenized sample was filtered through cell strainer (40 µm) (Corning Inc., New York, USA). The filtrate was treated with 1 µl of RNase A (Qiagen, Hilden, Germany) and propidium iodide (12 µl) (ThermoFisher Scientific, Massachusetts, USA). The control sample, chicken erythrocytes (20 µl) (BD^TM^ DNA QC Particles kit, BD Biosciences, California, USA) was mixed with LB01 buffer (480 µl), RNase A (1 µl) and propidium iodide (12 µl). The test and control samples were incubated in dark at 4 °C for 30 min before flow cytometry analysis. The flow cytometry readings were acquired on BD Accuri^TM^ C6 flow cytometer for 10,000 events, flow rate (14 µl/min) and 10 µm core size setting. Gating of the density plots was carried out and histogram data was acquired for genome size estimation using FlowJo™ Software (BD Life Sciences California, USA). The density plot and histogram obtained from flow cytometer for genome size estimation from kidney tissue is shown in Fig. 3a,b, and genome size of *L. argentimaculatus* was found to be 1.06 pg (1.037 Gb). The genome size estimation for Lutjanidae family ranges from 0.78 Gb to 1.37 Gb using different methods (https://www.genomesize.com/search.php). In our previous study we have reported the genome size for *L. argentimaculatus* as 0.93 Gb, using blood cells by flow cytometry analysis^12^. In the present study we have used the kidney cells of the fish which revealed 1.037 Gb genome size estimate. This slight variation in the estimate may have been impacted by the use of different tissue type for the target species. However, our estimates of *L. argentimaculatus* genome size is typically close and within the range values for other lutjanids available in the animal genome size database.

Apart from the genome size estimates from flow cytometry, insights about the genome haploid length and repeat length is revealed by counting the K-mers from Illumina paired end reads. The K-mer frequency for *L. argentimaculatus* genome was estimated from the Illumina paired end reads using Jellyfish 2.2.3^13^ to count the canonical 21 K-mers with the hash size as 20 G. The counts histogram was later given as input to the online tool Genomescope^14^ to get the K-mer frequency peaks. The K-mer frequency for *L. argentimaculatus* revealed the genome haploid length to be 713 Mb and genome repeat length as 91 Mb with genome unique length of 621 Mb (Fig. 3c). The genome size estimated by K-mer analysis for other *Lutjanus* species is around 1.117 Gb (*Lutjanus campechanus)*^5^.

### Genome assembly of *Lutjanus argentimaculatus*

**Fig. 4:**
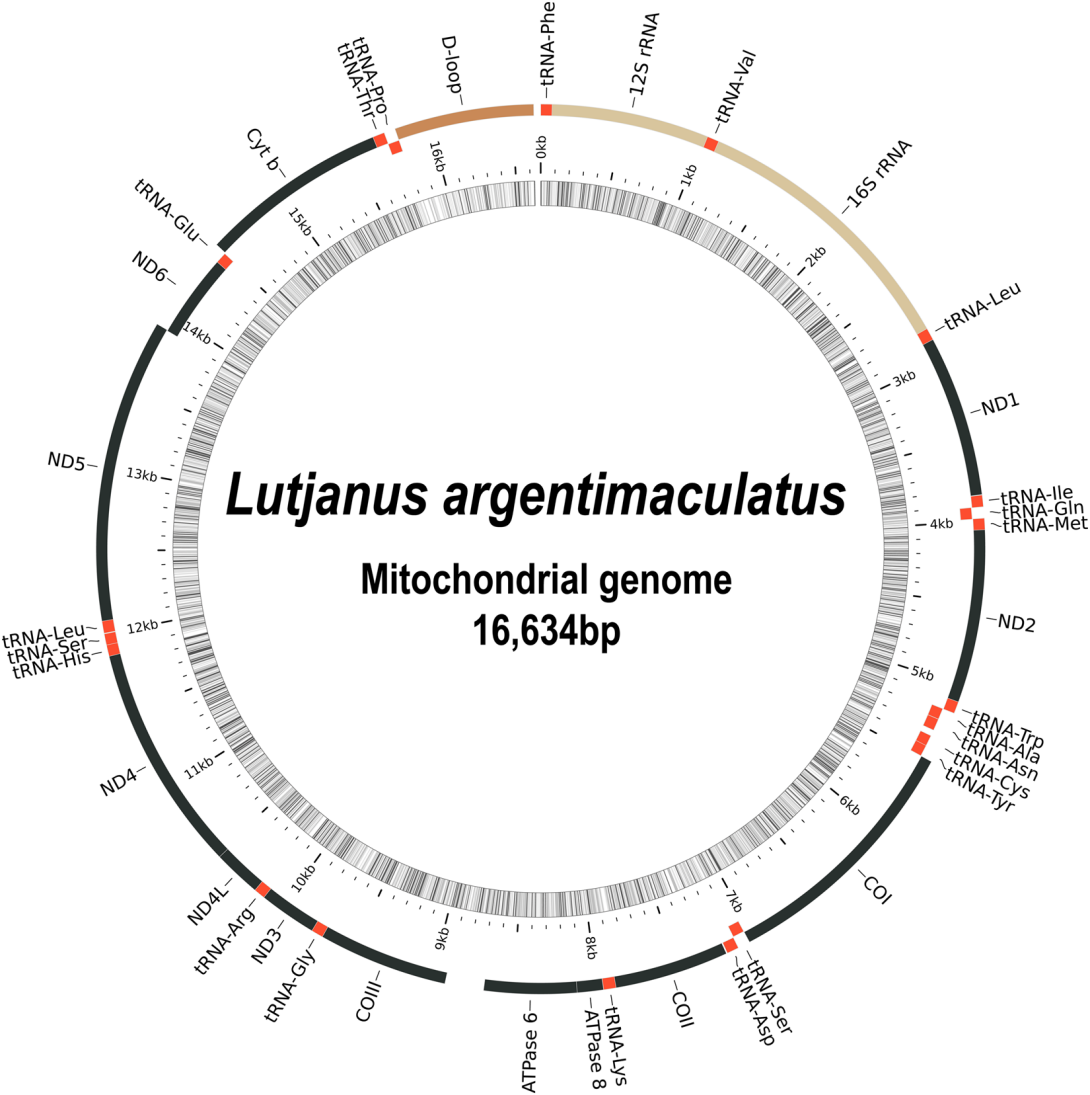
The circular mitochondrial genome map of *L. argentimaculatus* depicting the 13 encoded protein coding genes, 22 tRNA genes, 2 rRNA genes and a D-loop.

The contig level assembly of mangrove red snapper was generated using the long-read sequencing data of about 134 Gb using wtdbg2^15^, with minimum read length of 25,000 bp, and a minimum unitig length of 4096 bp. It generated an assembly of length 1.04 Gb in 699 contigs with N50 of 12.24 Mb. The mitochondrial DNA sequence was extracted from the contig file by performing blastn^16^ against known mitochondrial sequence of *L. argentimaculatus* (NCBI accession number: JN182927). The isolated mitochondrial genome sequence was 16,634 bp long and consisted of 13 CDS, 22 tRNA and 2 rRNA coding genes and a D-loop (Fig. 4). The sequence was uploaded to NCBI database along with the genome and was assigned the accession number CM068474. The contig assembly was further processed for removal of haplotigs and contig overlap using Purge_Dups v1.2.6^17^. This resulted in 382 contigs with an N50 value of 12.24 Mb and total length of 1.03 Gb.

**Fig. 5:**
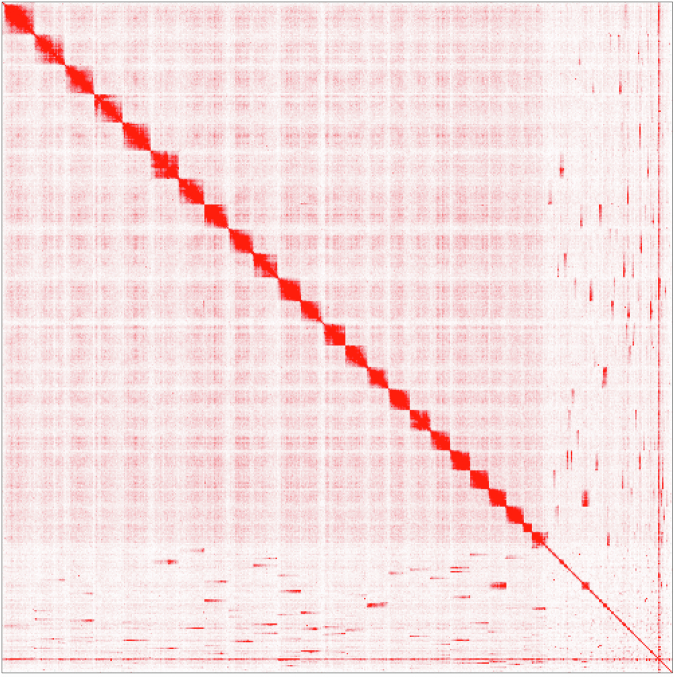
Hi-C map representing the scaffolds of Redsnapper genome assembly.

**Fig. 6:**
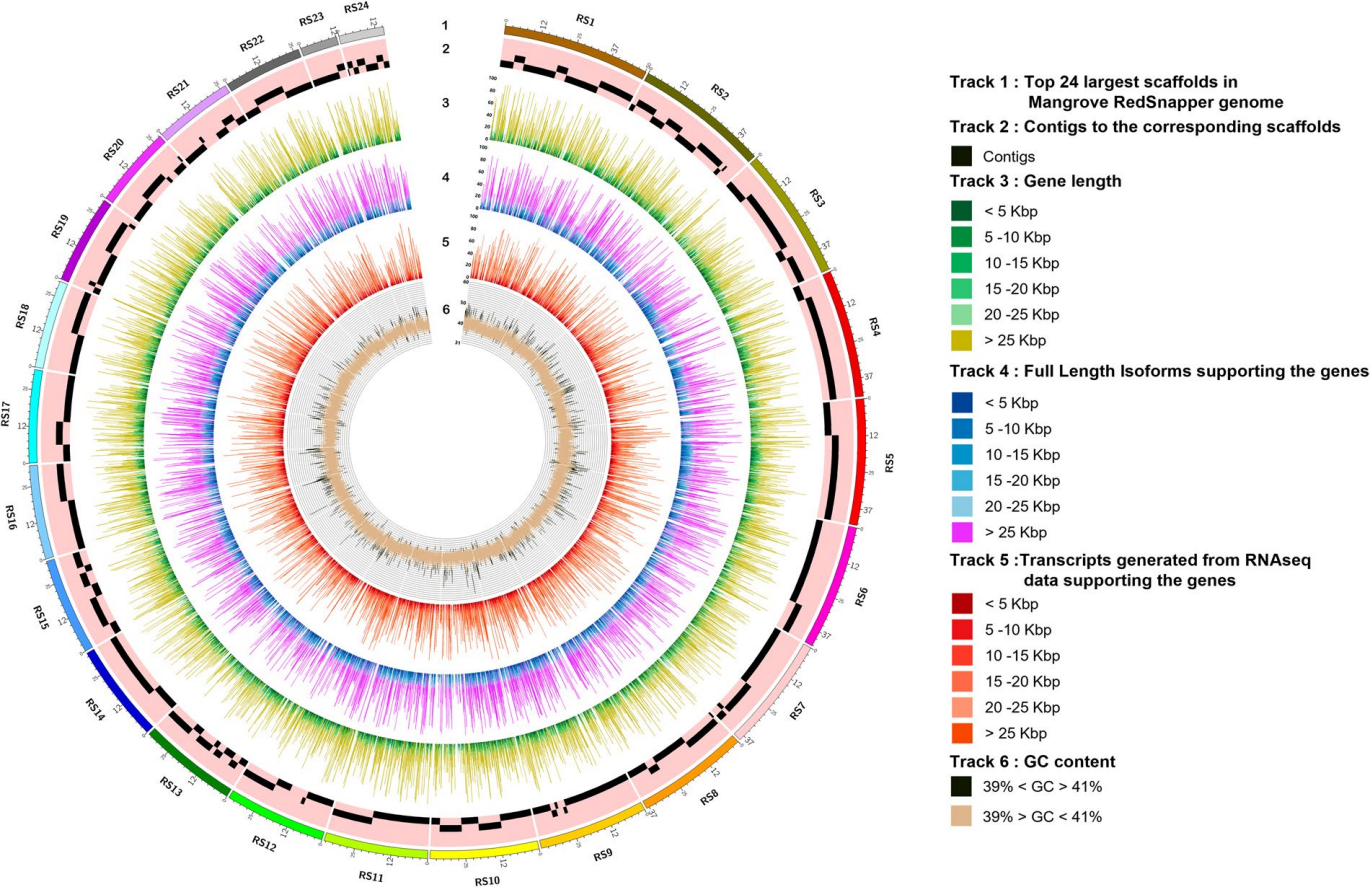
Circos plot of the Mangrove red snapper genome. From the outermost: Track1: Top 24 largest scaffolds of the red snapper genome. Track2: Contigs corresponding to the scaffolds represented as tiles. Track3: Genes of red snapper shown as highlights with incremental gene lengths of 5,000 bp (viz. <5 kb, 5 kb - 10 kb, 10 kb - 15 kb, 15 kb - 20 kb, 20 kb - 25 kb and >25 kb) shown in ascending order of the gene lengths. Track4: Full length isoform sequences supporting the genes of red snapper shown as highlights with incremental isoform lengths of 5,000 bp (viz. <5 kb, 5 kb - 10 kb, 10 kb - 15 kb, 15 kb - 20 kb, 20 kb - 25 kb and >25 kb) shown in ascending order of thethe isoform lengths. Track5: Transcripts generated from RNAseq data supporting the genes of red snapper shown as highlights with incremental transcript lengths of 20,000 bp (viz. <5 kb, 5 kb - 10 kb, 10 kb - 15 kb, 15 kb - 20 kb, 20 kb - 25 kb and >25 kb) shown in the ascending order of the transcript lengths. Track6: GC content of Red snapper genome shown as line diagram plotted with 100 kb sliding window. The GC values below 35 and above 45 are shown in black color, and remaining in tan color.

The purged contig assembly was further scaffolded with long range Hi-C contact map data using the tool SALSA2^18^. Briefly the paired end reads were trimmed using fastp v0.12.4^19^ with the option, --detect_adapter_for_pe. Trimmed reads were mapped to the purged contig assembly using BWA-MEM v 0.7.17-r1188^20^ software and the resultant *sam* file was converted to *bam* file format. Further this *bam* file is converted to *bed* format using bamToBed program of bedtools v2.27.1^21^. Finally, the sorted *bed* file, restriction enzyme cut sites (GATC, CTNAG, TTAA and GANTC) and the purged contig assembly was given as inputs to the SALSA2 pipeline to build the scaffold level assembly. The assembly file and Hi-C file were loaded on Juicebox with Assembly Tools v.2.17.00 for visualization and manual curation (Fig. 5). The scaffold level assembly comprised of 400 scaffolds with the top 24 scaffolds covering up to 828.87 Mb (Fig. 6, Table 1). The genome size estimated for *L. argentimaculatus* by flow cytometry indicated a closer approximation of the actual size (1.03 Gb) of the mangrove red snapper genome assembled in this study.

**Table 1:** Assembly statistics of *L. argentimaculatus* genome at contig and scaffold level.

Parameter	Scaffold Assembly	Primary Contig Assembly after Purging	Primary Contig Assembly
No. of sequences	400	382	699
Longest sequence, bp	50,306,003	38,328,485	38,328,485
Total length	1,030,266,718	1,030,280,882	1,042,747,252
N50, bp	33,807,785	12,244,877	12,244,877
N75, bp	27,147,058	5,735,490	5,597,631
L50	13	28	28
L75	21	57	59
GC (%)	39.58	39.58	39.6

**Fig. 7:**
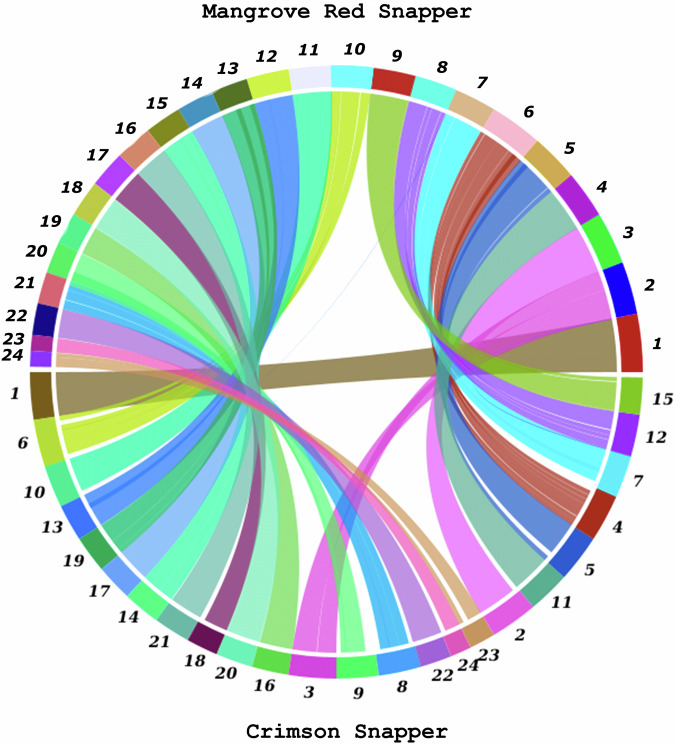
Synteny between Mangrove red snapper and Crimson snapper.

Synteny analysis was performed for the assembly of mangrove red snapper with crimson snapper, *Lutjanus erythropterus* [Assembly accession no. ASM2009168v1]^22^ and zebrafish, *Danio rerio* [Assembly accession no. GRCz11]^23^ using SyMAP v5.3.5^24^ to understand the genome similarity between them. The largest 24 scaffolds of red snapper and crimson snapper were taken, whereas for the zebrafish, 25 chromosomes were taken to perform genomic synteny. The analysis revealed that there are 31 syntenic blocks greater than 10 mb between mangrove red snapper and crimson snapper (Fig. 7). Whereas when the red snapper was compared to the zebrafish there are 8 and 58 blocks greater than 10 mb shared between them respectively (Fig. S1). It was also observed that 99% of red snapper is covered by 84% of crimson snapper in syntenic blocks. Similarly, 96% of red snapper genome is covered by 92% of zebrafish genome in syntenic blocks.

### Repeat profile

The repeat profile of the red snapper assembly was obtained using RepeatMasker v4.1.4^25^ (http://repeatmasker.org/RMDownload.html). First the database of repeat families were generated using RepeatModeler v2.0.4 (http://repeatmasker.org/RepeatModeler/) and then *de novo* repeat prediction was done using RepeatMasker v 4.1.4, LTR_FINDER (LTRPipeline v2.0.4), and TRF tool v4.09^26^ to identify all kinds of repetitive elements.

A total of 446.78 Mb genome sequences were identified as repeats, accounting for 43.37% of the whole assembly. DNA transposons constituted the major classified repeat class (13.51%, 139,201,547 bp), followed by Retroelements (5.06%, 52,120,576 bp). Simple repeats (1.81%, 18,645,993 bp) and rolling circles (0.45%, 4,654,588 bp), The major repeat element classes of retroelements was found to be LINEs (3.28%) followed by LTR elements (1.44%), SINEs (0.34%) and Penelope (0.05%). The Low complexity sequences were found be around 0.26% and small RNAs were estimated to be around 0.23%. (Table 2).

**Table 2:** Repeat profile of *L. argentimaculatus* genome.

Sequences	400		
Total length	1,030,266,718 bp (1,030,255,618 bp excl N/X-runs)		
GC level	39.58%		
Bases masked	446,783,797 bp (43.37%)		
**Repeat type**	**Number of elements**	**Length occupied**	**Percntage of sequences**
**Retroelements**	**206673**	**52120576 bp**	**5.06%**
SINEs:	27131	3536449 bp	0.34%
Penelope	320	501991 bp	0.05%
LINEs:	124695	33786881 bp	3.28%
L2/CR1/Rex	81348	18211440 bp	1.77%
R1/LOA/Jockey	4787	1307566 bp	0.13%
R2/R4/NeSL	1185	636861 bp	0.06%
RTE/Bov-B	10284	3495319 bp	0.34%
L1/CIN4	12433	4091338 bp	0.40%
LTR elements:	54847	14797246 bp	1.44%
BEL/Pao	2245	1432351 bp	0.14%
Ty1/Copia	177	85862 bp	0.01%
Gypsy/DIRS1	6428	4103204 bp	0.40%
Retroviral	6647	2042098 bp	0.20%
**DNA transposons**	**808300**	**139201547 bp**	**13.51%**
hobo-Activator	428796	64443905 bp	6.26%
Tc1-IS630-Pogo	60027	14417083 bp	1.40%
MULE-MuDR	2968	479205 bp	0.05%
PiggyBac	2908	702620 bp	0.07%
Tourist/Harbinger	38194	7729754 bp	0.75%
Other (Mirage, P-element, Transib)	19176	3396650 bp	0.33%
**Rolling-circles**	**9696**	**4651588 bp**	**0.45%**
**Unclassified:**	**1885896**	**228268275 bp**	**22.16%**
**Total interspersed repeats:**		**420092389 bp**	**40.78%**
**Small RNA:**	**16832**	**2333510 bp**	**0.23%**
**Satellites:**	**5393**	**617252 bp**	**0.06%**
**Simple repeats:**	421814	**18645993 bp**	**1.81%**
**Low complexity:**	48463	**2726203 bp**	**0.26%**

### Iso-Seq transcriptome data processing

The raw data was processed using iso-seq3 pipeline SMRTLink v10.1 to obtain high quality isoforms. The methodology described in^27,28^. was followed with minor modifications. Briefly, *ccs* step was called to obtain Circular Consensus Sequence (CCS) with options as ‘minimum passes = 3 and minimum quality = 0.99’. The data was further demultiplexed using *lima* by providing the options ‘peek-guess, dump-clips, and dump-removed’. The demultiplexed sequences were further processed through isoseq3 *refine* step with options ‘require-polya and minrq = 1’ to obtain Full Length Non-Chimeric sequences (FLNC). Finally, isoseq3 *cluster* was run to obtain 94,258 high quality transcripts with N50 length of 3,973 bp. To screen for any possible contaminants Mash v2.2^29^ was used with the refseq genome sketch. To reduce the redundancy in transcripts, the high quality sequences were collapsed based on the exonic structures using the isoseq3 *collapse* program with options ‘min-aln-coverage = 0.85 and min-aln-identity = 0.95’ to obtain 56,515 non-redundant transcripts.

### Gene prediction and functional annotation

Genes were predicted for the mangrove red snapper genome by incorporating evidences from Iso-seq, RNAseq datasets and proteins from related species. The protocol described in^30,31^ was adapted with minor changes. While the evidence from Iso-seq data was directly used for building the gene models, the other two evidences, RNA-seq and proteins were used to refine the gene models. Briefly, the full-length non-chimeric (FLNC) sequences of Iso-Seq data generated in this study were aligned to the genome using GMAP v2020-06-30^32^ to generate hints. Tentative genes were predicted using Augustus v3.3.3^33^ by providing the repeat masked genome and the Iso-seq hints generated previously as inputs and zebrafish gene models as reference. The evidence from RNA-seq data were obtained by aligning paired-end reads of various tissues (gills, muscle, kidney, stomach, gonad, and liver) to the genome using Hisat2 v2.2.0^34^ and the resultant *sam* file was converted to *bam* file format and sorted using samtools v1.14^35^. The reference based transcriptome was assembled using StringTie v2.1.4^36^ by providing the sorted *bam* file as input. These assembled transcripts were subjected to further refinement, filtering and subsequently transformed into genome-based coordinates using TransDecoder v5.5.06^37^. Additionally, protein sequences from closely related species within the order Eupercaria (Supplementary Table 6) were downloaded from NCBI Genome database, concatenated and subjected to clustering at 90% identity threshold using CD-HIT V4.8.1^38^. Hints were then generated by aligning these clustered protein sequences to the genome using GenomeThreader v1.7.3^39^.The gene model, formed by integrating the outputs of Augustus with hints from Iso-Seq, RNA-Seq, and homologous proteins, was unified using a weighted approach within Evidence Modeler^37^. This approach culminated in a consensus gene model encompassing a total of 27,172 genes comprising of 21,629 complete genes (having start and stop codons) and 905 genes with neither start nor stop codons (Supplementary Table S7).

A homology based annotation against the Actinopterygii (txid7898) dataset of non-redundant database was performed using blastx tool^16^ and hits were obtained for 24,981 (91.93%) transcripts, The protein domains based annotation was executed by the Interproscan module of OmicsBox v3.2.4^40^ and the EggNOG mapper module^41^ was used for orthology based annotation resulting in 22,870 (84.17%) and 16,147 (59.42%) hits respectively. The gene ontology and functional annotation obtained by combining the gene ontology terms from blastx, Interpro and EggNOG summed upto 22,491 (82.77%) transcripts. The annotated genes participating in different pathways were obtained by mapping against KEGG database^42^ and enzyme codes were obtained for 7,467 (27.48%) of them (Table 3) Figs. S2 & S3. Transferases and hydrolases were the most dominant enzyme classes expressed (Fig. S4). Gene ontology revealed that the most expressed GO categories were macromolecule biosynthetic process (Biological processes), anion binding (Molecular function) and intracellular membrane-bounded organelle (Cellular components) (Fig. S5). The annotated genes were involved in 330 pathways as per the results obtained from KEGG pathway analysis (**refer Gene_pathways.xlsx**^43^ **in figshare repository**).

**Table 3:** Annotation statistics of *L. argentimaculatus* through various databases.

Attributes	No. of Transcripts
Predicted Genes	27,172
Blast Hits	24,981 (91.93%)
InterProScan hits	22,870 (84.17%)
EggNOG hits	16,147 (59.42%)
GO Annotation	22,491 (82.77%)
GO Mapping	10,229 (37.64%)
Enzyme code	7,467 (27.48%)
No hits	2,191 (8.06%)

### Isoform diversity of *L. argentimaculatus* transcriptome

SQANTI3 v. 5.0^44^ was used to classify 56,515 transcripts into isoform categories and it has led to identification of 18,108 unique gene models in the mangrove red snapper transcriptome (Table 4, Supplementary file 1). Out of 56,515 unique isoforms, 10.6% isoforms were found to be Full Splice Match (FSM), since they are found to have the same splice junctions as the reference transcripts. The splice junction matching the reference from the beginning but not at the end and called as Incomplete Splice Match (ISM) were found to be 3.75% of the total isoforms. The Novel in Catalog (NIC) having a combination of known acceptor or donor splice sites and Novel not in Catalog (NNC) which are known to use at least one novel acceptor or donor sites were found to be at 2.6% and 43.94% respectively. Another major category of isoforms which typically span across two genes and called as Fusion isoforms comprised of 32.46%. The genic genomic isoforms which overlap with introns and exons comprised of 1.69%. The intergenic isoforms which are found in intergenic locations were about 4.49%. The antisense isoform which have an overlap in the antisense strand were about 0.4% in the mangrove red snapper transcriptome. The Genic intron isoform which are known to be completely contained within an intron category of isoforms were absent in the mangrove red snapper transcriptome. About 43.69% of genes had only one isoform and about 27.42% genes had two to three isoforms. About 13.53% of genes consisted of four to five isoforms and rest of 15.35% genes had more than or equal to 6 isoforms (Fig. S6).

**Table 4:** Statistics of Iso-Seq based transcriptome analysis.

Feature	Count
Iso-Seq data generated, bases	138,561,629,489
Number of CCS reads	2,841,798
Number of FLNC sequences	2,837,244
Number of high-quality transcripts	94,258
N50 length, bases	3,973
Number of unique isoforms	56,515
Number of unique genes	18,108
Unique genes With Blast Hits	17,638
Unique genes With GO Annotation	12,070

We have obtained high number of 56,515 unique isoforms which was comparable with equally high number of isoforms (66538) obtained from grey mullet in our earlier study^27^. A higher proportion of genes with more isoforms indicate presence of a high degree of transcriptome complexity in brackishwater fish transcriptomes. These transcriptomes will be an important resource in exploring the overall contribution of alternative isoforms to gene expression related to various functional pathways.

### Alternate splicing events and rarefaction curve analysis

**Fig. 8:**
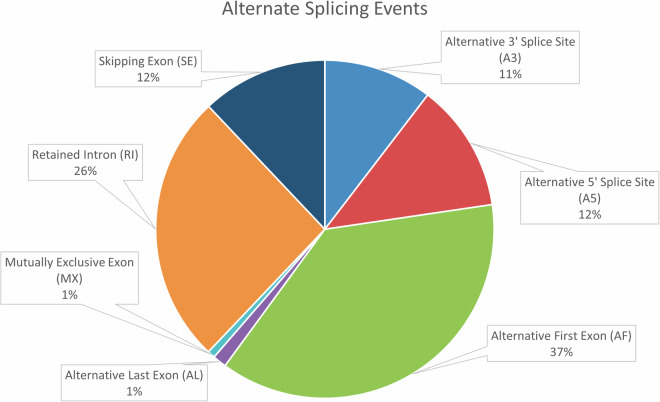
Identification of alternate splicing events in *L. argentimaculatus* transcriptome.

For identifying the alternate splicing events in the mangrove red snapper transcriptome, SUPPA2^45^ with the subcommand *generateEvents* was used with default options. In total 23,064 alternate splicing events were identified in 5,905 genes, of which 5,228 were alternative 3′ splice site and alternative 5′ splice site (A3/A5 events), 8,925 were alternative first/last exons (AF/AL events), 5,964 were retained intron (RI events), 2,780 were Skipping Exon SE events and 167 were mutually exclusive exons (MX events) (Fig. 8).

Alternate splicing plays key roles in response to different stress factors in the fish such as aggression in *Betta splendens*^46^ cold stress in Tilapia^47^, heat stress in catfish^48^. The other functional associations of alternative splicing include in sex determination and gonadal differentiation reported in zebra fish^49^, antiviral immunity in grass carp^50^ etc. It has been reported that alternate splicing events show universally similar trends across organisms with relatively high occurrences of retained introns (RIs), alternative 3′ and 5′; splice sites (A3 and A5), alternative first exons (AF), and less degree of skipping exons (SE), alternative last exons (AL) and mutually exclusive exons (MX). Our study also revealed similar pattern except for AF which showed highest events (37%), followed by RIs (26%). The SE (12%), AL and MX (1%) were observed at lower levels. However, the comparative pattern of alternate splicing events in case of fishes alone remains to be studied before any conclusion.

**Fig. 9:**
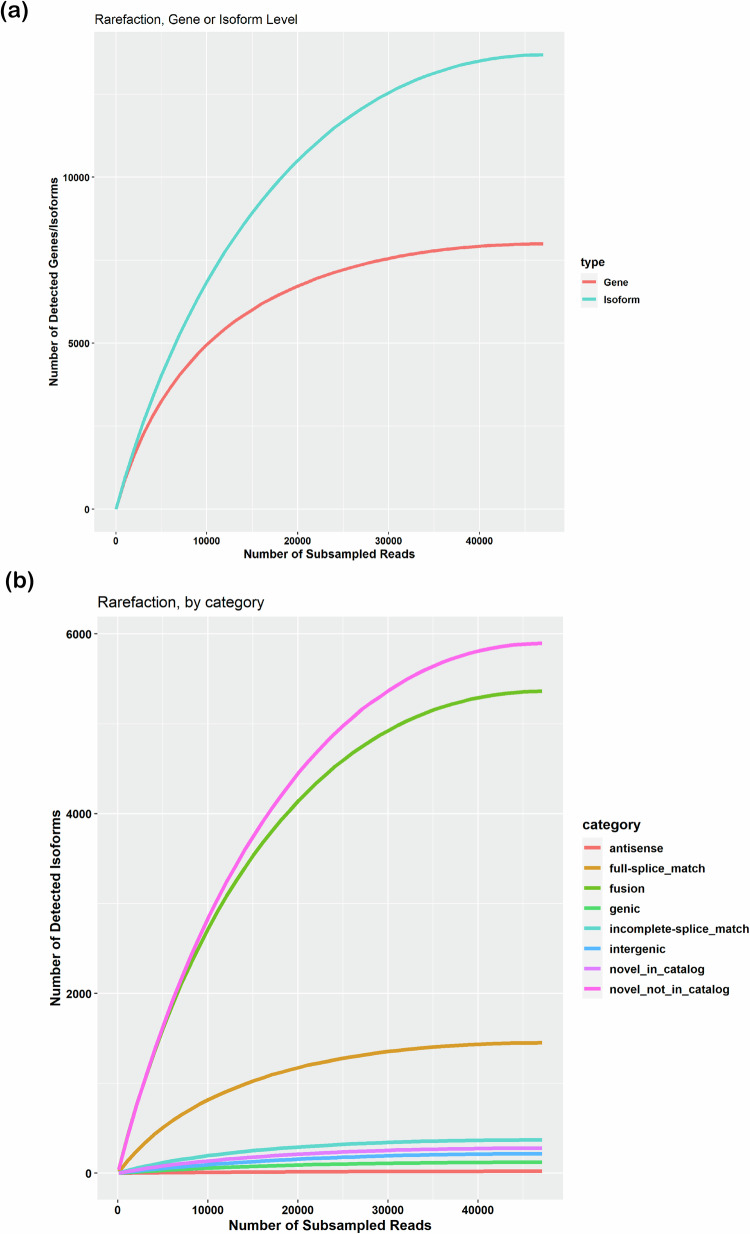
The rarefaction curve analysis. **(a)** to obtain genes and isoforms relative to the read depth, **(b)** the isoform discovery rate at the various categories level.

Rarefaction curve analysis was performed to understand the read depth required for discovery of genes and isoforms. From the cDNA_Cupcake scripts (https://github.com/Magdoll/cDNA_Cupcake), *subsample.py* was used with the options ‘min_fl_count = 2 a– --step = 1,000’ by providing the collapsed transcripts and the sqanti3 classification file as inputs. To understand further about the isoform discovery rate at the various categories level, another script *subsample_with_category.py* was used with the options and inputs same as subsample.py script. The rarefaction curve analysis represented the relation between genes and isoforms to the read depth (Fig. 9(a)) and the isoform discovery rate at the various categorical levels (Fig. 9(b)). At the gene/isoform level, rarefaction curves showed that the discovery of genes reached saturation much earlier in comparison to the isoforms. Under isoform categories, the novel not in catalog (NNC) and fusion isoforms are still being discovered, whereas the other categories of isoforms reached saturation and showed sequencing depth sufficiency.

In conclusion, this highly contiguous genome assembly and the isoform characterized transcriptome of *L. argentimaculatus* will be a useful genetic resource and will provide valuable insights and information pertaining to structural, functional, and evolutionary aspects of fish.

## Data Records

The datasets generated during and analysed during the current study are available in the NCBI’s Bioproject repository under the accession number PRJNA1036849^51^. The genome sequence was submitted to NCBI Genome Assembly database under the accession number GCA_034769285.1 and figshare data repository(Genome.fasta)^43,52^. The Table 5 lists the biosample and SRA accession numbers of the data. The Isoseq assembly transcript^43^ and the predicted genes sequence^43^ files are available in the figshare data repository.

**Table 5:** List of data generated in the current study and their corresponding accession numbers.

S. No	Data Type	Tissue	Biosample Accession	SRA Accession
1	Pacbio Long reads (Genomic Data)	Muscle	SAMN38153217	SRR26856359^55^
2	Pacbio IsoSeq (Transcriptomic Data)	Pooled (Muscle, gills, stomach, liver, kidney, gonad)	SAMN38196717	SRR26856358^56^
3	Linked Reads (Hi-C data)	Muscle	SAMN38153217	SRR26872073^57^
4	Illumina Short reads (Genomic data)	Muscle	SAMN38153217	SRR26872074^58^
5	Illumina Short reads (RNASeq)	Muscle	SAMN38153217	SRR26872072^59^
6		Stomach	SAMN38169620	SRR26872067^60^
7		Gills	SAMN38169618	SRR26872069^61^
8		gonad	SAMN38169619	SRR26872068^62^
9		kidney	SAMN38169617	SRR26872070^63^
10		liver	SAMN38169616	SRR26872071^64^
11	Genome Assembly	NCBI Genome database	Accession Number: GCA_034769285.154 10.6084/m9.figshare.25107587.v2^44^	
12	Contig Assembly (Purged)	Figshare data repository	10.6084/m9.figshare.25107587.v2^44^	
13	Isoseq Assembly	Figshare data repository	10.6084/m9.figshare.25107587.v2^44^	
14	Predicted genes	Figshare data repository	10.6084/m9.figshare.25107587.v2^44^	

## Technical Validation

**Fig. 10:**
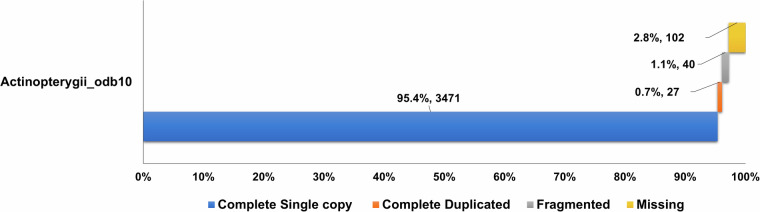
Genome completeness analysis using BUSCO for *L. argentimaculatus* scaffolds against Actinopterygii_odb10 orthologous database.

The genome completeness of the assembled final scaffolds were assessed using BUSCO v5.3.2 tool^53^ utilizing the actinopterygii_odb10 ortholog database for assessment. BUSCO assessment confirmed genome completeness of 97.20% (3495 orthologs), which includes a 95.4% (3471 orthologs) of complete single copy orthologs, 0.7% (27 orthologs) of complete duplicate orthologs, 1.1% (40 orthologs) of fragmented orthologs, and the missing orthologs contributed to the remaining 2.8% (102 orthologs). (Fig. 10). Further validation of the assembled genome was performed by mapping the short reads to the assembled genome. The mapping was performed by using BWA-MEM v 0.7.17-r1188^20^ to generate the *sam* files and then converted and sorted into bam files. Qualimap^54^ tool was used to assess the *bam* file and generate the QC report, which suggested that 99.41% of the reads were mapped back onto the red snapper genome assembly. Similarly RNASeq data was also combined and mapped to the genome assembly of red snapper using the Hisat2 tool to generate the *sam* file and subsequently converted to and sorted to *bam* file. The QC report generated by qualimap tool showed that 74.17% of the reads were mapped back to the genome assembly.

One of the criteria to assess the quality and accuracy of a genome assembly is to compare the assembly and annotation metrics with those of closely related species. The current assembly of *L. argentimaculatus* genome is of high quality as compared to the published genomes for other Lutjanus species (*L. campechanus* and *L. erythropterus*). While the assembly reported by Norrell *et al*. for *L. campechanus* genome spanned about 770 Mb with 76,321 contigs (N50 = 14,414 bp) and 67,254 scaffolds (N50 = 16,803 bp) were obtained. The *L. campechanus* was also assembled by Portnoy *et al*. with improved metrics, genome length 1.2 Gb consisting of 140,690 scaffolds with N50 of 20,750 bp. Though the genome length and scaffold N50 has improved, the number of scaffolds still remain high for *L. campechanus*. The assembly of *L. erythropterus* was found to have better scaffold level metrics despite a modest level assembly. It has genome length of 969,885,659 bp consisting of 10,853 scaffolds (N50 = 40,645,435 bp) (Supplementary Table 8). In comparison for the *L. argentimaculatus* genome, to improve the genome assembly by increasing the genome coverage and contiguity, we used Pacific Biosciences (PacBio) Single Molecule Real-Time (SMRT) technology in this study to generate 134.6 Gb sequence data. The high-quality genome assembly of *L. argentimaculatus* yielded a smaller number of contigs (382) and scaffolds (400) with high N50 values of 12 Mb and 33.8 Mb respectively indicating the outstanding contiguity metrics obtained. This was further substantiated by BUSCO metrics on confirming genome completeness of 97.20%, against Actinopterygii_odb10 orthologous database.

### Supplementary information

Supplementary Figure

Supplementary Tables

Supplementary File

## Data Availability

All data processing commands and pipelines were carried out in accordance with the instructions and guidelines provided by the relevant bioinformatics software. There were no custom scripts or code utilized in this study.
