# Supplementary material for: Genome assembly, Full-length transcriptome, and isoform diversity of Red Snapper, Lutjanus argentimaculatus
Source: Sci Data. 2024 Jul 18;11:796. doi: 10.1038/s41597-024-03633-1 (PMC11258364; doi:10.1038/s41597-024-03633-1)
Supplement: Supplementary file 1 — Supplementary Figure [file 41597_2024_3633_MOESM1_ESM.docx]

**Genome assembly, Full-length transcriptome, and isoform diversity of Red Snapper, Lutjanus argentimaculatus**

Mudagandur S Shekhar^1^, Vinaya Kumar Katneni^1,^*, Ashok Kumar Jangam^1^, Karthic Krishnan^1^, Sudheesh K Prabhudas^1^, Roja Jayaraman^1^, Jesudhas Raymond Jani Angel^2^, Muniyandi Kailasam^3^

^1^ Centre for Bioinformatics, ICAR-Central Institute of Brackishwater Aquaculture, No 75, Santhome High Road, MRC Nagar, Chennai, 600028, Tamil Nadu, India

^2^ Crustacean Culture Division, ICAR-Central Institute of Brackishwater Aquaculture, No 75, Santhome High Road, MRC Nagar, Chennai, 600028, Tamil Nadu, India

^3^ Finfish Culture Division, ICAR-Central Institute of Brackishwater Aquaculture, No 75, Santhome High Road, MRC Nagar, Chennai, 600028, Tamil Nadu, India

^*^Corresponding Author: Vinaya Kumar Katneni, email: vinayciba@gmail.com


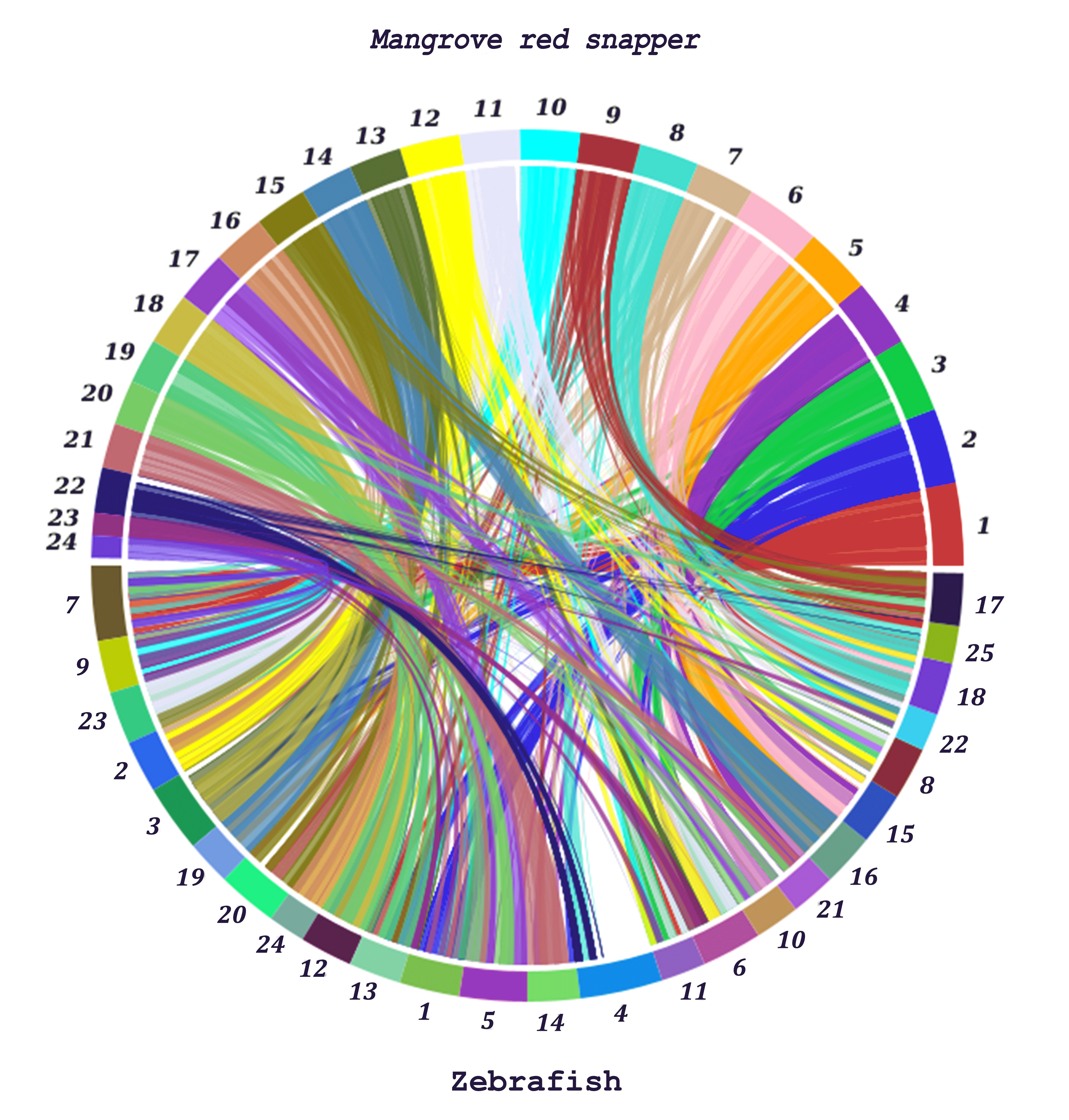


**Figure S1:** Synteny between Mangrove red snapper and Zebra fish


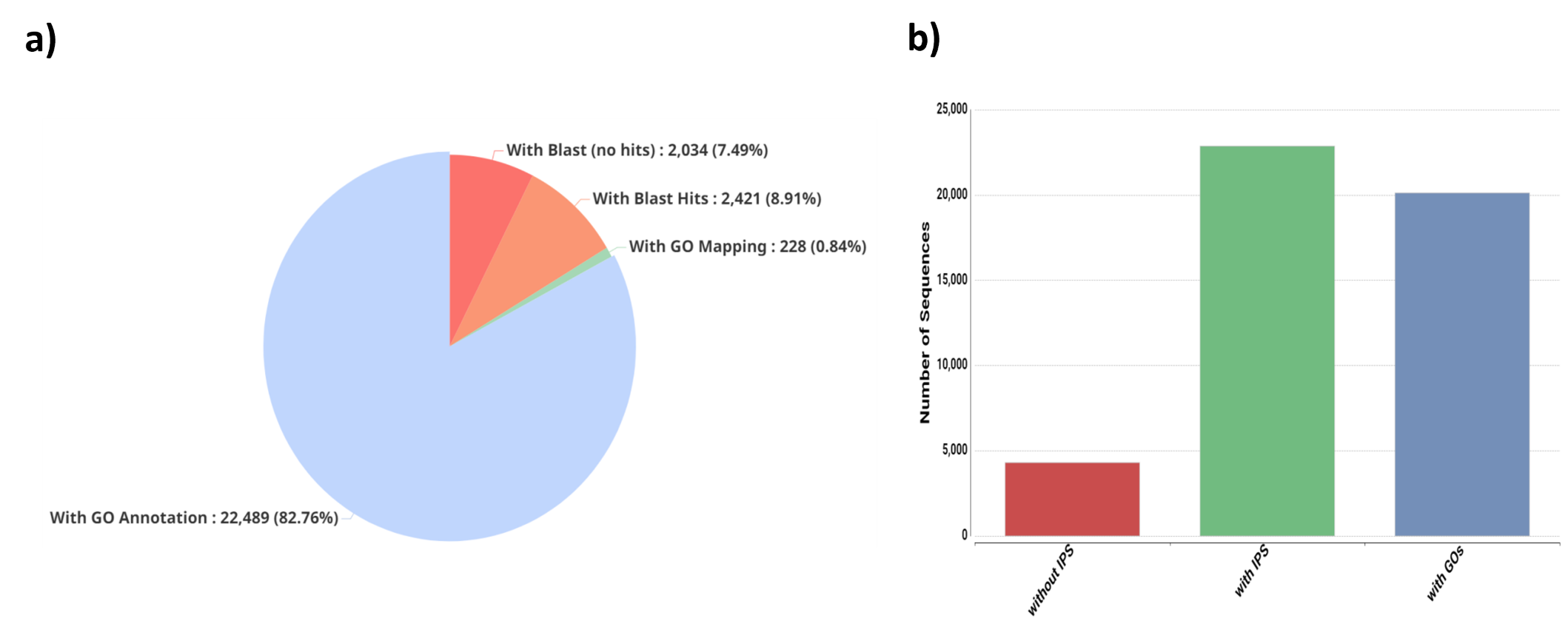


**Figure S2:** The distribution of transcripts at various functional annotation levels using Omicsbox tool. **a)** Pie chart depicting the percentage of transcripts with GO annotation, with only GO mapping, with only blast hits and No hits. **b)** Histogram showing the number of transcripts with Interproscan hits, without Interproscan hits and with Gene ontology ID’s.


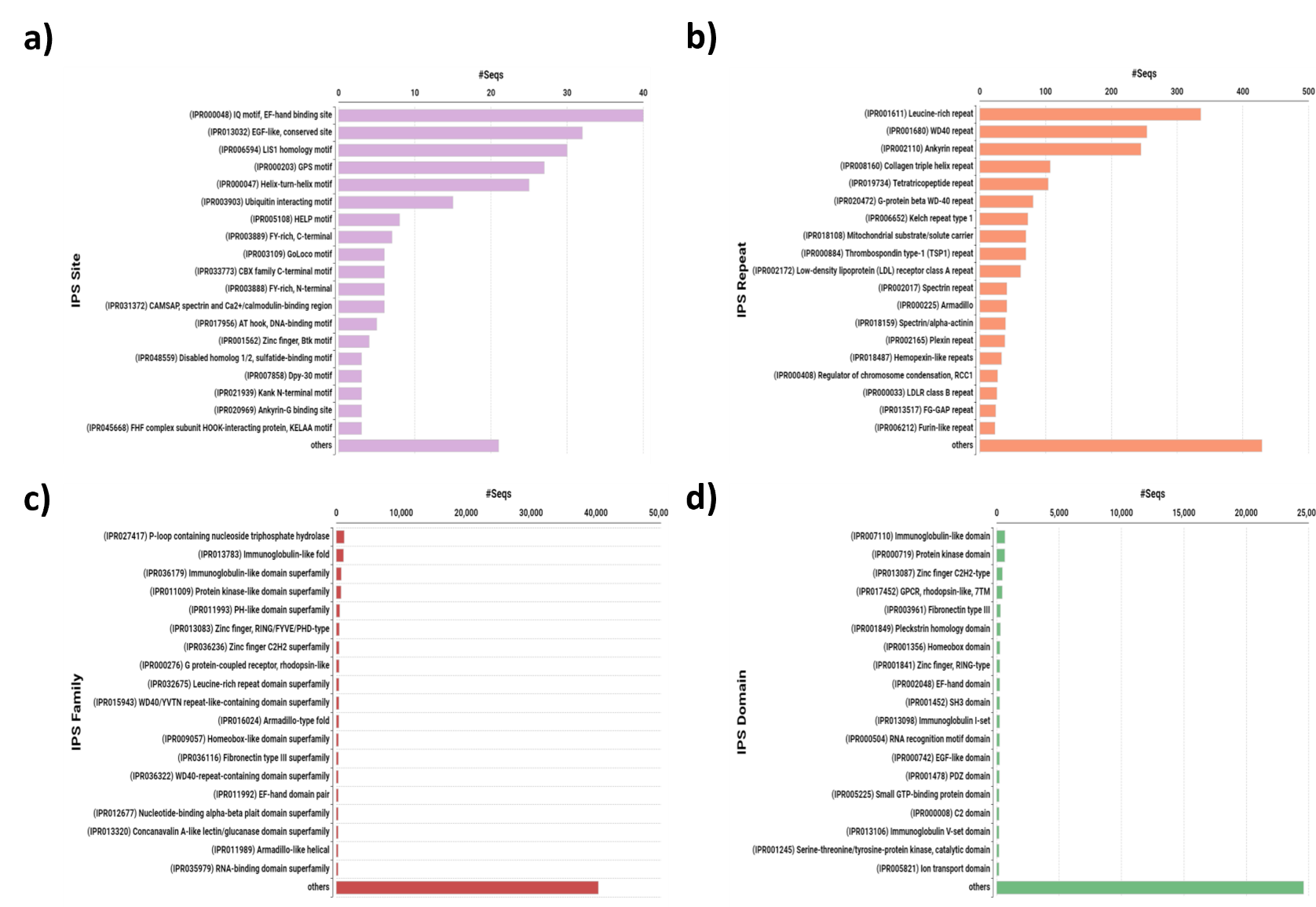


**Figure S3.** Distribution of transcripts based on different categories of Interproscan results. **a)** Distribution based on sites present on the transcript. **b)** Distribution based on repeats present in the transcripts. **c)** Distribution based on protein families. **d)** Distribution based on domains.


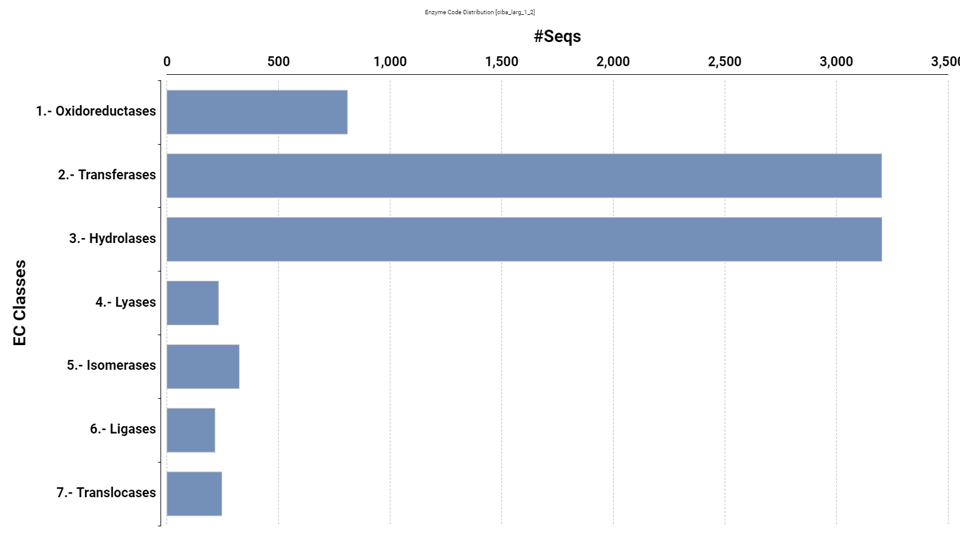


**Figure S4:** Histogram depicting the distribution of enzymes identified in the transcripts into different classes


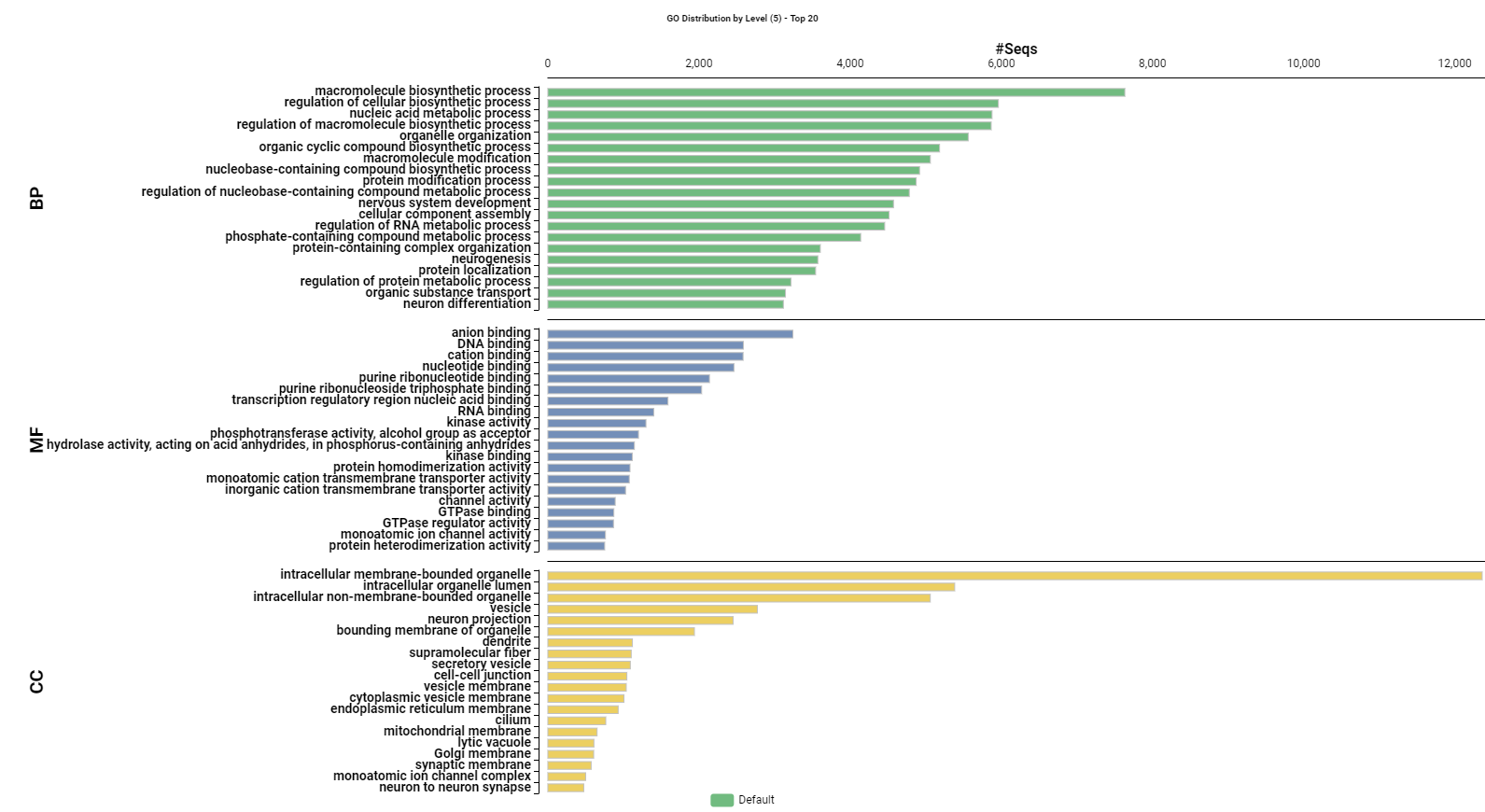


**Figure S5:** The Gene Ontology classification of the annotated genes by Level 5 of the predicted genes in *L. argentimaculatus* genome.


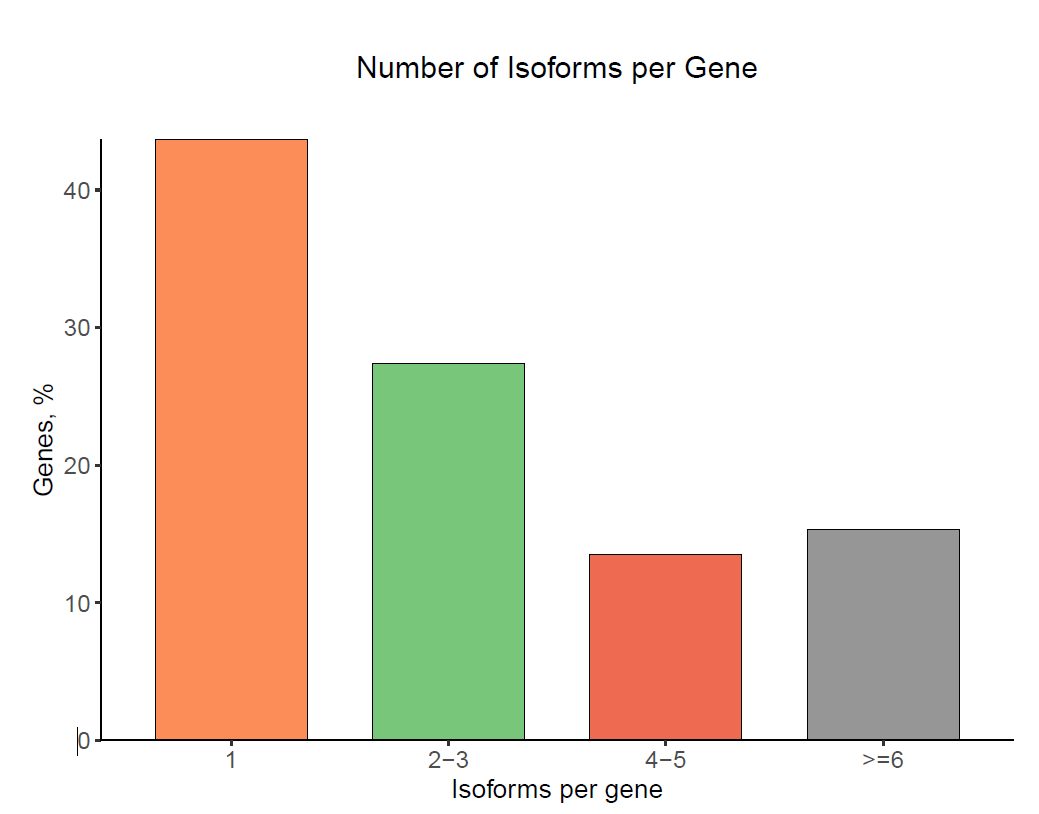


**Figure S6**: Isoform diversity distribution per gene.
