# Supplementary material for: Genome assembly, Full-length transcriptome, and isoform diversity of Red Snapper, Lutjanus argentimaculatus
Source: Sci Data. 2024 Jul 18;11:796. doi: 10.1038/s41597-024-03633-1 (PMC11258364; doi:10.1038/s41597-024-03633-1)
Supplement: Supplementary file 2 — Supplementary Tables [file 41597_2024_3633_MOESM2_ESM.docx]

**Genome assembly, Full-length transcriptome, and isoform diversity of Red Snapper, Lutjanus argentimaculatus**

Mudagandur S Shekhar^1^, Vinaya Kumar Katneni^1,^*, Ashok Kumar Jangam^1^, Karthic Krishnan^1^, Sudheesh K Prabhudas^1^, Roja Jayaraman^1^, Jesudhas Raymond Jani Angel^2^, Muniyandi Kailasam^3^

^1^ Centre for Bioinformatics, ICAR-Central Institute of Brackishwater Aquaculture, No 75, Santhome High Road, MRC Nagar, Chennai, 600028, Tamil Nadu, India

^2^ Crustacean Culture Division, ICAR-Central Institute of Brackishwater Aquaculture, No 75, Santhome High Road, MRC Nagar, Chennai, 600028, Tamil Nadu, India

^3^ Finfish Culture Division, ICAR-Central Institute of Brackishwater Aquaculture, No 75, Santhome High Road, MRC Nagar, Chennai, 600028, Tamil Nadu, India

^*^Corresponding Author: Vinaya Kumar Katneni, email: vinayciba@gmail.com

**Table S1**: Species and accessions used for phylogenetic analysis of *L. argentimaculatus* using the COI sequence for species identification.

| **Species** | **No. of Accessions** |
| --- | --- |
| *Lutjanus argentimaculatus* | 39+1^*^ |
| *Lutjanus bohar* | 23 |
| *Lutjanus gibbus* | 37 |
| *Lutjanus johnii* | 28 |
| *Lutjanus lutjanus* | 65 |
| *Lutjanus fulviflamma* | 67 |
| *Lethrinus lentjan* (out group) | 3 |
| **Total** | **263** |

* 39 accessions from Bold database and 1 is sample sequence used in this study

**Table S2**. Raw read metrics of the Pacbio CLR sequencing for *L. argentimaculatus*.

| **Attributes** | **Value** |
| --- | --- |
| Polymerase Read Bases | 134,676,716,511 |
| Polymerase Reads | 7,196,502 |
| Polymerase Read Length (mean) | 18,714 |
| Polymerase Read N50 | 29,027 |
| Subread Length (mean) | 15,808 |
| Subread N50 | 24,615 |
| Longest Subread Length (mean) | 16,314 |
| Longest Subread N50 | 25,201 |
| Unique Molecular Yield | 117,289,975,808 |

**Table S3**: Statistics of Hi-C raw reads generated for scaffolding the contigs of *L. argentimaculatus*

| Total read pairs (RPs) analyzed | 224,819,705 |
| --- | --- |
| Total Reads | 449,639,410 |
| Total bases (Gb) | 67.446 |
| Q30 bases (Gb) | 62.005 (92.49%) |
| Q20 bases (Gb) | 63.821 (97.09%) |
| Clustering usable HQ reads per contig (CTGs >5KB) | 1162.12 |
| RPs >10KB apart | 2.34% |
| RPs >10KB apart (CTGs >10KB) | 9.97% |
| Intercontig RPs | 13.76% |
| Intercontig HQ RPs | 16.88% |
| Same strand RPs | 6.82% |
| Split reads | 5.43% |

**Table S4:** The transcriptome library quality and sequence data stats for *L. argentimaculatus*

| **Sample** | **Quantity (ng/ul)** | **Insert size (bp)** | **Total Reads** | **Total Bases** |
| --- | --- | --- | --- | --- |
| Gills | 1.5450 | 346 | 122,358,582 | 18,476,145,882 |
| Kidney | 0.8949 | 314 | 120,764,058 | 18,235,372,758 |
| Liver | 1.0616 | 344 | 115,361,278 | 17,419,552,978 |
| Muscles | 0.7241 | 295 | 113,063,266 | 17,072,553,166 |
| Stomach | 1.7055 | 362 | 152,791,934 | 23,071,582,034 |
| Gonad | 0.467 | 317 | 96342858 | 14,547,771,558 |

**Table S5**: Metrics of PacBio- Iso-Seq sequencing data

| **Attributes** | **Data** |
| --- | --- |
| Polymerase Reads | 1,945,300 |
| Subreads | 42,641,710 |
| Bases | 138,561,629,489 |
| Mean Read Length | 73,714 |
| Mean of Longest Subread Length | 4,946 |
| Unique Molecular Yield | 7,078,415,047 |

**Table S6**: List of species, whose protein sequences were used for gene prediction.

| **Organism Scientific Name** | **Organism Common Name** | **Assembly Name** | **Assembly Accession** | **Level** | **Contig N50** | **Size** | **Gene Count** | **BioProject** | **BioSample** |
| --- | --- | --- | --- | --- | --- | --- | --- | --- | --- |
| *Takifugu rubripes* | torafugu | FUGU5 | GCF_000180615.1 | Chromosome | 52,883 | 3,914,68,268 | 23,151 | PRJNA1434 | SAMEA3138310 |
| *Larimichthys crocea* | large yellow croaker | L_crocea_2.0 | GCF_000972845.2 | Chromosome | 277,487 | 657,923,191 | 27,392 | PRJNA245366 | SAMN03092871 |
| *Perca flavescens* | yellow perch | PFLA_1.0 | GCF_004354835.1 | Chromosome | 4,268,950 | 877,439,799 | 28,914 | PRJNA514308 | SAMN10722690 |
| *Epinephelus lanceolatus* | giant grouper | ASM528154v1 | GCF_005281545.1 | Chromosome | 159,800 | 1,087,399,367 | 27,295 | PRJNA516312 | SAMN10786578 |
| *Epinephelus moara* | kelp grouper | YSFRI_EMoa_1.0 | GCF_006386435.1 | Chromosome | 76,174 | 1,030,460,988 | 26,752 | PRJNA543191 | SAMN11657372 |
| *Sander lucioperca* | pikeperch | SLUC_FBN_1.2 | GCF_008315115.2 | Chromosome | 6,663,498 | 901,221,791 | 33,533 | PRJNA561467 | SAMN12618724 |
| *Etheostoma spectabile* | orangethroat darter | UIUC_Espe_1.0 | GCF_008692095.1 | Chromosome | 26,023 | 854,790,067 | 38,154 | PRJNA556880 | SAMN12373147 |
| *Plectropomus leopardus* | leopard coralgrouper | YSFRI_Pleo_2.0 | GCF_008729295.1 | Chromosome | 897,070 | 895,688,574 | 29,473 | PRJNA545594 | SAMN11897347 |
| *Notolabrus celidotus* | New Zealand spotty | fNotCel1.pri | GCF_009762535.1 | Chromosome | 3,748,462 | 846,744,125 | 28,408 | PRJNA561956 | SAMN12623201 |
| *Cyclopterus lumpus* | lumpfish | fCycLum1.pri | GCF_009769545.1 | Chromosome | 4,950,682 | 572,885,595 | 26,630 | PRJNA562003 | SAMN12629502 |
| *Perca fluviatilis* | European perch | GENO_Pfluv_1.0 | GCF_010015445.1 | Chromosome | 4,197,643 | 951,345,774 | 32,186 | PRJNA549142 | SAMN12071746 |
| *Epinephelus fuscoguttatus* | brown-marbled grouper | E.fuscoguttatus.final_Chr_v1 | GCF_011397635.1 | Chromosome | 13,857,438 | 1,046,995,804 | 27,856 | PRJDB9224 | SAMD00201013 |
| *Etheostoma cragini* | Arkansas darter | CSU_Ecrag_1.0 | GCF_013103735.1 | Chromosome | 45,088 | 643,078,674 | 27,709 | PRJNA611833 | SAMN14351042 |
| *Sebastes umbrosus* | honeycomb rockfish | fSebUmb1.pri | GCF_015220745.1 | Chromosome | 11,445,908 | 800,904,020 | 30,334 | PRJNA562005 | SAMN12629503 |
| *Chelmon rostratus* | copperband butterflyfish | fCheRos1.pri | GCF_017976325.1 | Chromosome | 16,959,746 | 644,191,893 | 24,046 | PRJNA561969 | SAMN12623620 |
| *Cheilinus undulatus* | humphead wrasse | ASM1832078v1 | GCF_018320785.1 | Chromosome | 16,477,222 | 1,173,455,521 | 26,354 | PRJNA622923 | SAMN14532944 |
| *Siniperca chuatsi* | mandarin fish | ASM2008510v1 | GCF_020085105.1 | Chromosome | 20,478,450 | 718,804,017 | 27,696 | PRJNA738969 | SAMN19768590 |
| *Scatophagus argus* |  | fScaArg1.pri | GCF_020382885.2 | Chromosome | 17,189,422 | 570,785,501 | 26,110 | PRJNA749579 | SAMN12623616 |
| *Micropterus dolomieu* | smallmouth bass | ASM2129224v1 | GCF_021292245.1 | Chromosome | 46,775 | 829,570,727 | 31,328 | PRJNA777359 | SAMN22851699 |
| *Cottoperca gobio* |  | fCotGob3.1 | GCF_900634415.1 | Chromosome | 6,330,900 | 609,391,784 | 25595 | PRJEB30248 | SAMEA104242975 |
| *Sparus aurata* | gilthead seabream | fSpaAur1.1 | GCF_900880675.1 | Chromosome | 2,862,625 | 833,578,411 | 32,008 | PRJEB31901 | SAMEA104384835 |
| *Takifugu rubripes* | torafugu | fTakRub1.2 | GCF_901000725.2 | Chromosome | 3,136,617 | 384,110,215 | 27,412 | PRJEB31988 | SAMEA104384834 |
| *Pseudochaenichthys georgianus* | South Georgia icefish | fPseGeo1.1 | GCF_902827115.1 | Chromosome | 661,283 | 1,026,101,545 | 29,469 | PRJEB37648 | SAMEA104132832 |
| *Acanthopagrus latus* | yellowfin seabream | fAcaLat1.1 | GCF_904848185.1 | Chromosome | 14,880,455 | 685,127,588 | 30,246 | PRJEB40700 | SAMN16236878 |

**Table S7**: Statistics of genes predicted in the *L. argentimaculatus* genome.

| **Attributes** | **Value** |
| --- | --- |
| Total sequence length | 1,030,266,718 |
| Number of genes | 27,172 |
| Number of mRNAs | 27,172 |
| Number of exons | 211,939 |
| Number of introns | 184,767 |
| Number of CDS | 27,172 |
| CDS: complete | 21,629 |
| CDS: start, no stop | 2,207 |
| CDS: stop, no start | 2,431 |
| CDS: no stop, no start | 905 |
| Total gene length | 386,711,962 |
| Total mRNA length | 386,711,962 |
| Total exon length | 34,226,502 |
| Total intron length | 352,854,994 |
| Total CDS length | 34,226,502 |
| Shortest gene | 150 |
| Shortest mRNA | 150 |
| Longest gene | 99,395 |
| Longest mRNA | 99,395 |
| Longest exon | 8,565 |
| Longest intron | 96,463 |
| Longest CDS | 31,071 |
| mean gene length | 14,232 |
| mean mRNA length | 14,232 |
| mean exon length | 161 |
| mean intron length | 1,910 |
| mean CDS length | 1,260 |
| % of genome covered by genes | 37.5 |
| % of genome covered by CDS | 3.3 |
| mean mRNAs per gene | 1 |
| mean exons per mRNA | 8 |
| mean introns per mRNA | 7 |

**Table S8:** Genomes of available Lutjanus species

| Species | *L. argentimaculatus* | *L. campechanus* | *L. campechanus* | *L. erypthropterus* |
| --- | --- | --- | --- | --- |
| Author | Shekhar et al. | Norrell et al. | Portnoy et al. | Lai et al. |
| Genome length | 1,030,266,718 bp | 770,602,665 bp | 1,202,279,098 bp | 969,885,659 bp |
| No of scaffolds | 400 | 67,254 | 140,690 | 10,853 |
| Scaffold N50 | 33,807,785 bp | 16,803 bp | 20,750 bp | 40,645,435 bp |
| No. of contigs | 382 | 76,321 | 205,466 | 11,914 |
| Contig N50 | 12,244,877 bp | 14,414 bp | 15,631 bp | 2,781,023 bp |
