# Supplementary material for: Genome assembly, Full-length transcriptome, and isoform diversity of Red Snapper, Lutjanus argentimaculatus
Source: Sci Data. 2024 Jul 18;11:796. doi: 10.1038/s41597-024-03633-1 (PMC11258364; doi:10.1038/s41597-024-03633-1)
Supplement: Supplementary file 3 — Supplementary File [file 41597_2024_3633_MOESM3_ESM.pdf]

SQANTI3 report

Unique Genes: 18108

Unique Isoforms: 56515

Transcript Classification

Category	Isoforms, count
FSM	6008
ISM	2121
NIC	1475
NNC	24834
Genic Genomic	960
Antisense	231
Fusion	18347
Intergenic	2539
Genic Intron	0

Gene Classification

Category	Genes, count
Annotated Genes	15402
Novel Genes	2706

Splice Junction Classification

Category	SJs, count	Percent
Known canonical	129928	71.62
Known Non−canonical	5	0.00
Novel canonical	37601	20.73
Novel Non−canonical	13886	7.65

Gene Characterization

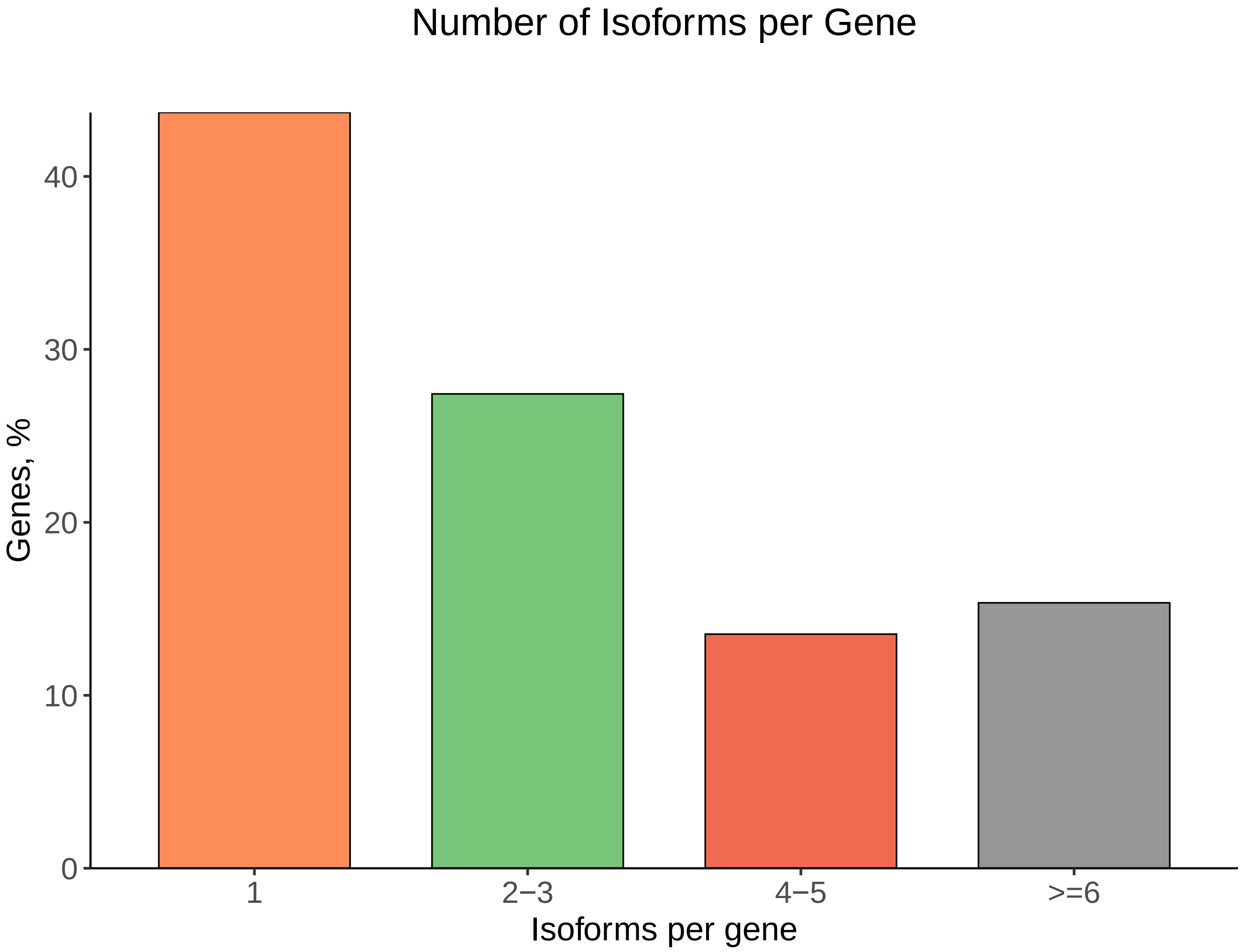
Number of Isoforms per Gene
Genes, %
40
30
20
10
0
1
2−3
4−5
>=6
Isoforms per gene

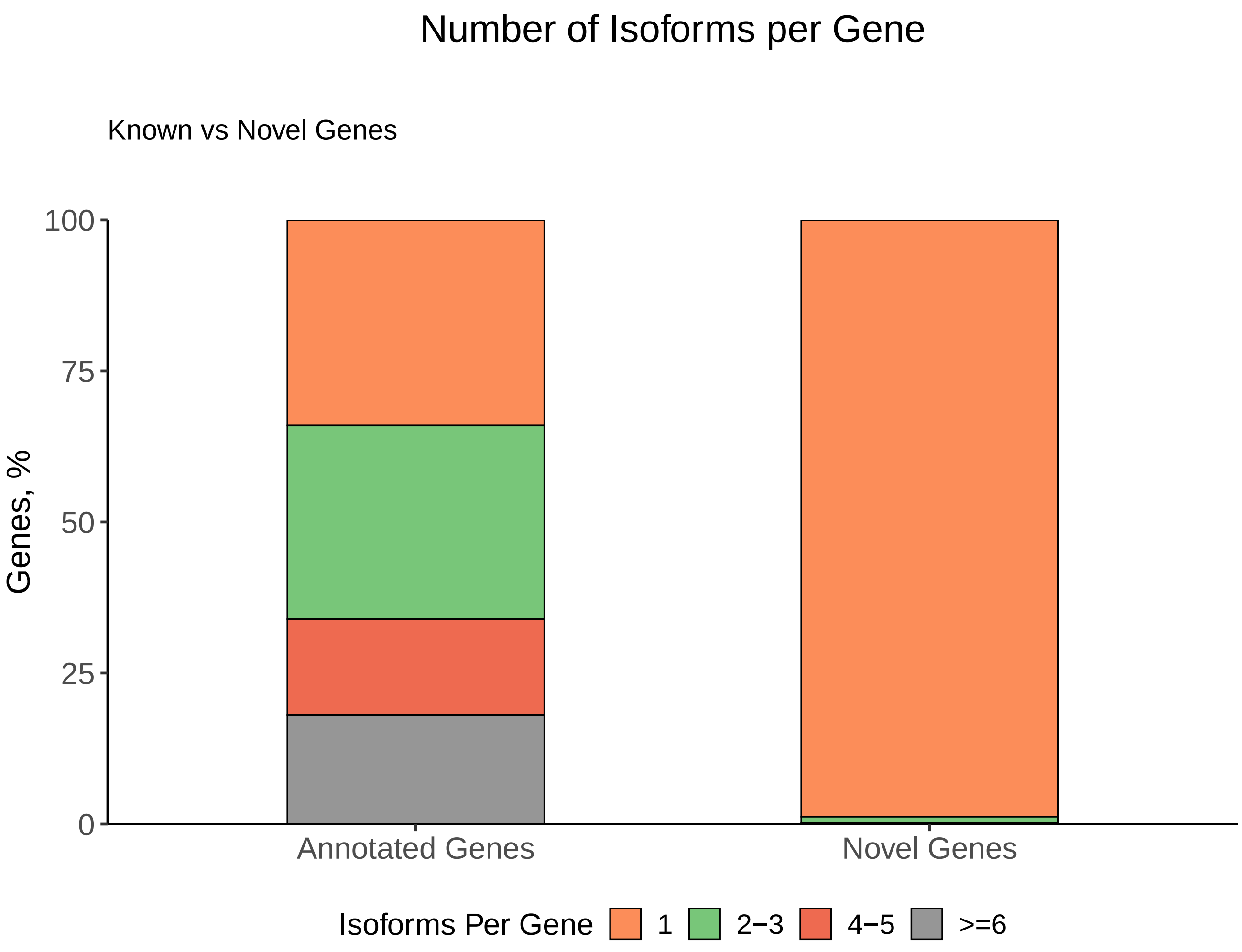

Number of Isoforms per Gene
Known vs Novel Genes
Genes, %
100
75
50
25
0
Annotated Genes
Novel Genes
Isoforms Per Gene
1
2−3
4−5
>=6

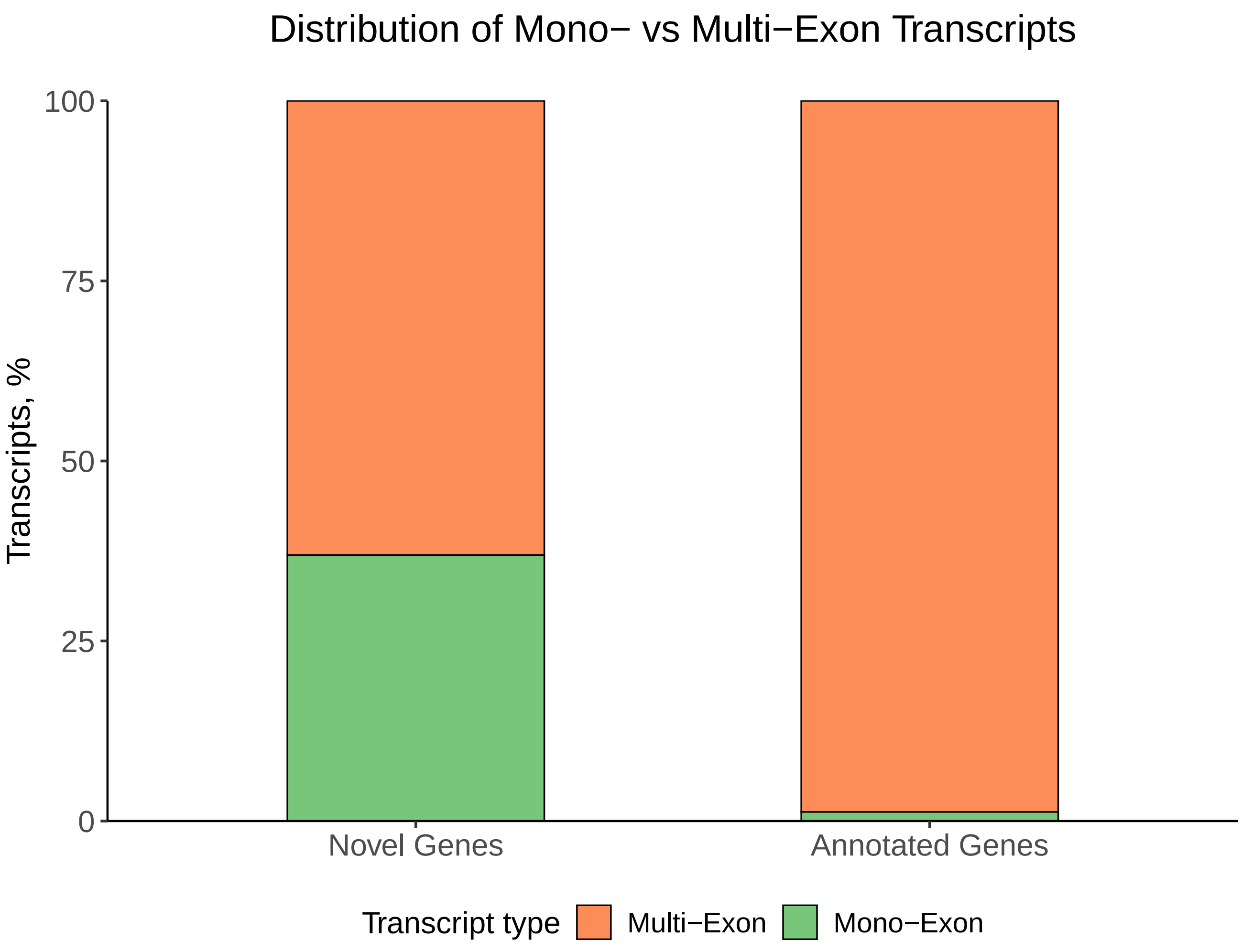
Distribution of Mono− vs Multi−Exon Transcripts
Transcripts, %
100
75
50
25
0
Novel Genes
Annotated Genes
Transcript type
Multi−Exon
Mono−Exon

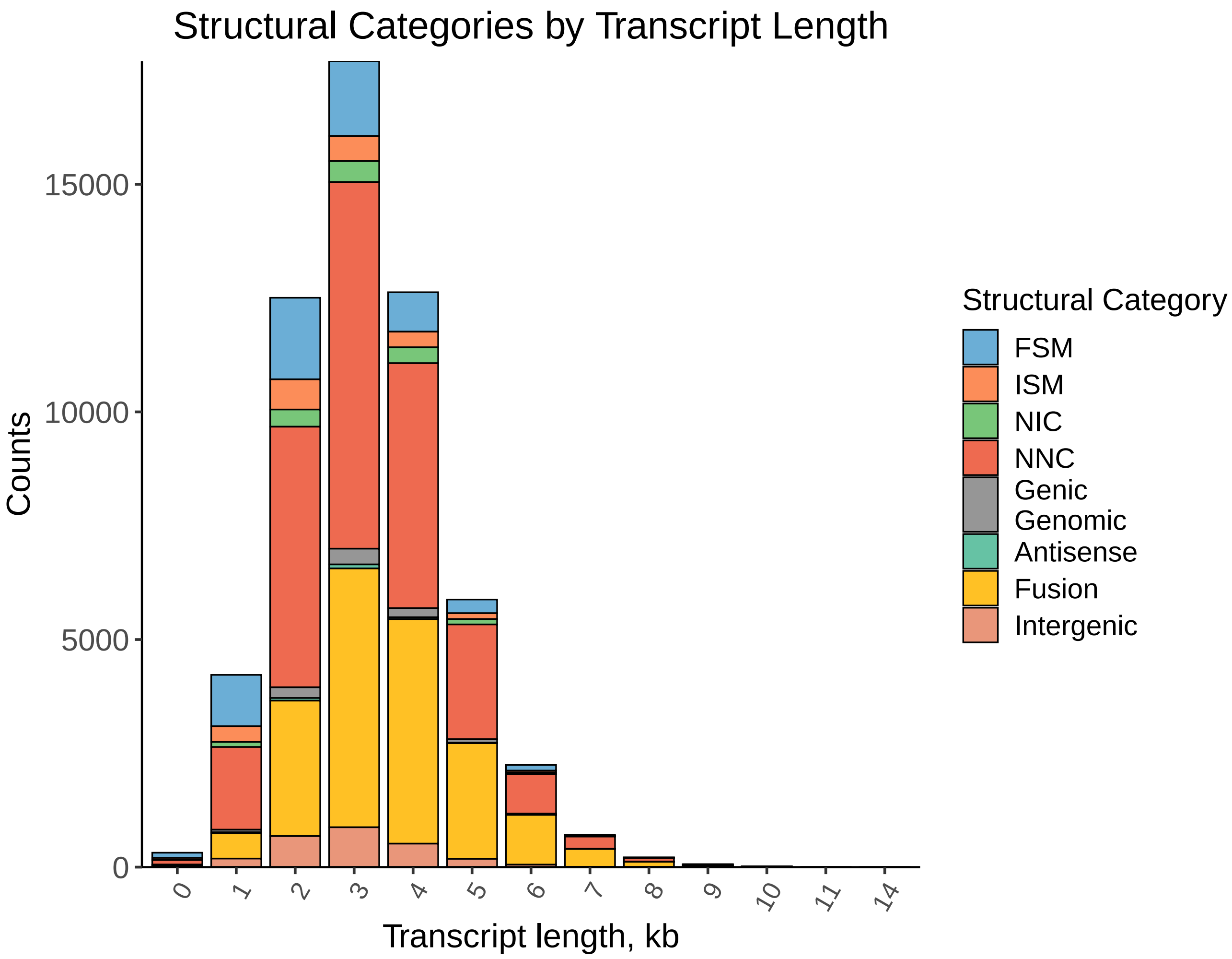
Structural Categories by Transcript Length
Counts
15000
10000
5000
0
0
1
2
3
4
5
6
7
8
9
10
11
14
Transcript length, kb
Structural Category
FSM
ISM
NIC
NNC
Genic Genomic
Antisense
Fusion
Intergenic

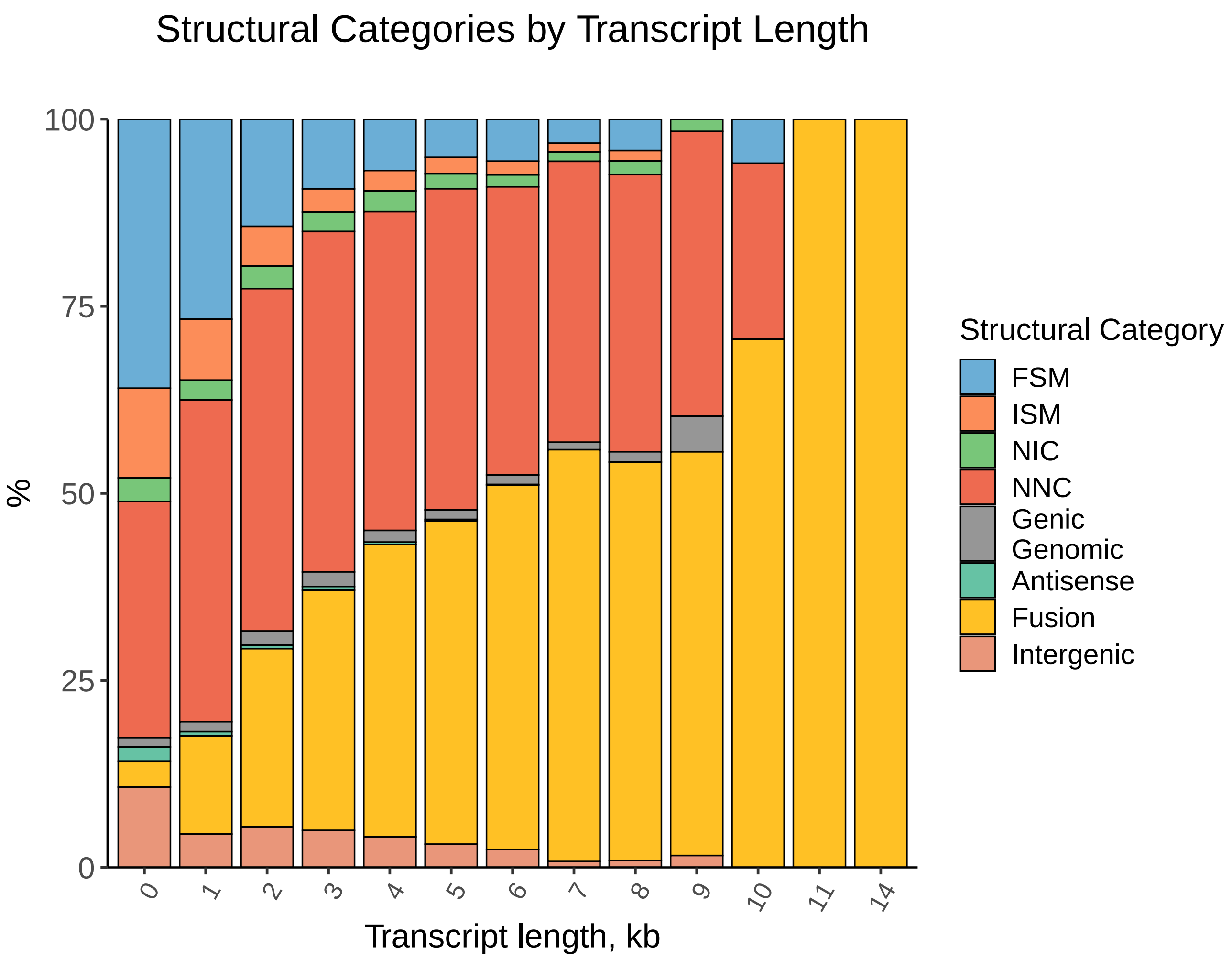

Structural Categories by Transcript Length
%
100
75
50
25
0
0
1
2
3
4
5
6
7
8
9
10
11
14
Transcript length, kb
Structural Category
FSM
ISM
NIC
NNC
Genic
Genomic
Antisense
Fusion
Intergenic

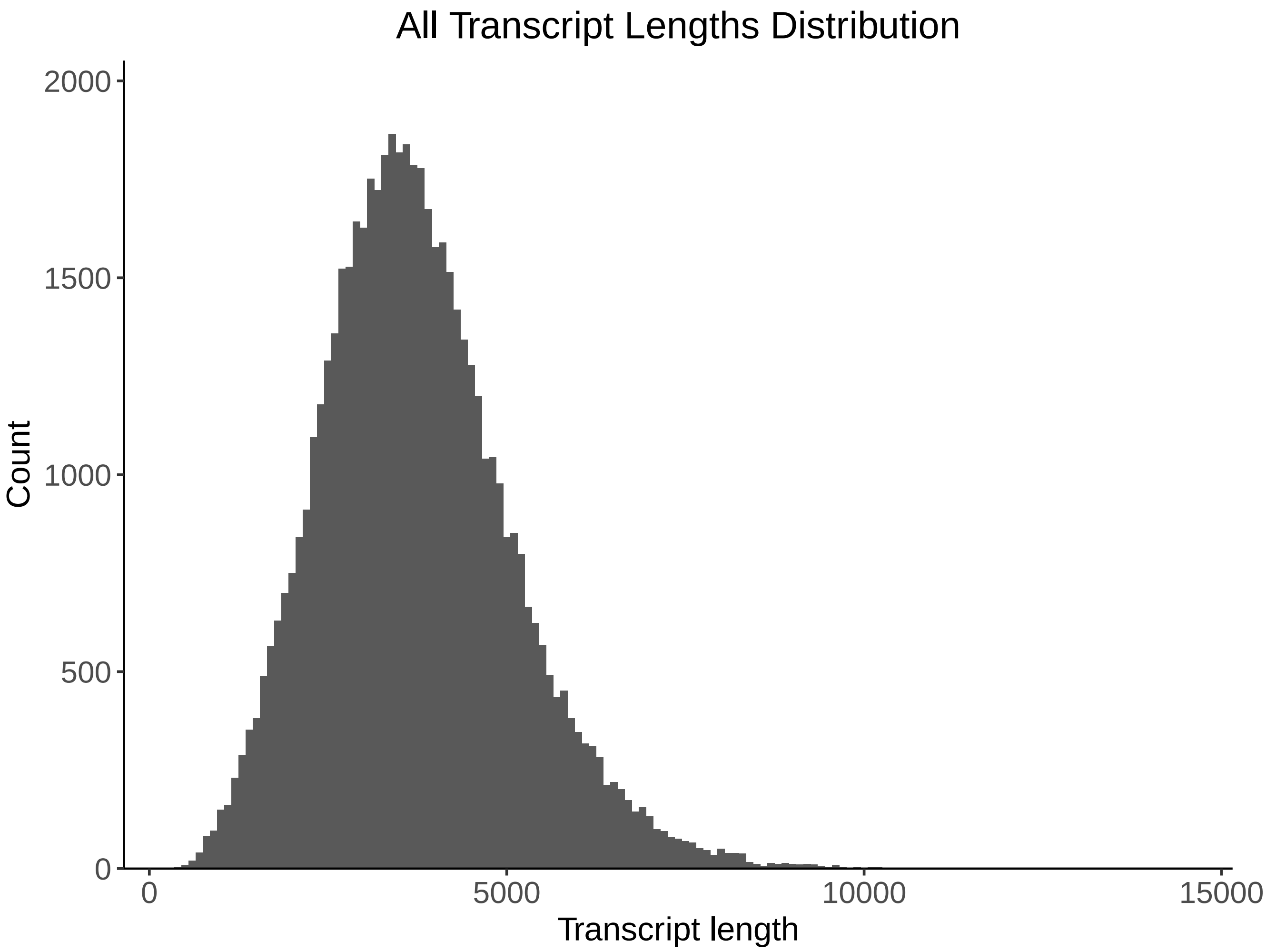
All Transcript Lengths Distribution
Count
2000
1500
1000
500
0
0
5000
10000
15000
Transcript length

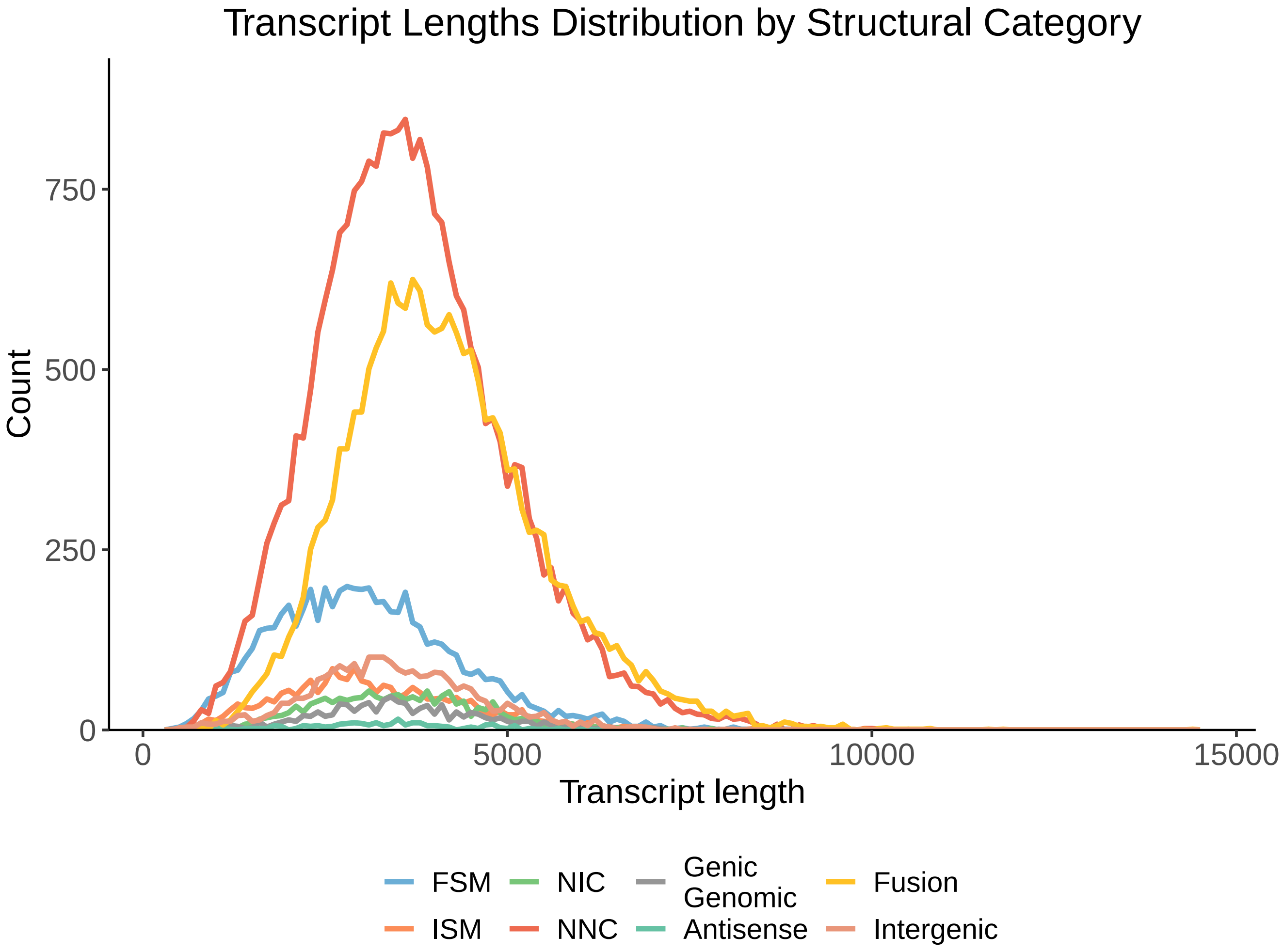

Transcript Lengths Distribution by Structural Category
Count
750
500
250
0
0
5000
10000
15000
Transcript length
FSM
ISM
NIC
NNC
Genic
Genomic
Antisense
Fusion
Intergenic

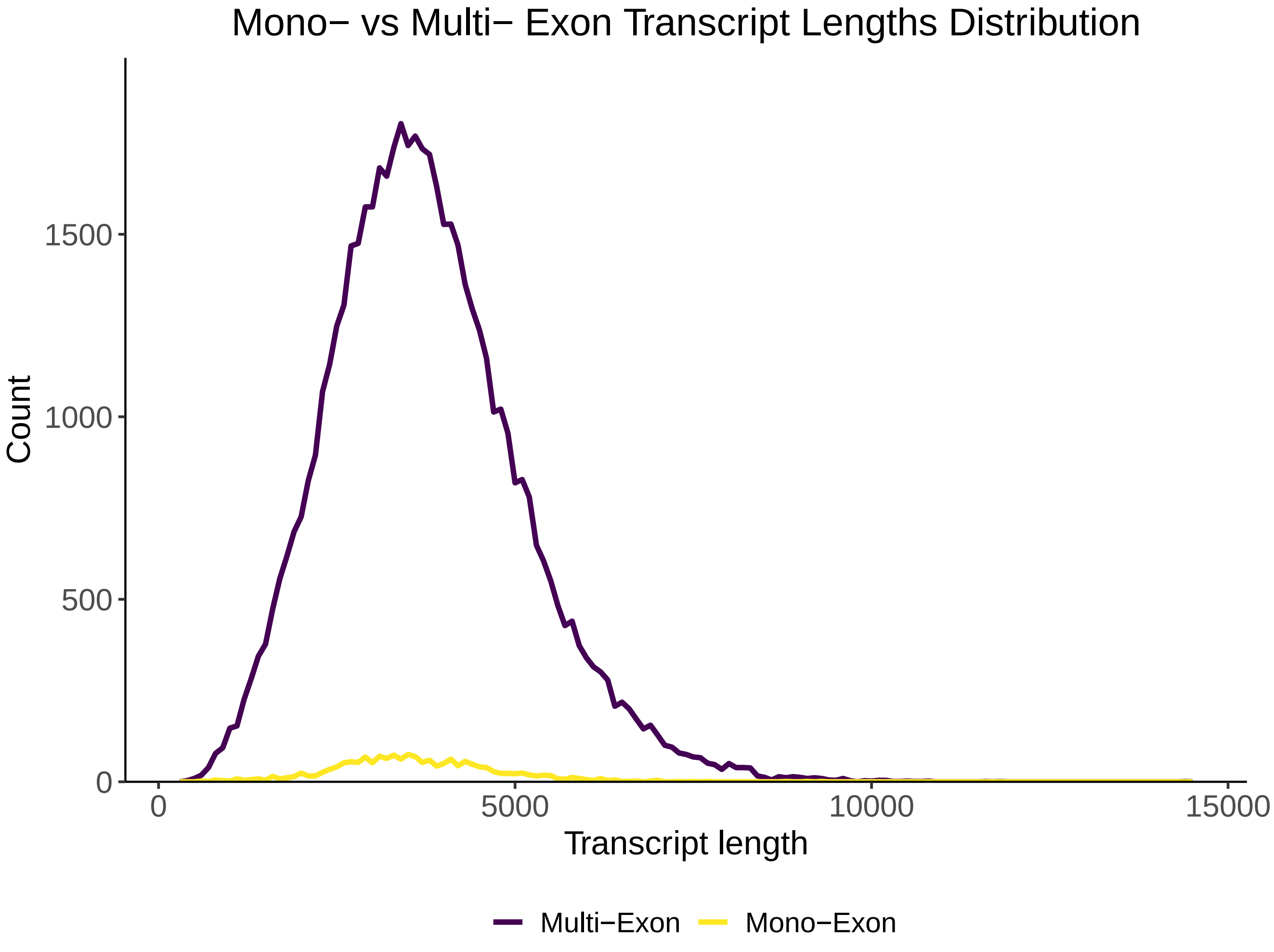
Mono− vs Multi− Exon Transcript Lengths Distribution
Count
1500
1000
500
0
0
5000
10000
15000
Transcript length
Multi−Exon
Mono−Exon

Structural Isoform Characterization

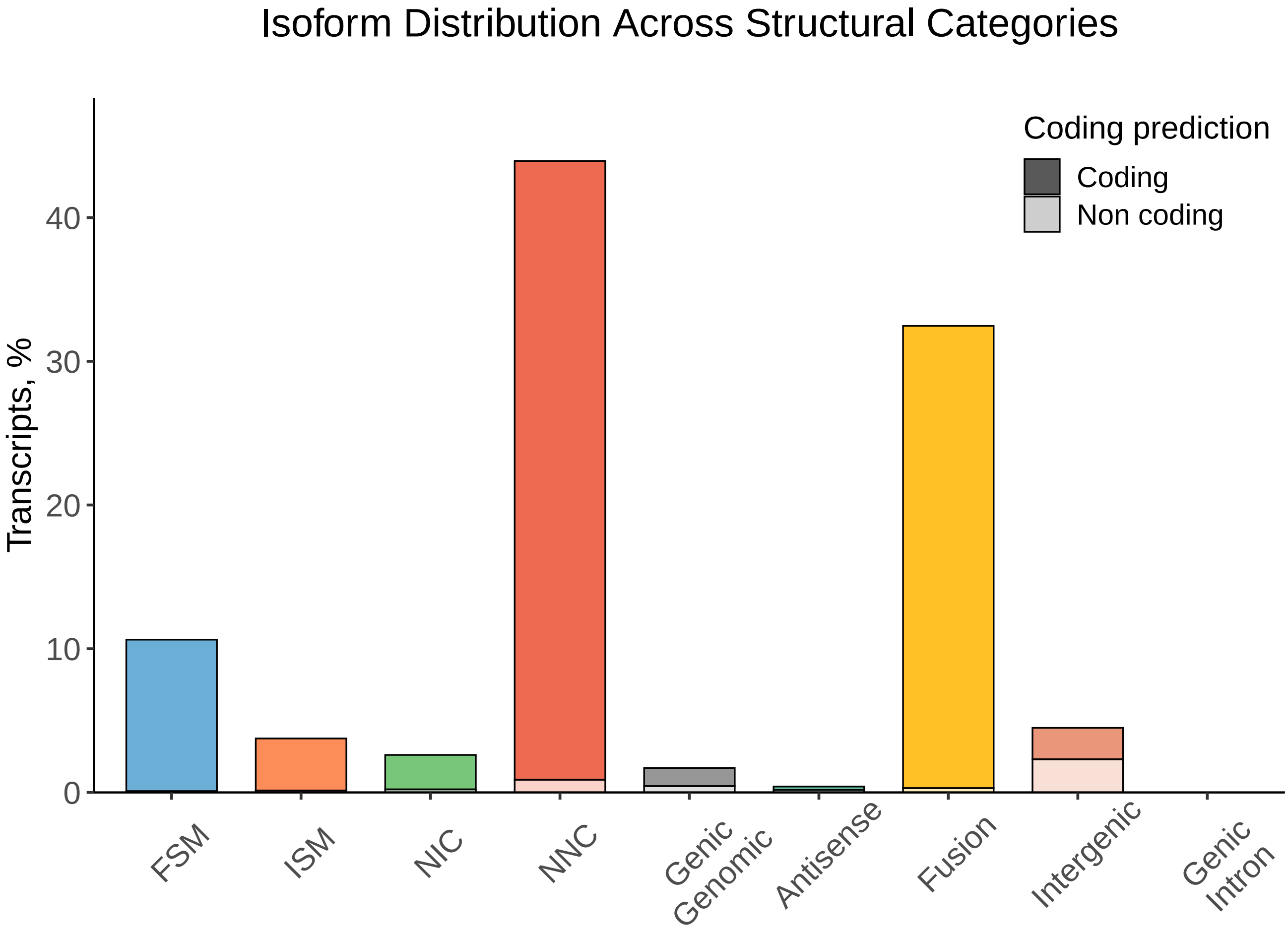
Isoform Distribution Across Structural Categories
Coding prediction
Coding
Non coding
Transcripts, %
0
10
20
30
40
FSM
ISM
NIC
NNC
Genic
Genomic
Antisense
Fusion
Intergenic
Genic
Intron

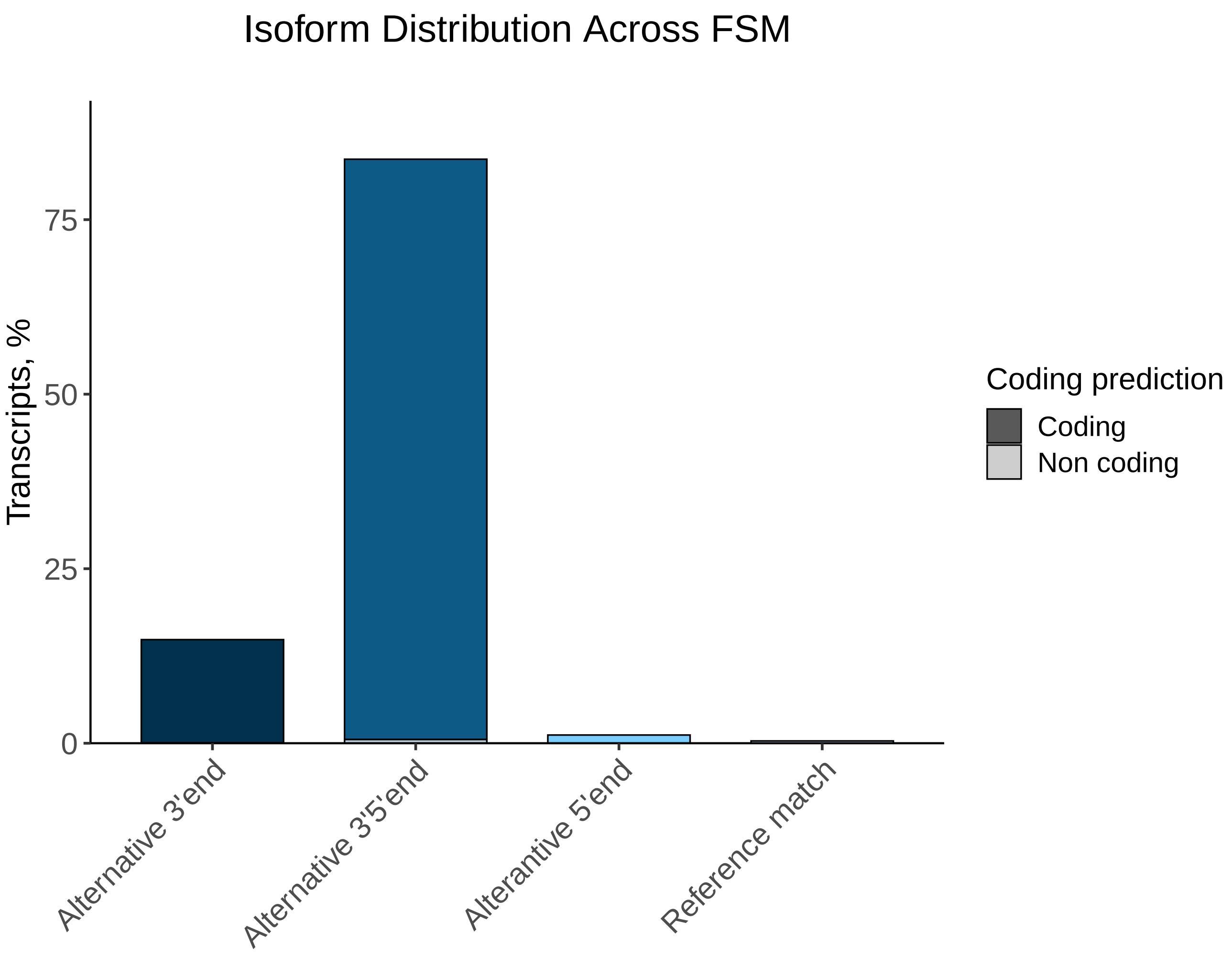
Isoform Distribution Across FSM
Transcripts, %
0
25
50
75
Alternative 3'end
Alternative 3'5'end
Alterantive 5'end
Reference match
Coding prediction
Coding
Non coding

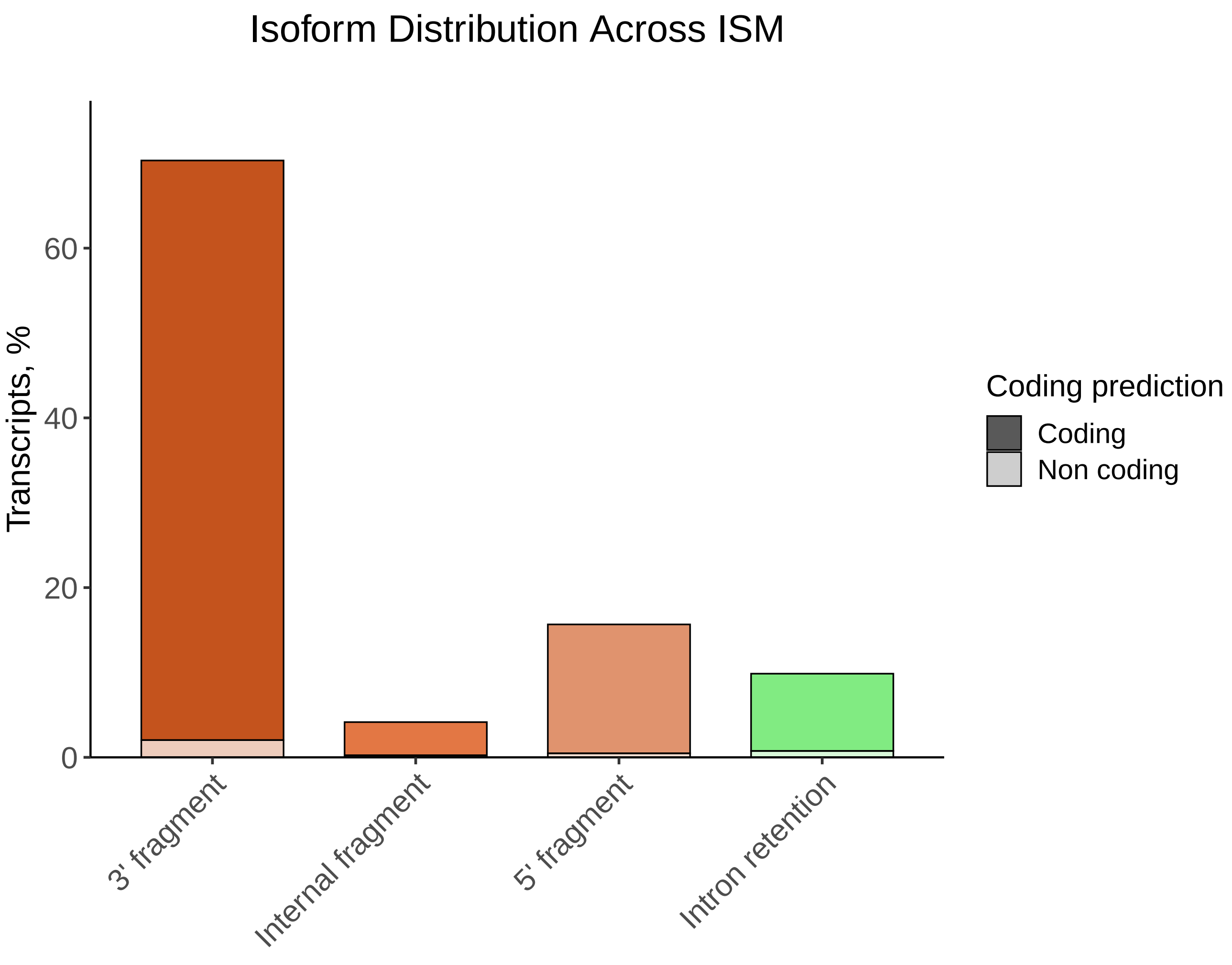

Isoform Distribution Across ISM
Transcripts, %
60
40
20
0
3' fragment
Internal fragment
5' fragment
Intron retention
Coding prediction
Coding
Non coding

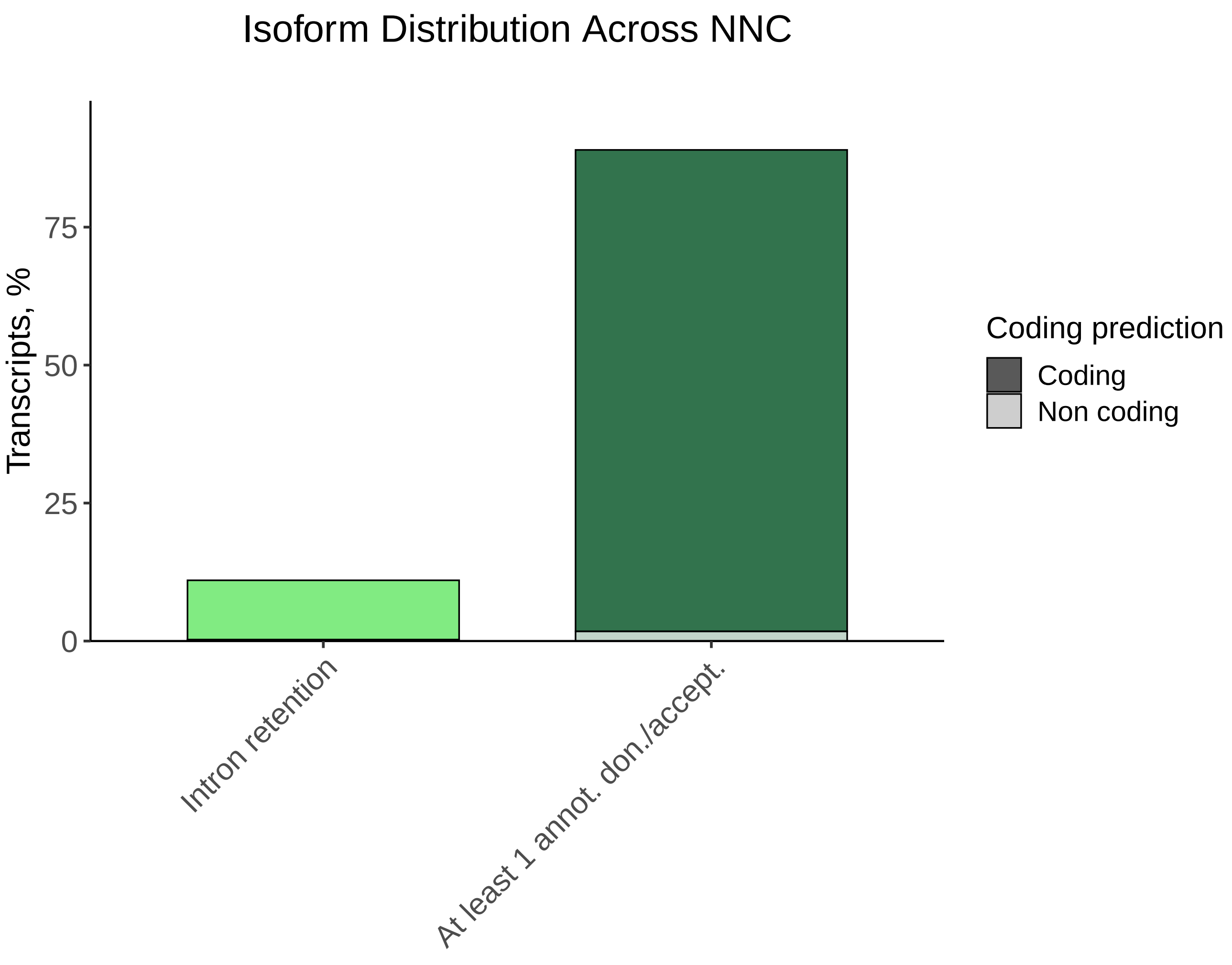

Isoform Distribution Across NNC
Transcripts, %
0
25
50
75
Intron retention
At least 1 annot. don./accept.
Coding prediction
Coding
Non coding

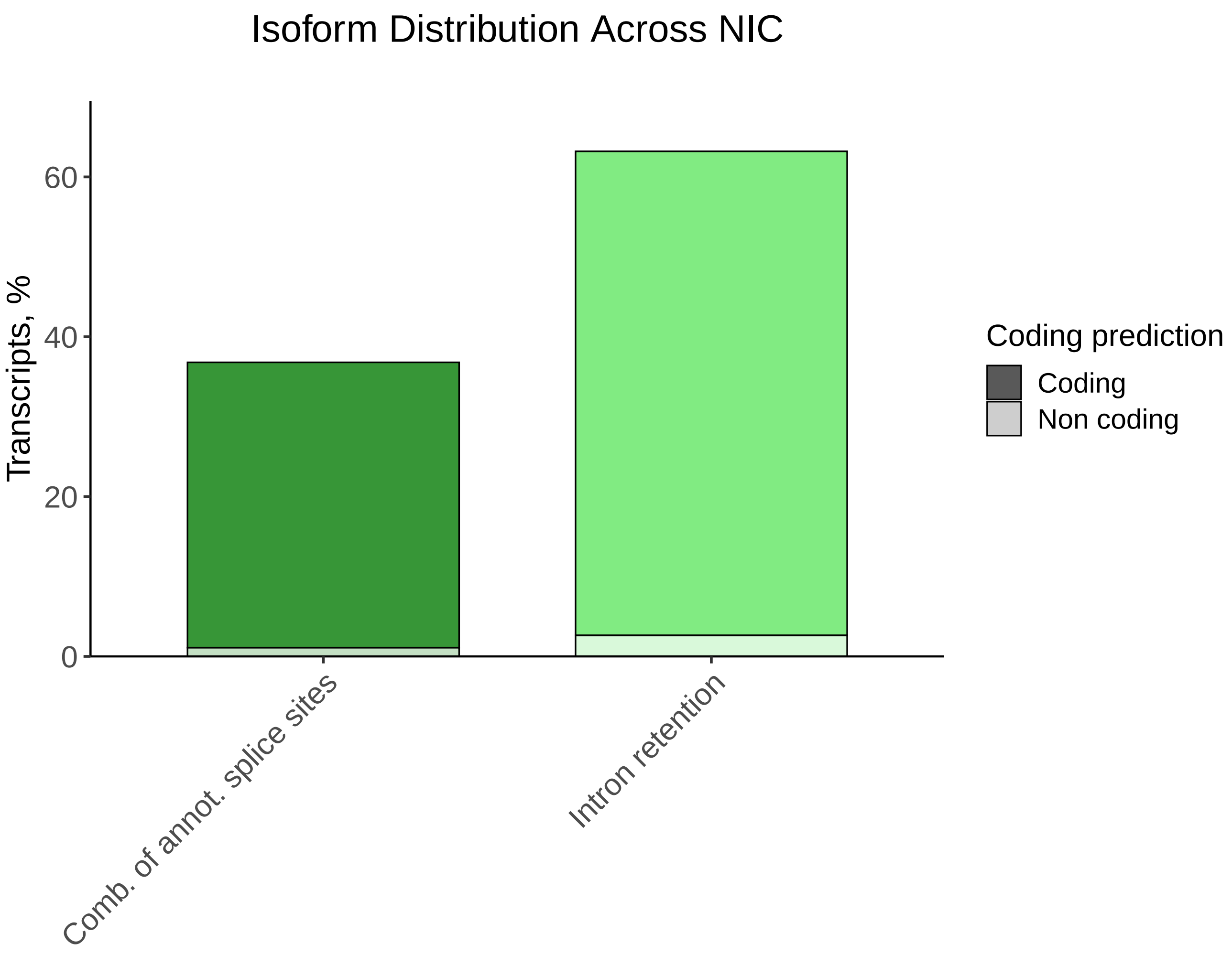

Isoform Distribution Across NIC
Transcripts, %
0
20
40
60
Comb. of annot. splice sites
Intron retention
Coding prediction
Coding
Non coding

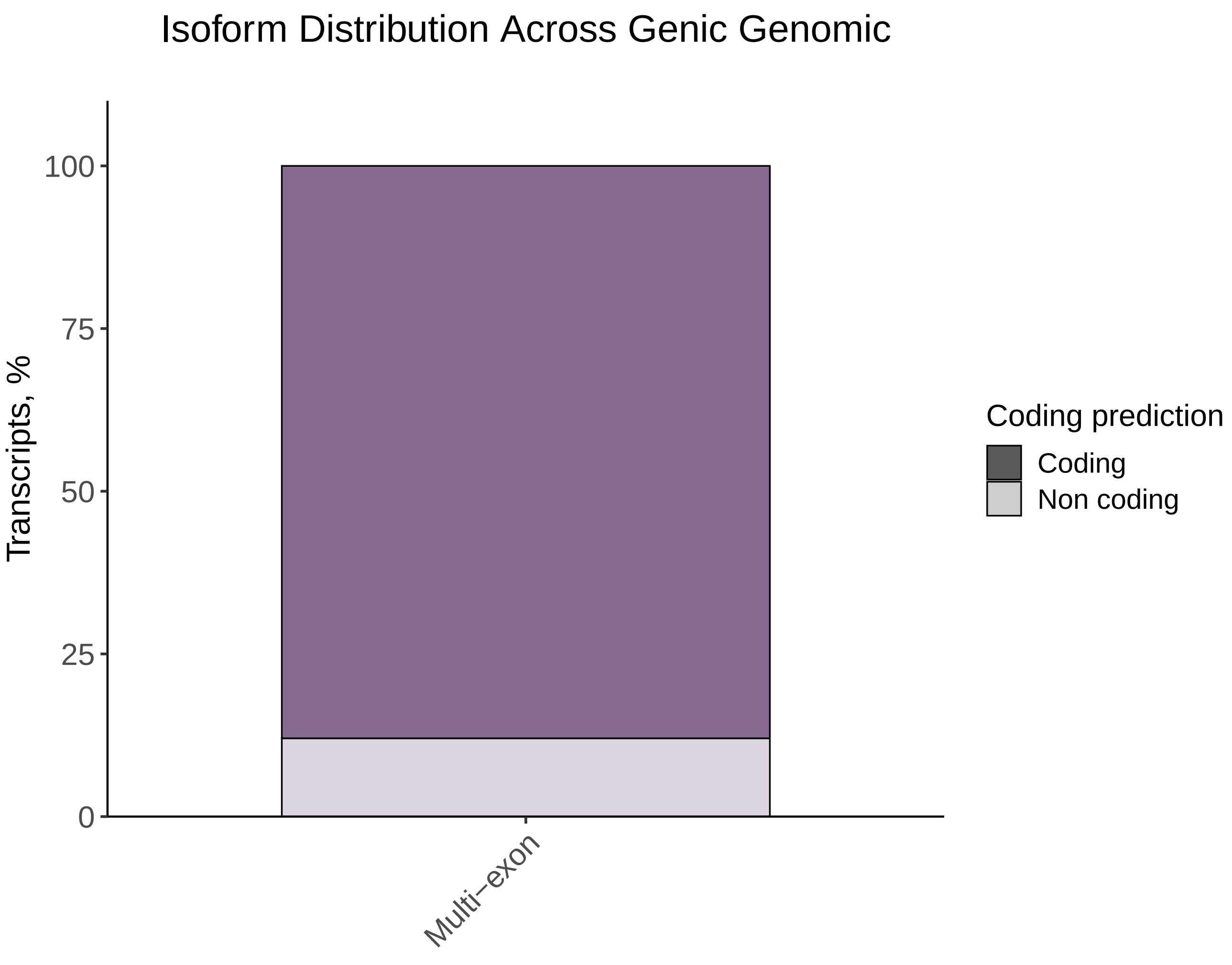

Isoform Distribution Across Genic Genomic
Transcripts, %
100
75
50
25
0
Multi−exon
Coding prediction
Coding
Non coding

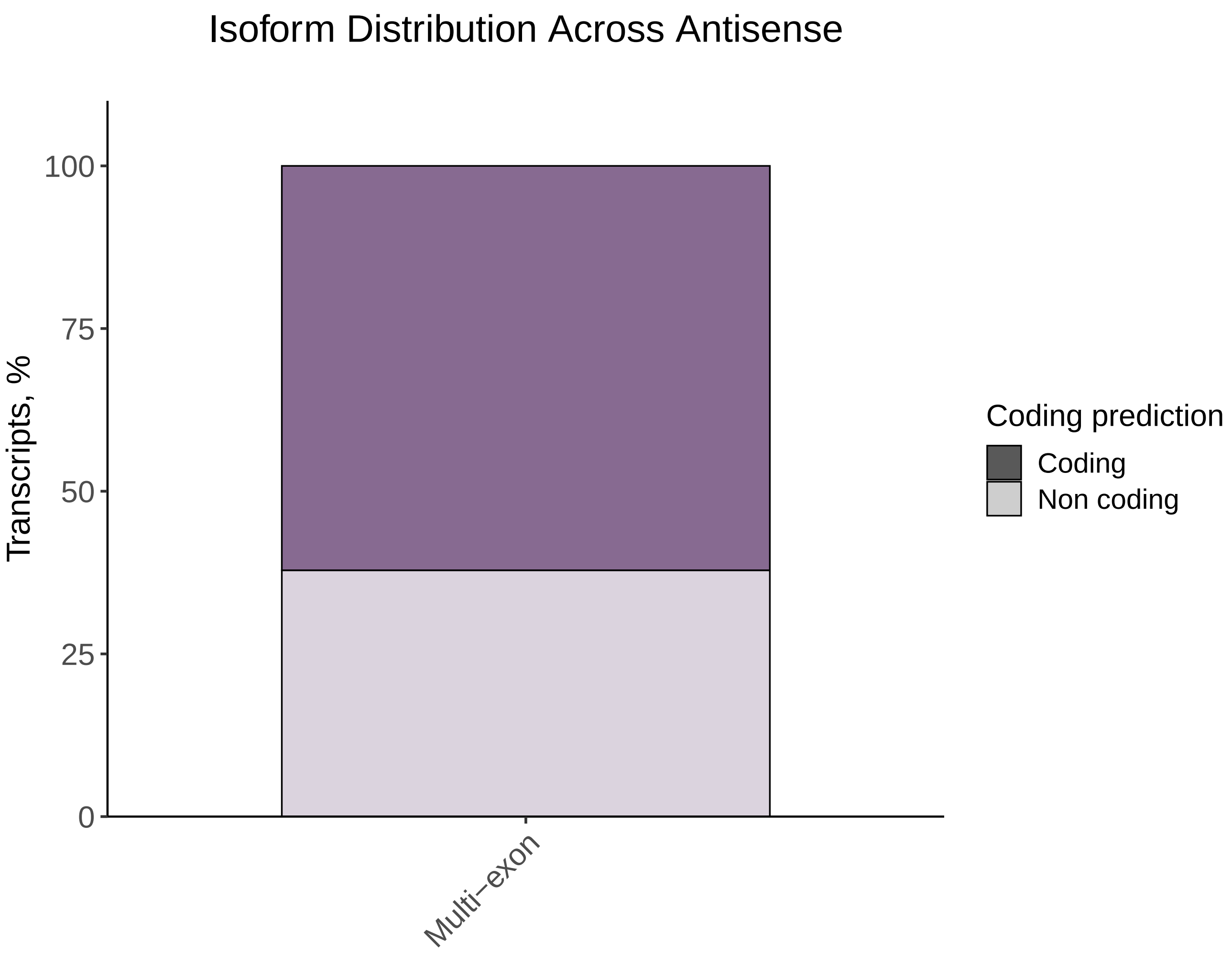
Isoform Distribution Across Antisense
Transcripts, %
100
75
50
25
0
Multi−exon
Coding prediction
Coding
Non coding

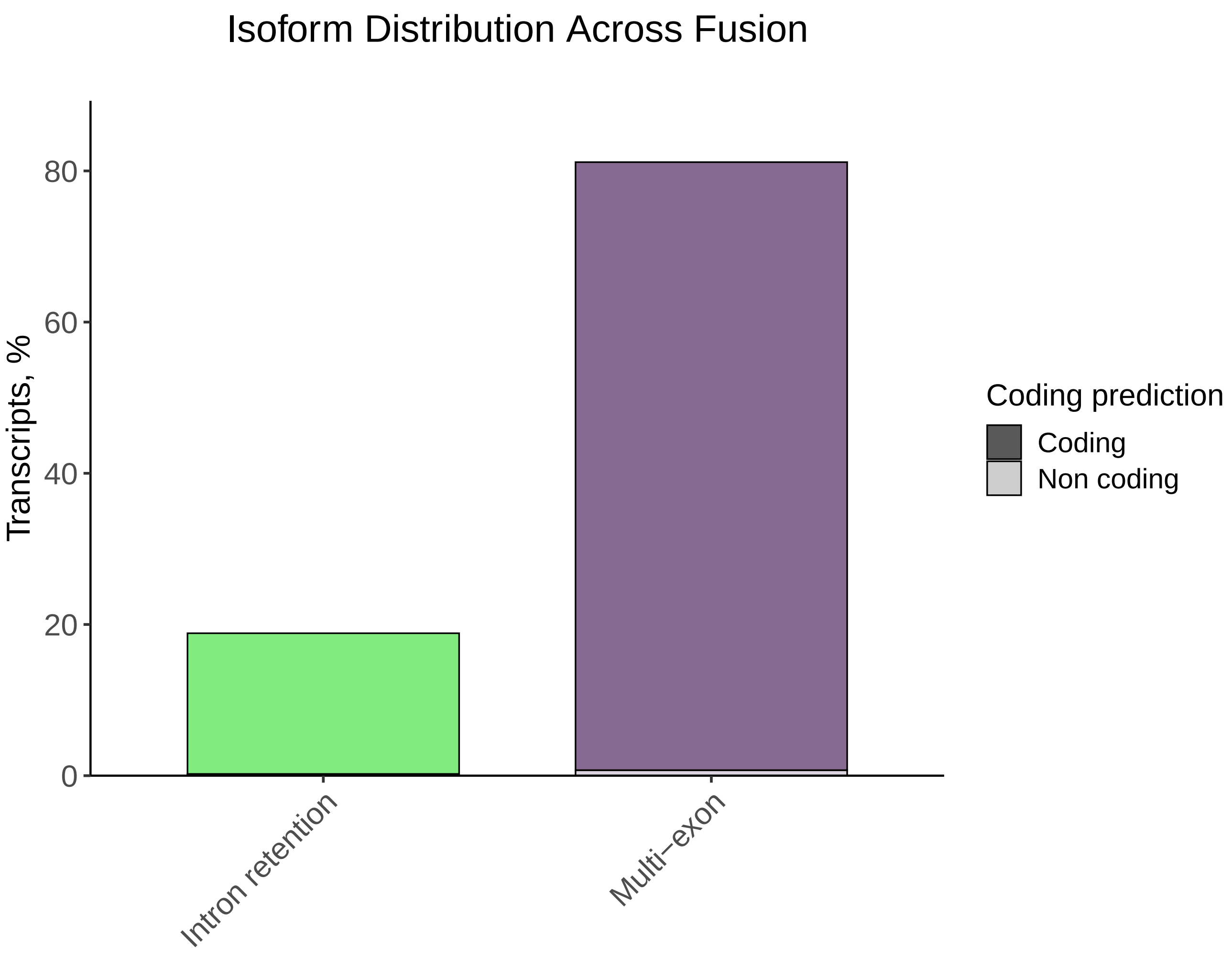
Isoform Distribution Across Fusion
Transcripts, %
0
20
40
60
80
Intron retention
Multi−exon
Coding prediction
Coding
Non coding

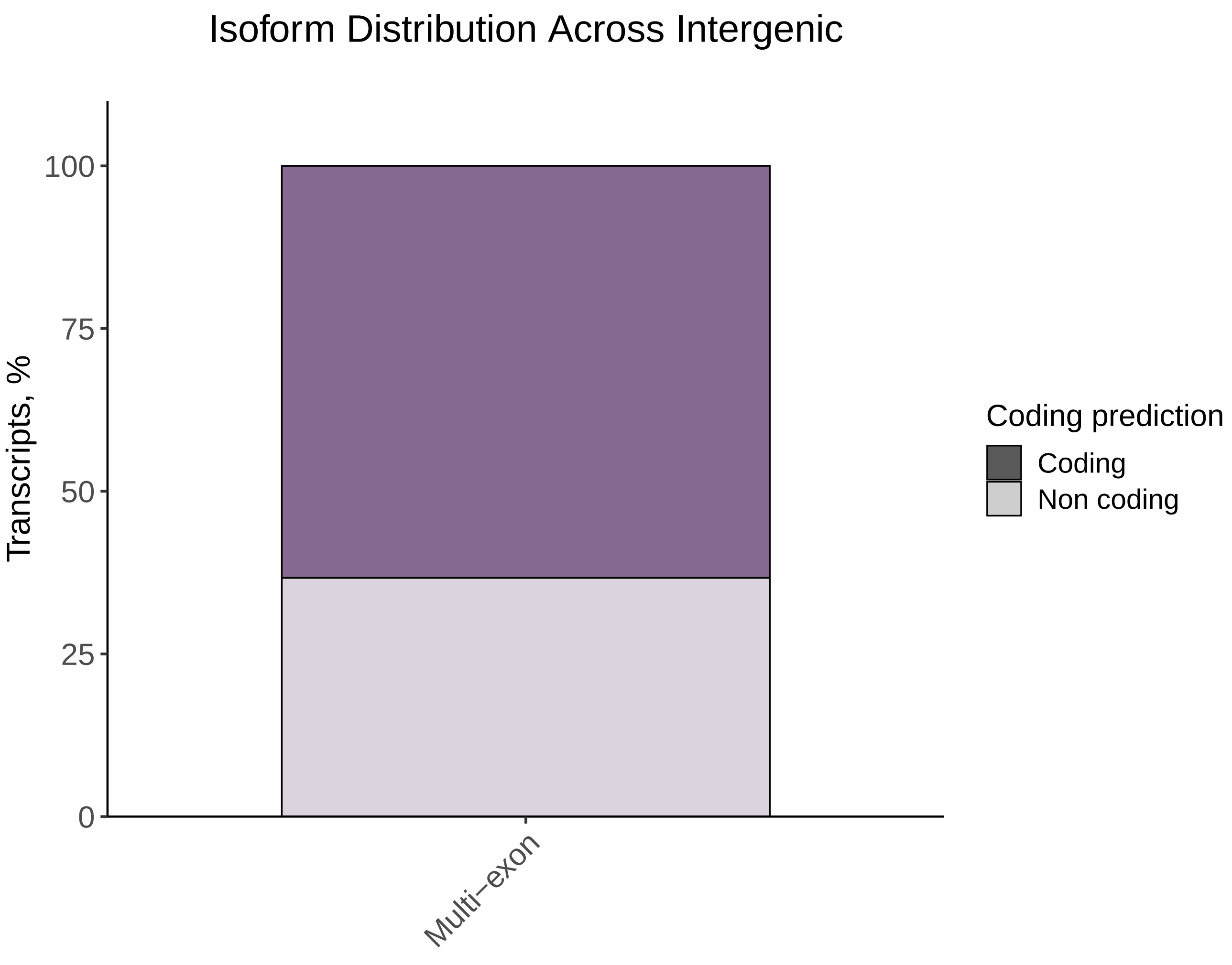

Isoform Distribution Across Intergenic
Transcripts, %
100
75
50
25
0
Multi−exon
Coding prediction
Coding
Non coding

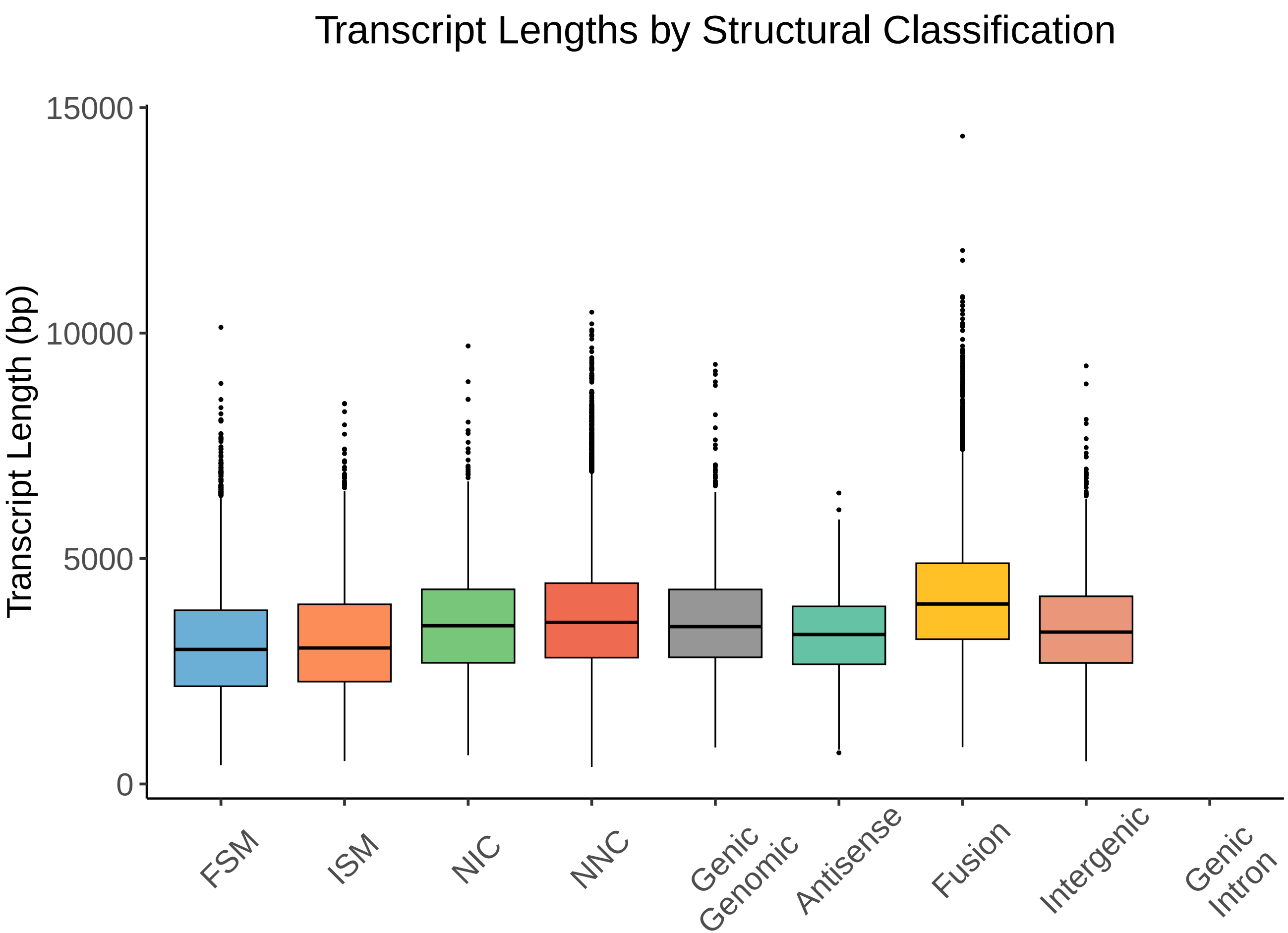

Transcript Lengths by Structural Classification
Transcript Length (bp)
15000
10000
5000
0
FSM
ISM
NIC
NNC
Genic Genomic
Antisense
Fusion
Intergenic
Genic Intron

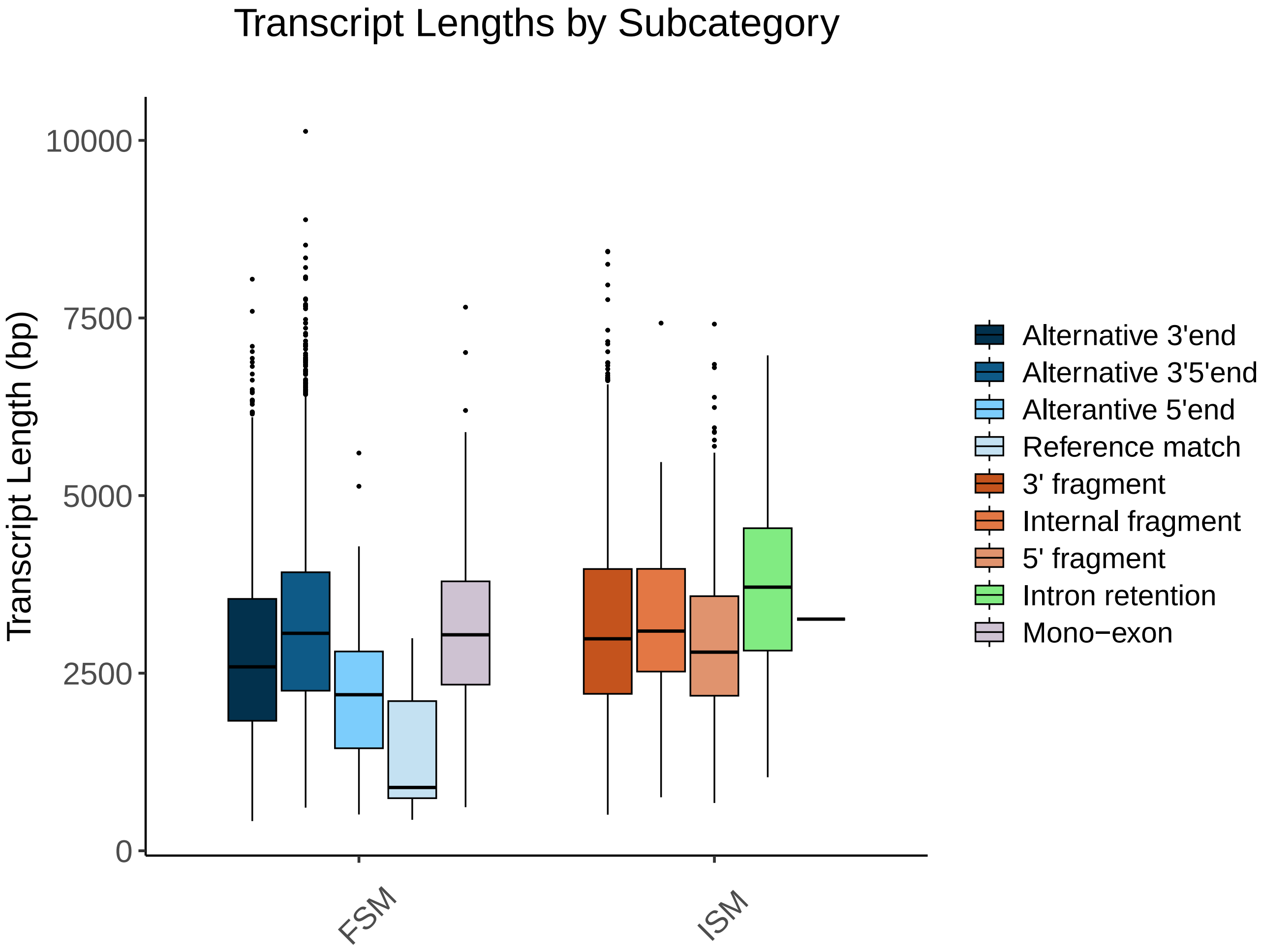

Transcript Lengths by Subcategory
Transcript Length (bp)
10000
7500
5000
2500
0
FSM
ISM
Alternative 3'end
Alternative 3'5'end
Alterantive 5'end
Reference match
3' fragment
Internal fragment
5' fragment
Intron retention
Mono−exon

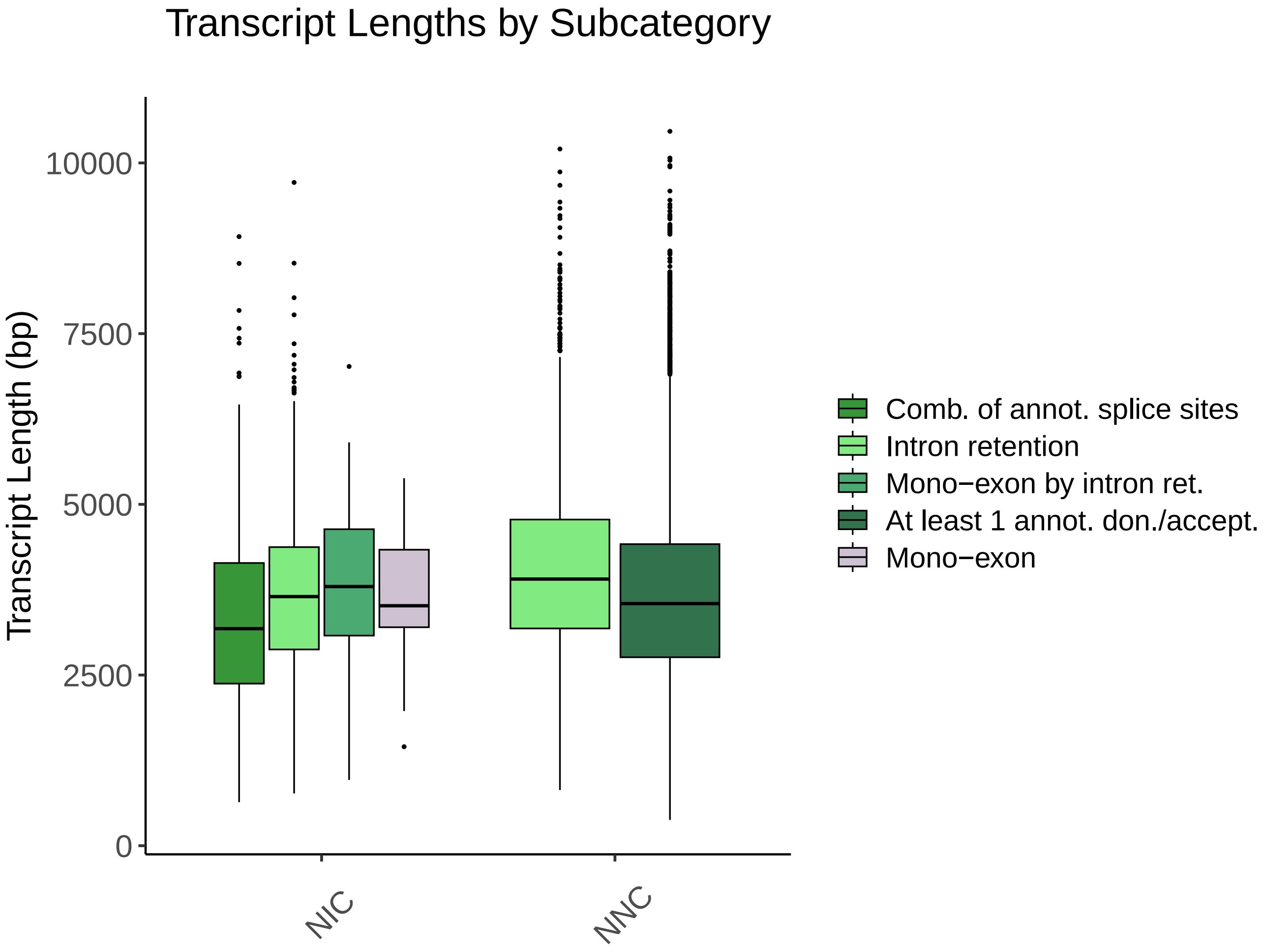
Transcript Lengths by Subcategory
Transcript Length (bp)
10000
7500
5000
2500
0
NIC
NNC
Comb. of annot. splice sites
Intron retention
Mono−exon by intron ret.
At least 1 annot. don./accept.
Mono−exon

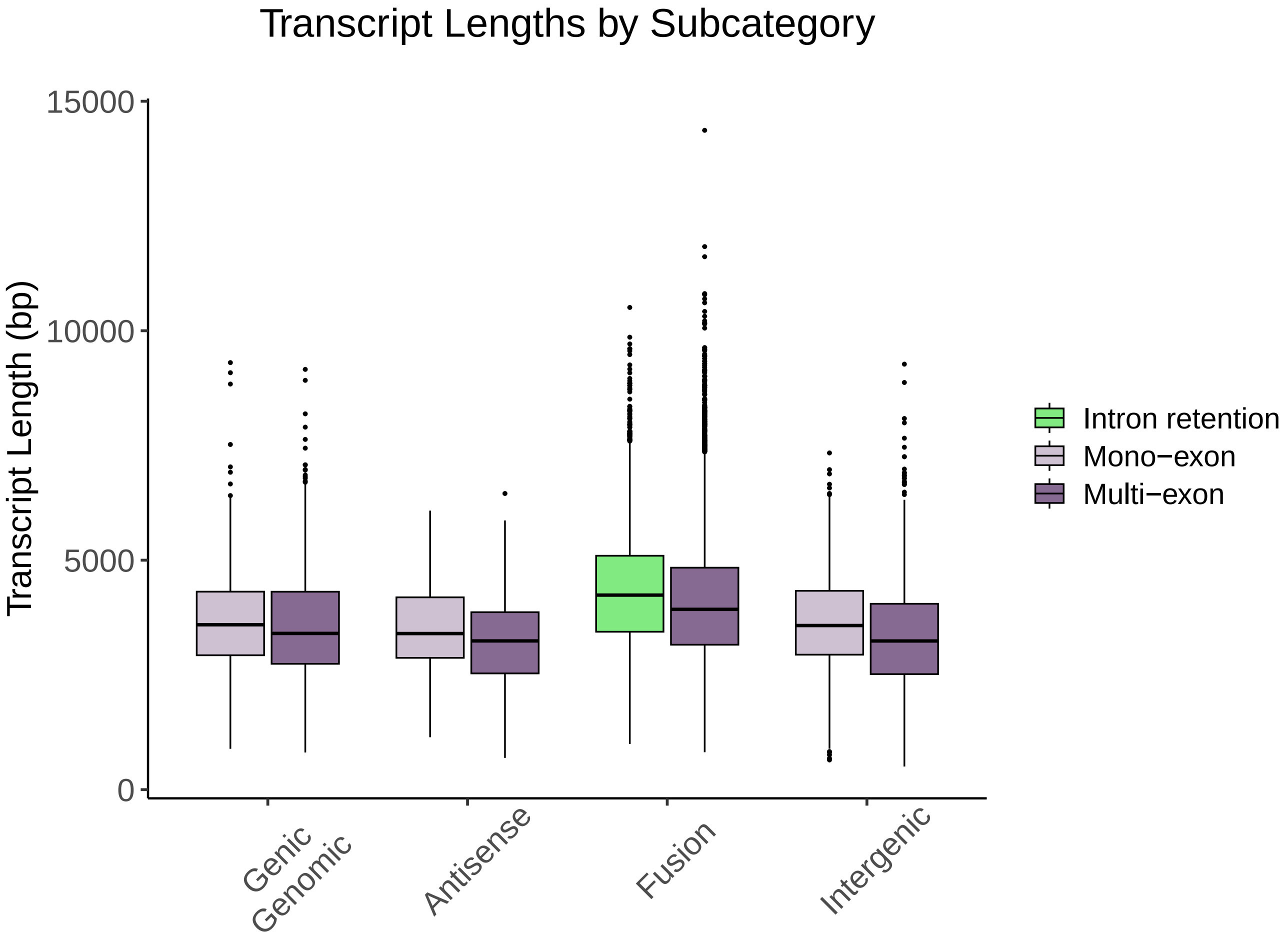
Transcript Lengths by Subcategory
Transcript Length (bp)
15000
10000
5000
0
Genic
Genomic
Antisense
Fusion
Intergenic
Intron retention
Mono−exon
Multi−exon

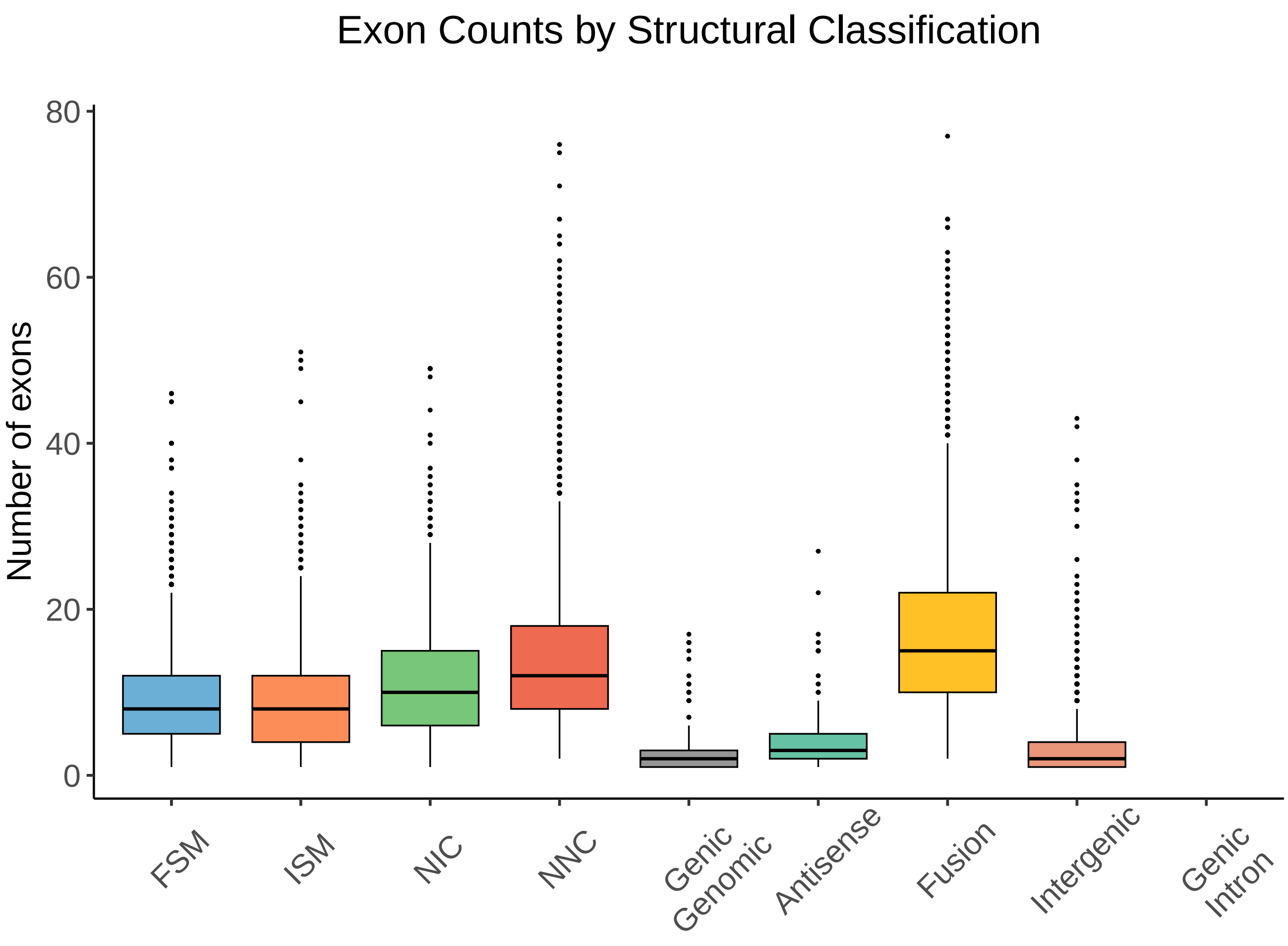

Exon Counts by Structural Classification
Number of exons
0
20
40
60
80
FSM
ISM
NIC
NNC
Genic Genomic
Antisense
Fusion
Intergenic
Genic Intron

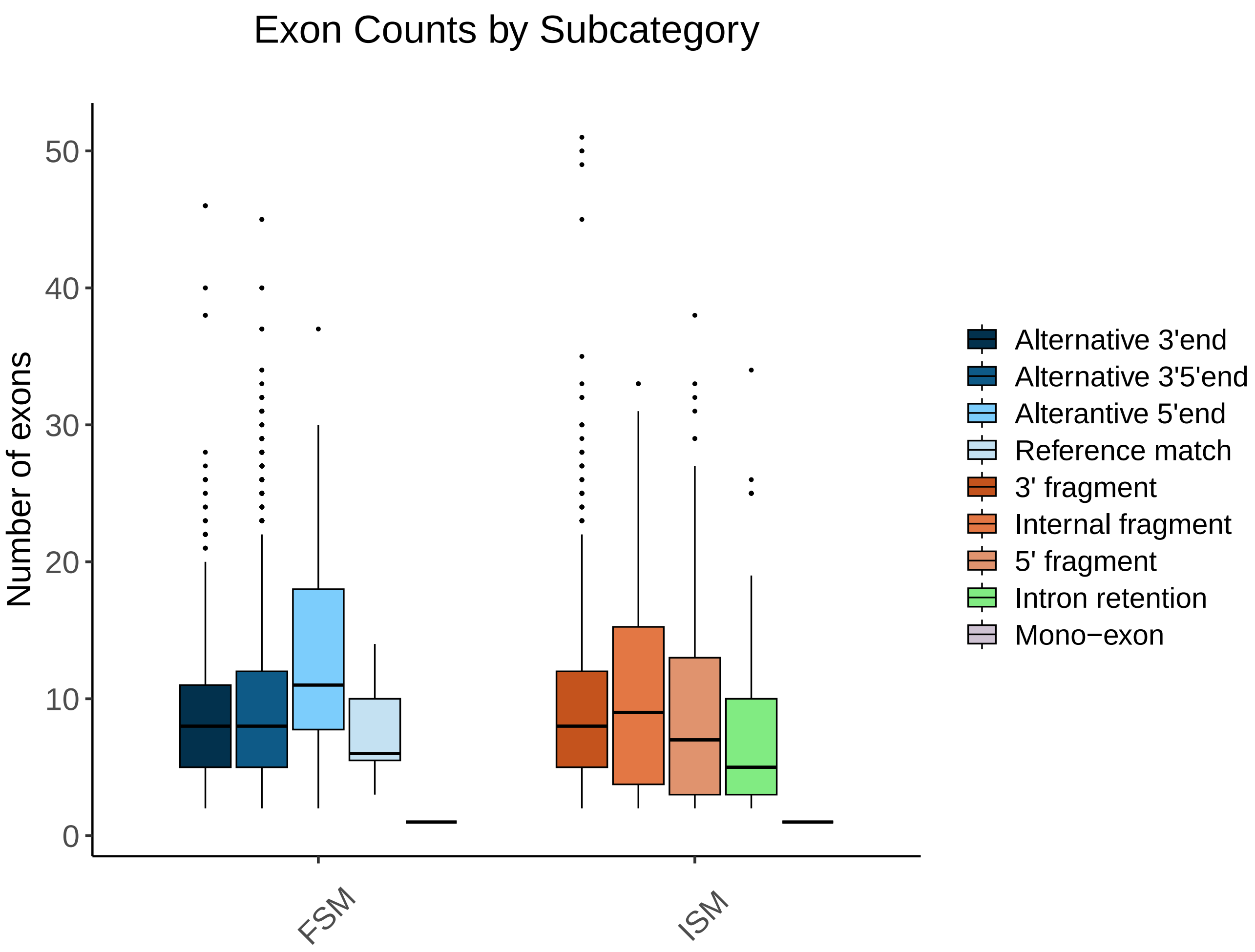
Exon Counts by Subcategory
Number of exons
0
10
20
30
40
50
FSM
ISM
Alternative 3'end
Alternative 3'5'end
Alterantive 5'end
Reference match
3' fragment
Internal fragment
5' fragment
Intron retention
Mono−exon

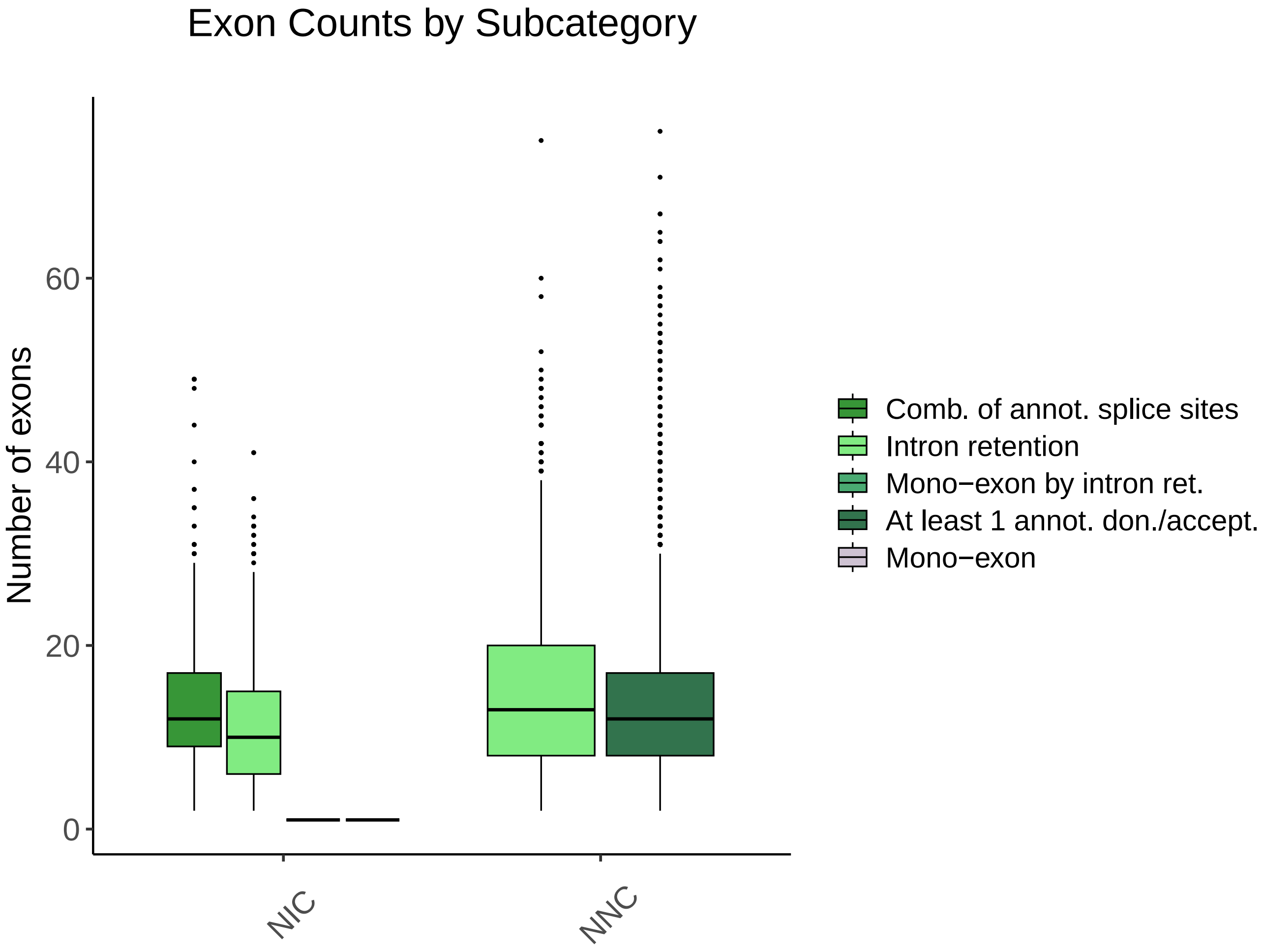
Exon Counts by Subcategory
Number of exons
0
20
40
60
NIC
NNC
Comb. of annot. splice sites
Intron retention
Mono−exon by intron ret.
At least 1 annot. don./accept.
Mono−exon

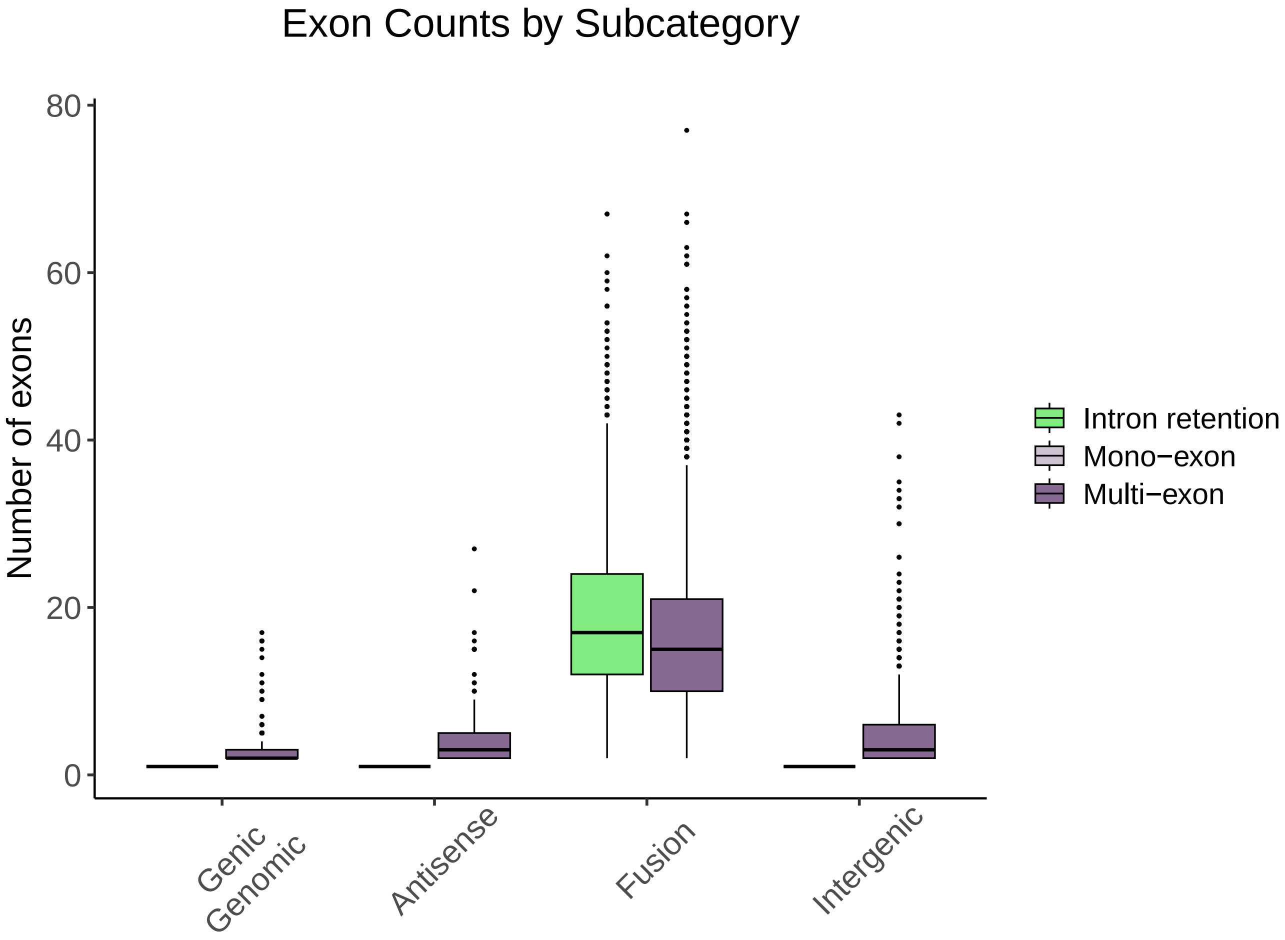
Exon Counts by Subcategory
Number of exons
80
60
40
20
0
Genic
Genomic
Antisense
Fusion
Intergenic
Intron retention
Mono−exon
Multi−exon

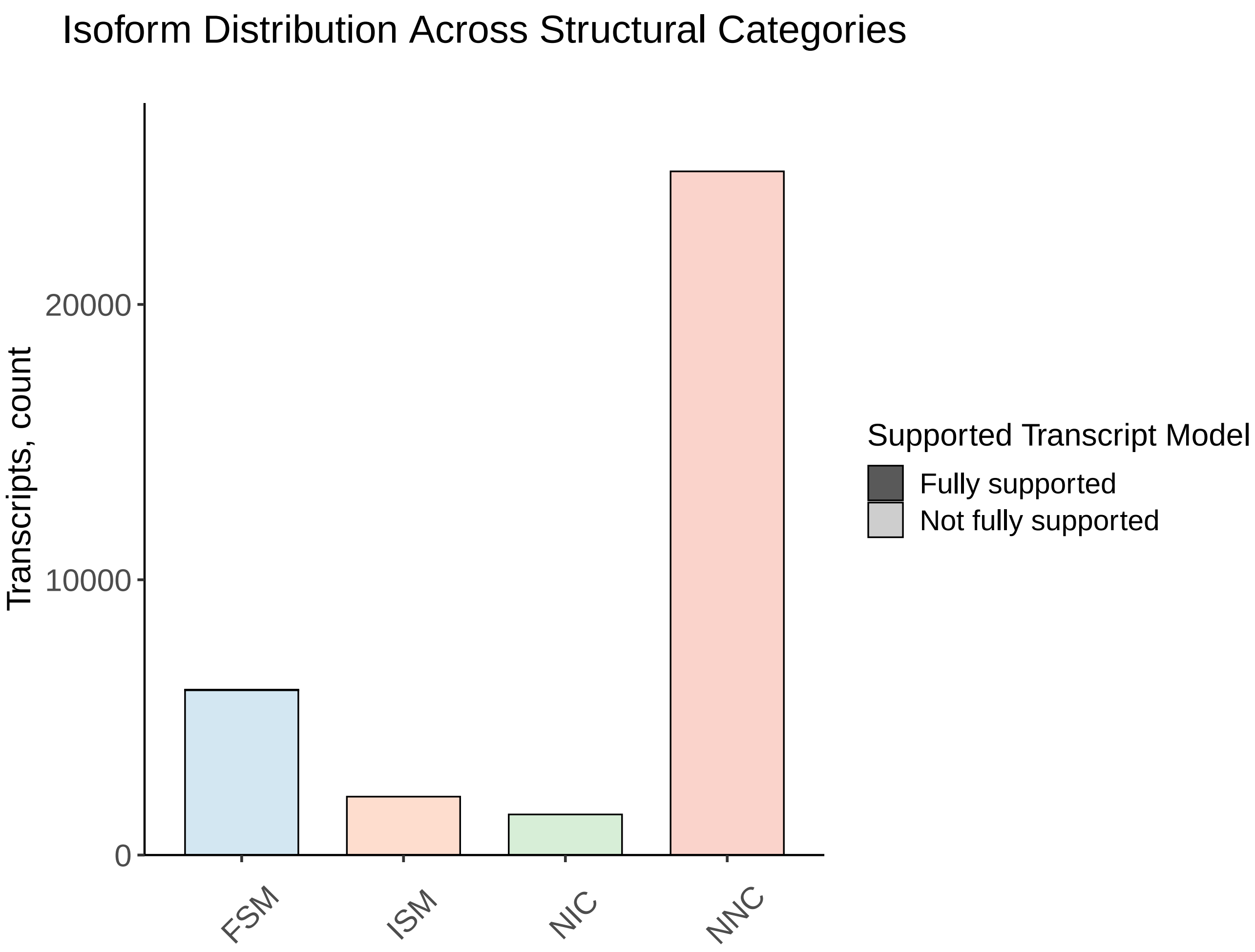

Isoform Distribution Across Structural Categories
Transcripts, count
20000
10000
0
FSM
ISM
NIC
NNC
Supported Transcript Model
Fully supported
Not fully supported

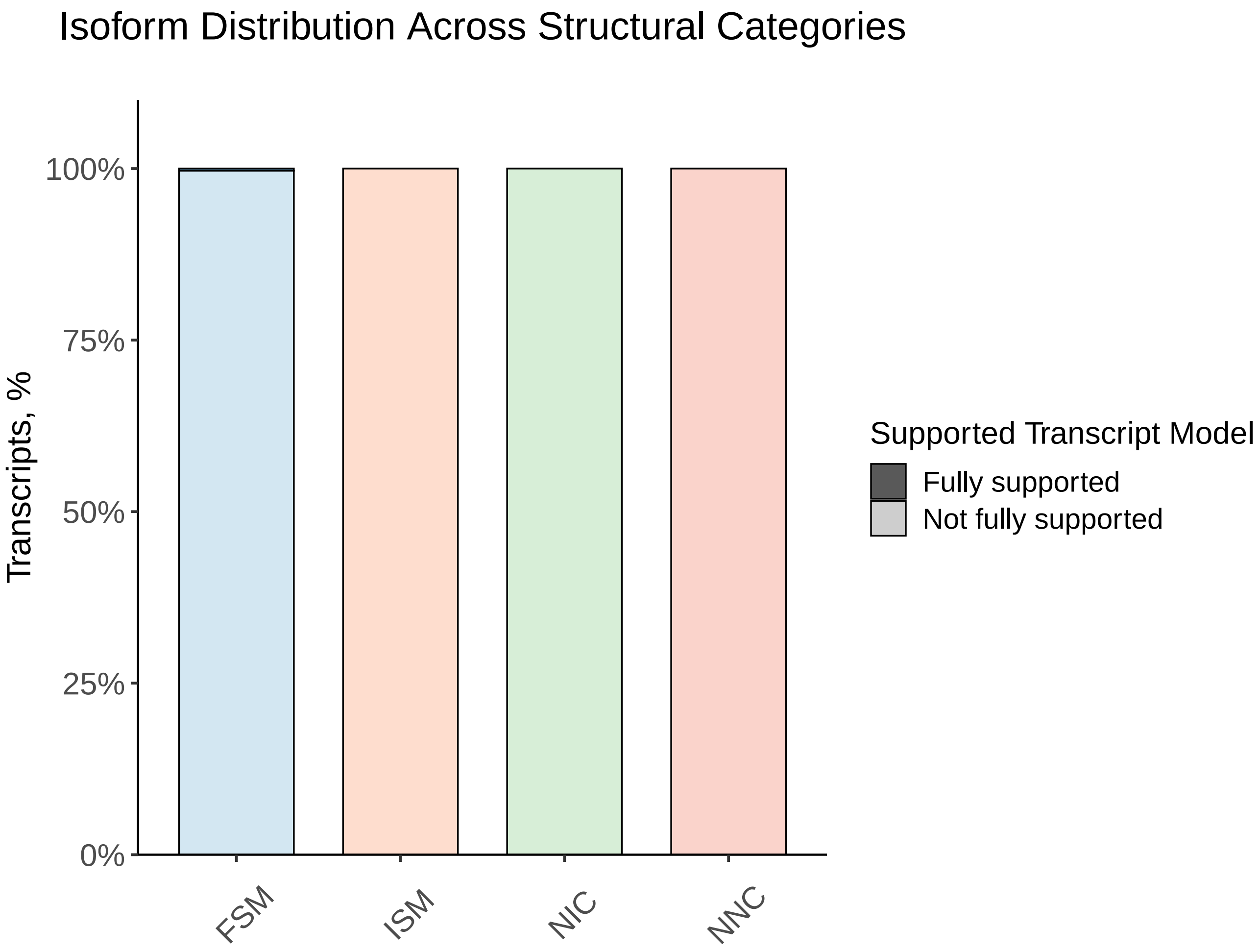

Isoform Distribution Across Structural Categories
Transcripts, %
100%
75%
50%
25%
0%
FSM
ISM
NIC
NNC
Supported Transcript Model
Fully supported
Not fully supported

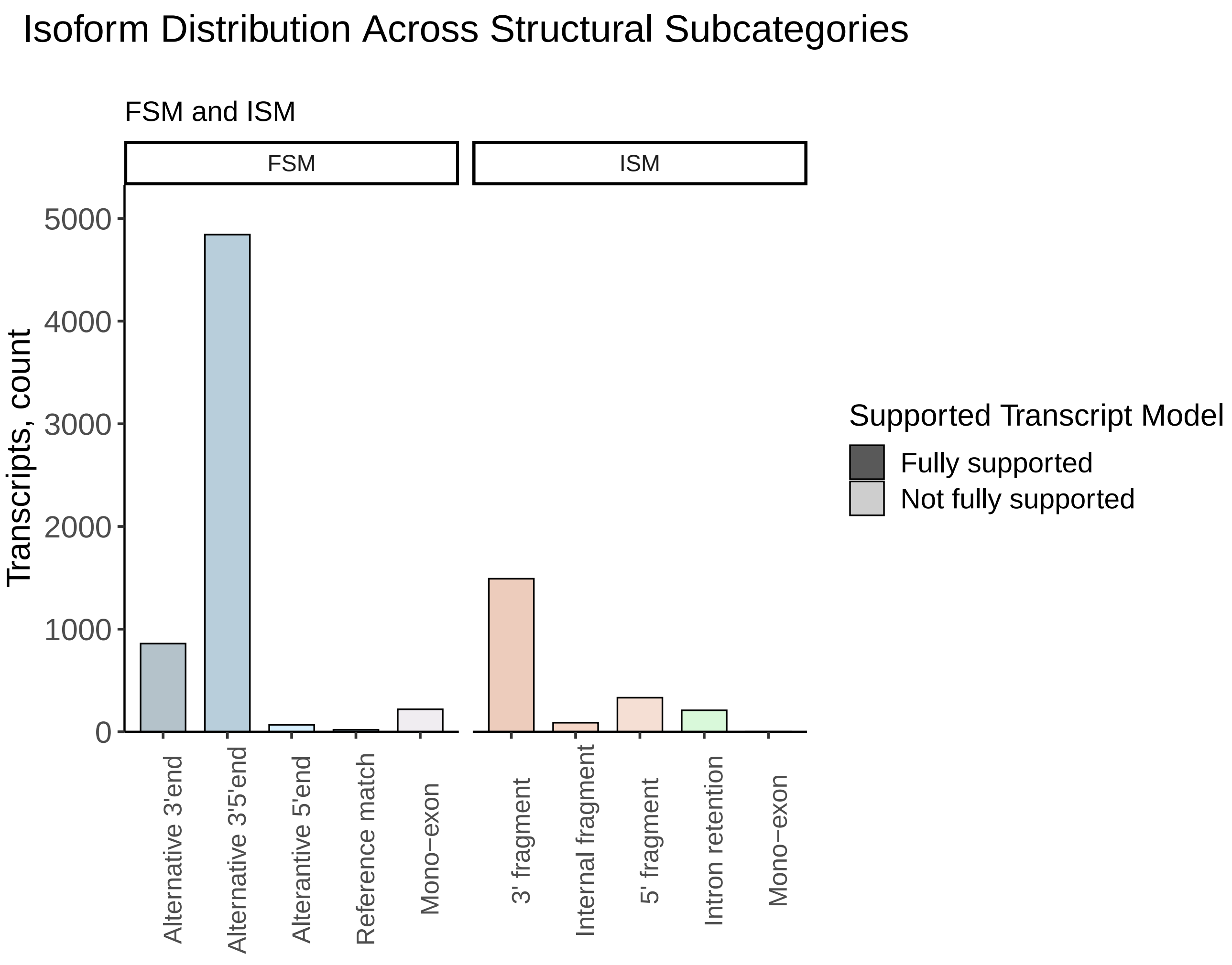
Isoform Distribution Across Structural Subcategories
FSM and ISM
FSM
ISM
Transcripts, count
5000
4000
3000
2000
1000
0
Alternative 3'end
Alternative 3'5'end
Alterantive 5'end
Reference match
Mono−exon
3' fragment
Internal fragment
5' fragment
Intron retention
Mono−exon
Supported Transcript Model
Fully supported
Not fully supported

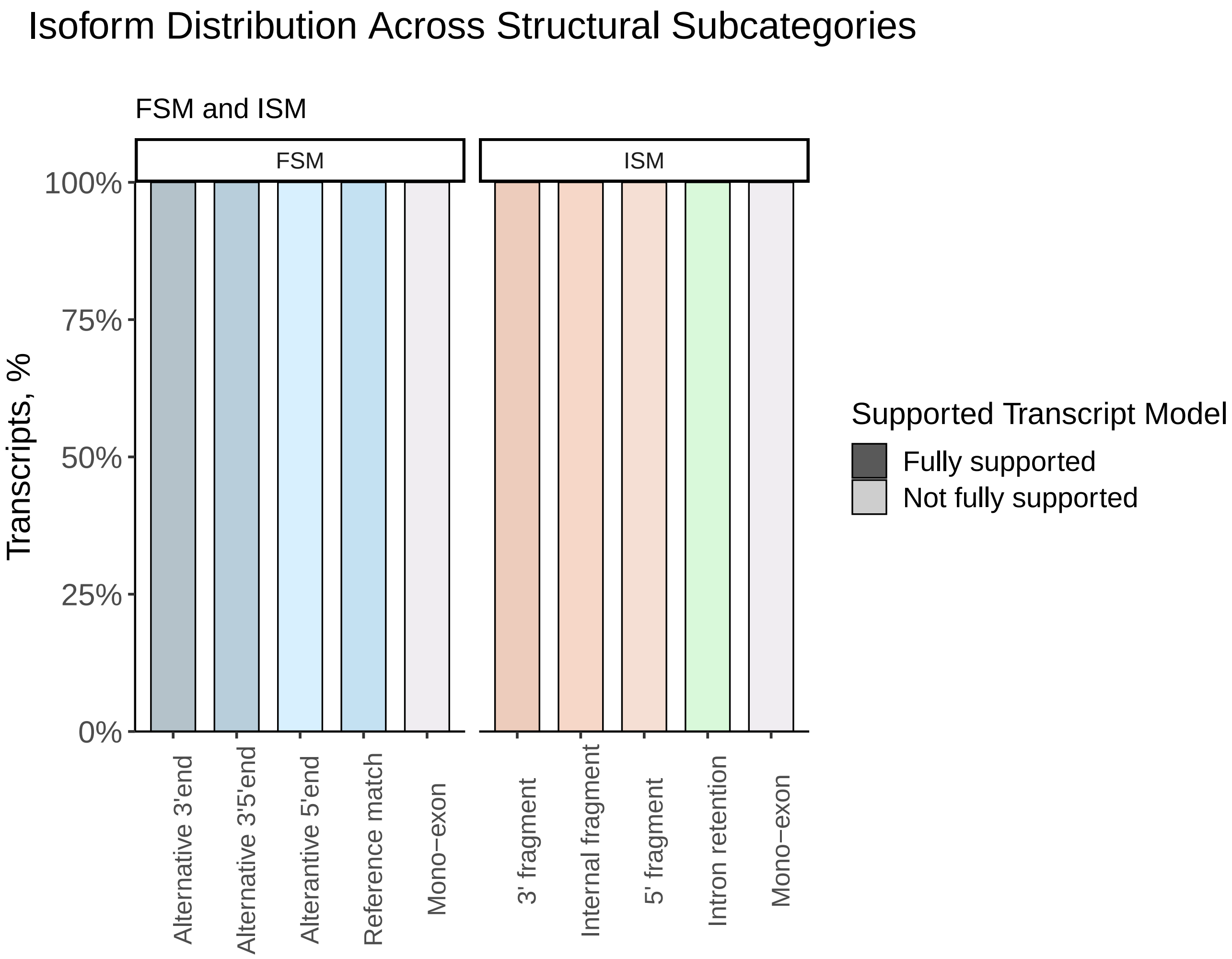

Isoform Distribution Across Structural Subcategories
FSM and ISM
FSM
ISM
100%
75%
50%
25%
0%
Transcripts, %
Alternative 3'end
Alternative 3'5'end
Alterantive 5'end
Reference match
Mono−exon
3' fragment
Internal fragment
5' fragment
Intron retention
Mono−exon
Supported Transcript Model
Fully supported
Not fully supported

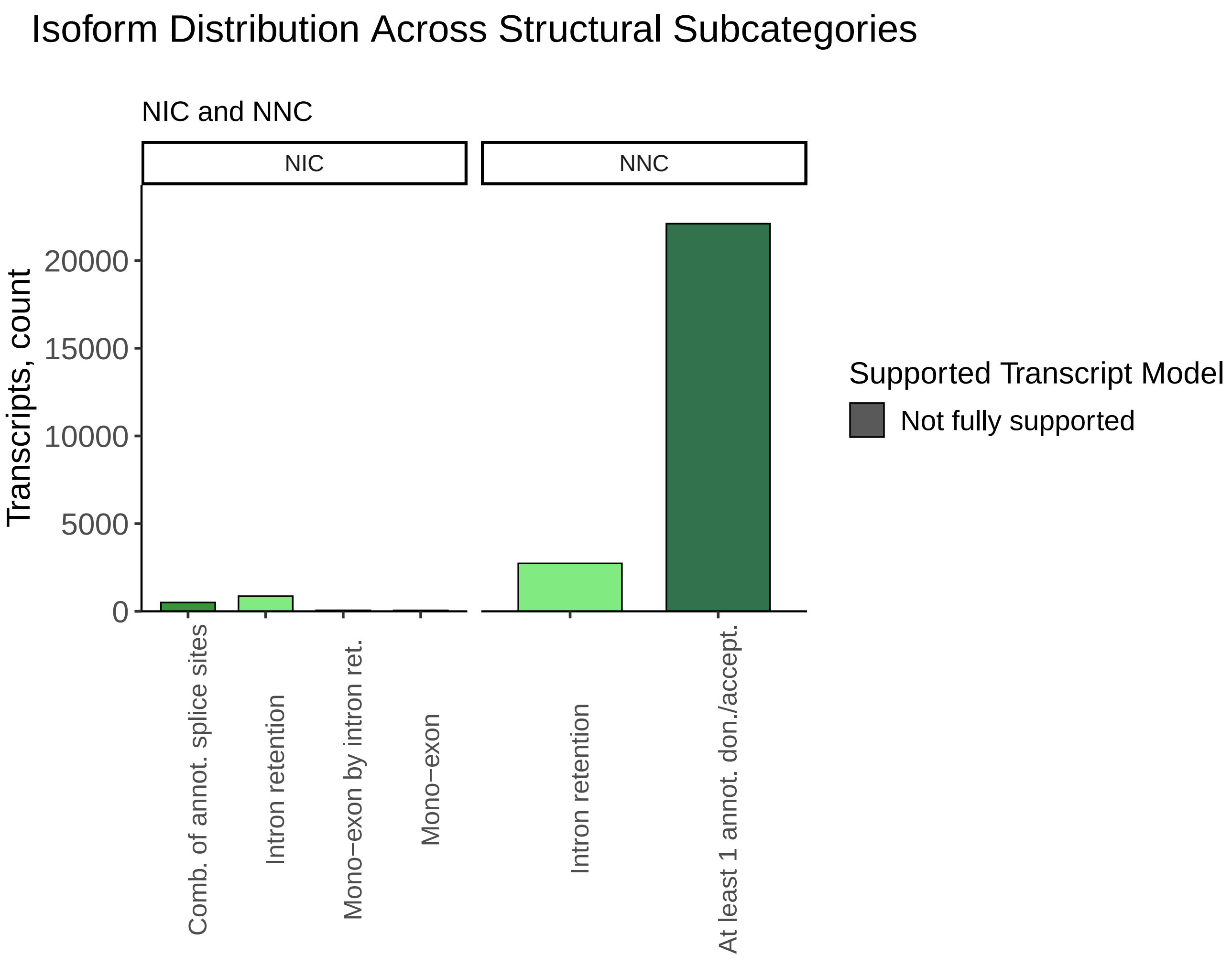

Isoform Distribution Across Structural Subcategories
NIC and NNC
NIC
NNC
Transcripts, count
0
5000
10000
15000
20000
Comb. of annot. splice sites
Intron retention
Mono−exon by intron ret.
Mono−exon
Intron retention
At least 1 annot. don./accept.
Supported Transcript Model
Not fully supported

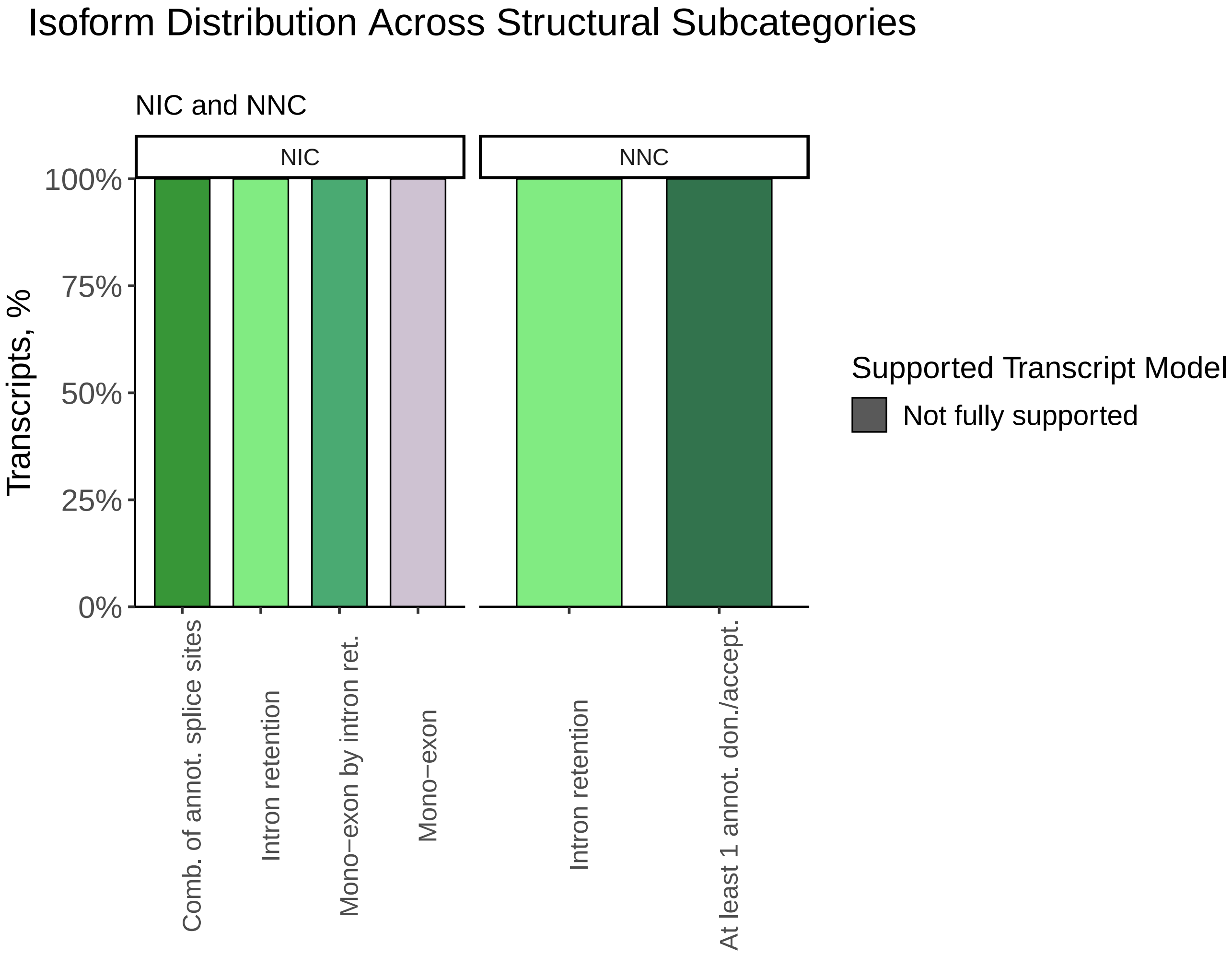
Isoform Distribution Across Structural Subcategories
NIC and NNC
NIC
NNC
100%
75%
50%
25%
0%
Transcripts, %
Comb. of annot. splice sites
Intron retention
Mono−exon by intron ret.
Mono−exon
Intron retention
At least 1 annot. don./accept.
Supported Transcript Model
Not fully supported

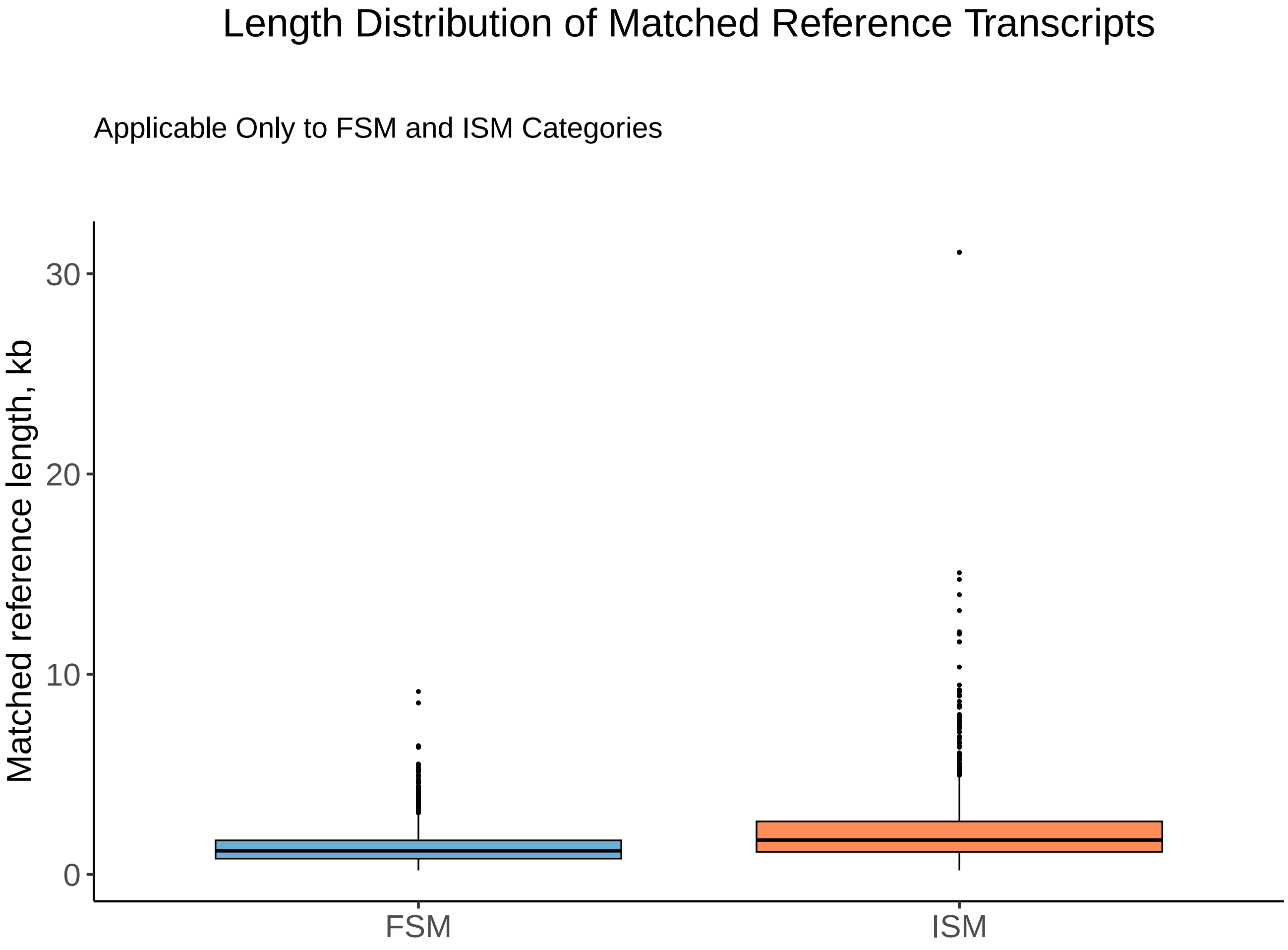

Length Distribution of Matched Reference Transcripts
Applicable Only to FSM and ISM Categories
Matched reference length, kb
30
20
10
0
FSM
ISM

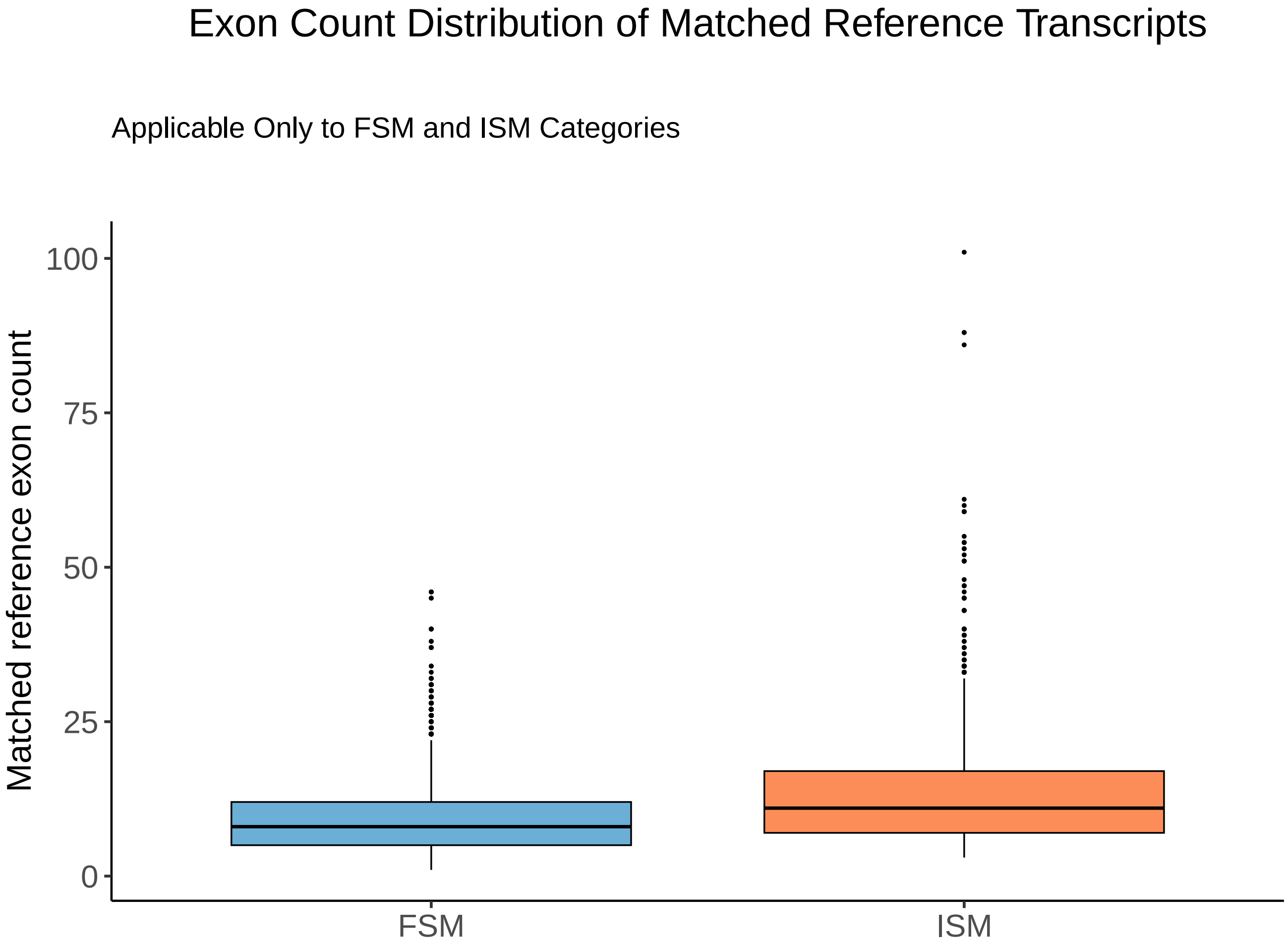

Exon Count Distribution of Matched Reference Transcripts
Applicable Only to FSM and ISM Categories
Matched reference exon count
100
75
50
25
0
FSM
ISM

Splice Junction Characterization

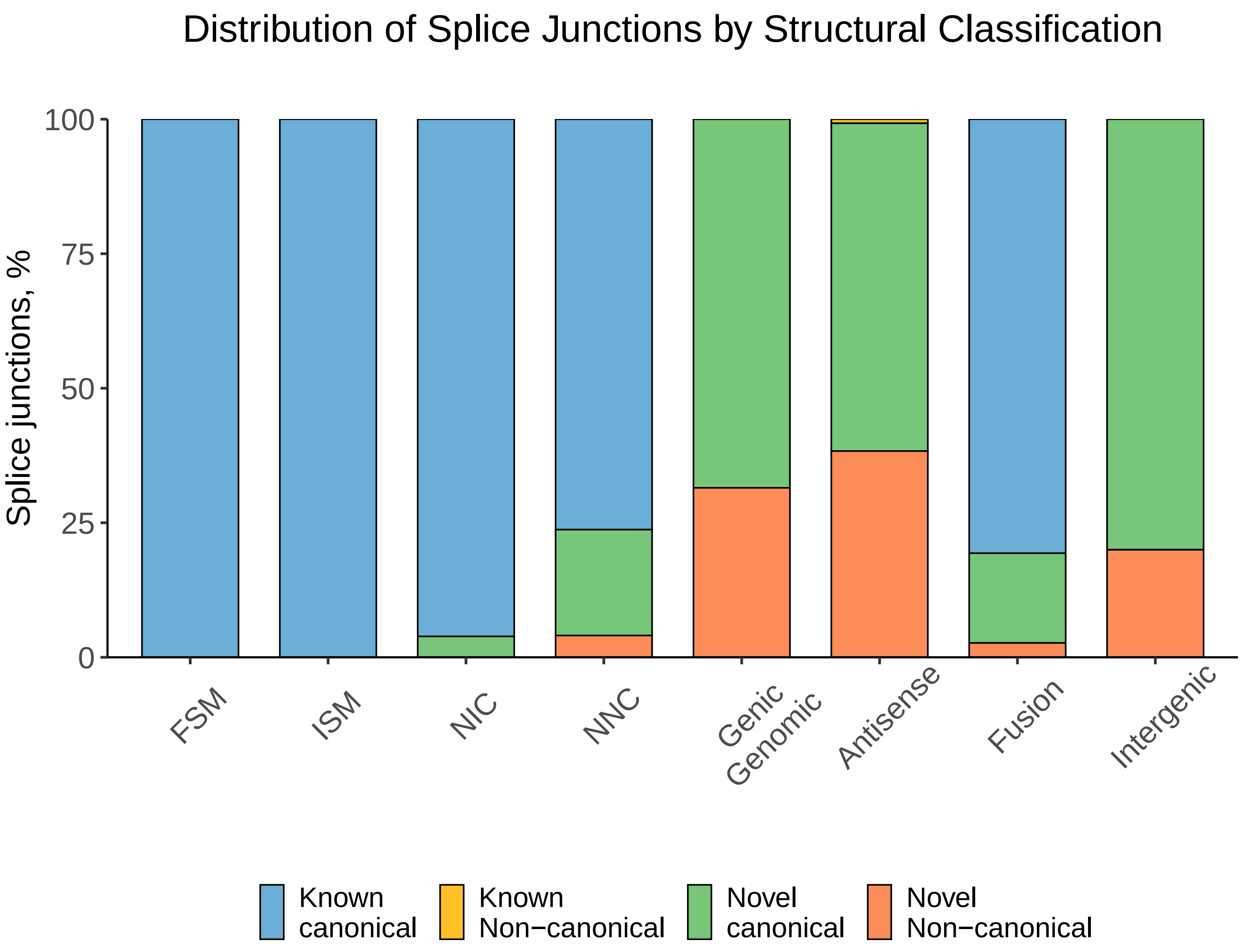
Distribution of Splice Junctions by Structural Classification
Splice junctions, %
100
75
50
25
0
FSM
ISM
NIC
NNC
Genic
Genomic
Antisense
Fusion
Intergenic
Known
canonical
Known
Non−canonical
Novel
canonical
Novel
Non−canonical

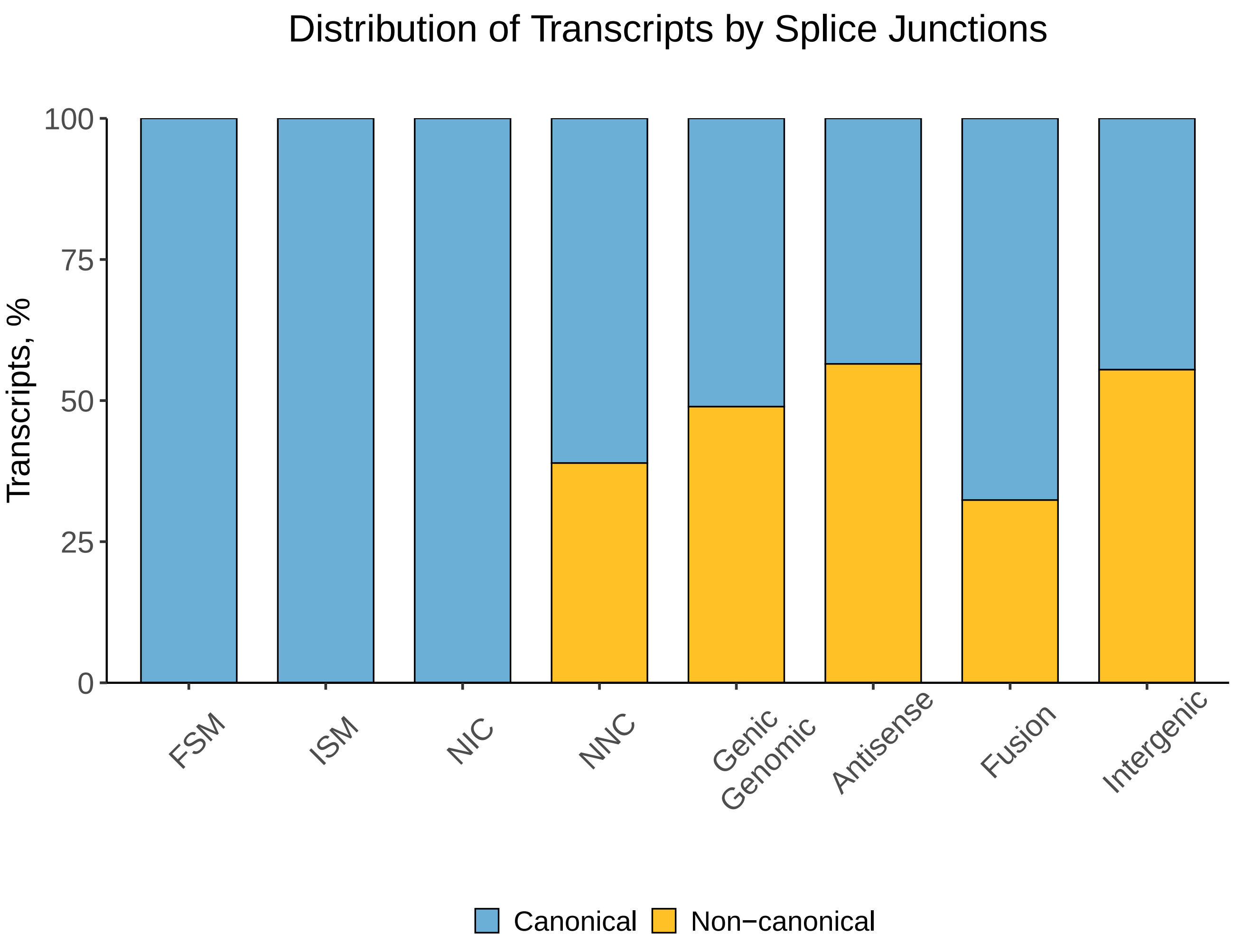

Distribution of Transcripts by Splice Junctions
Transcripts, %
100
75
50
25
0
FSM
ISM
NIC
NNC
Genic
Genomic
Antisense
Fusion
Intergenic
Canonical
Non−canonical

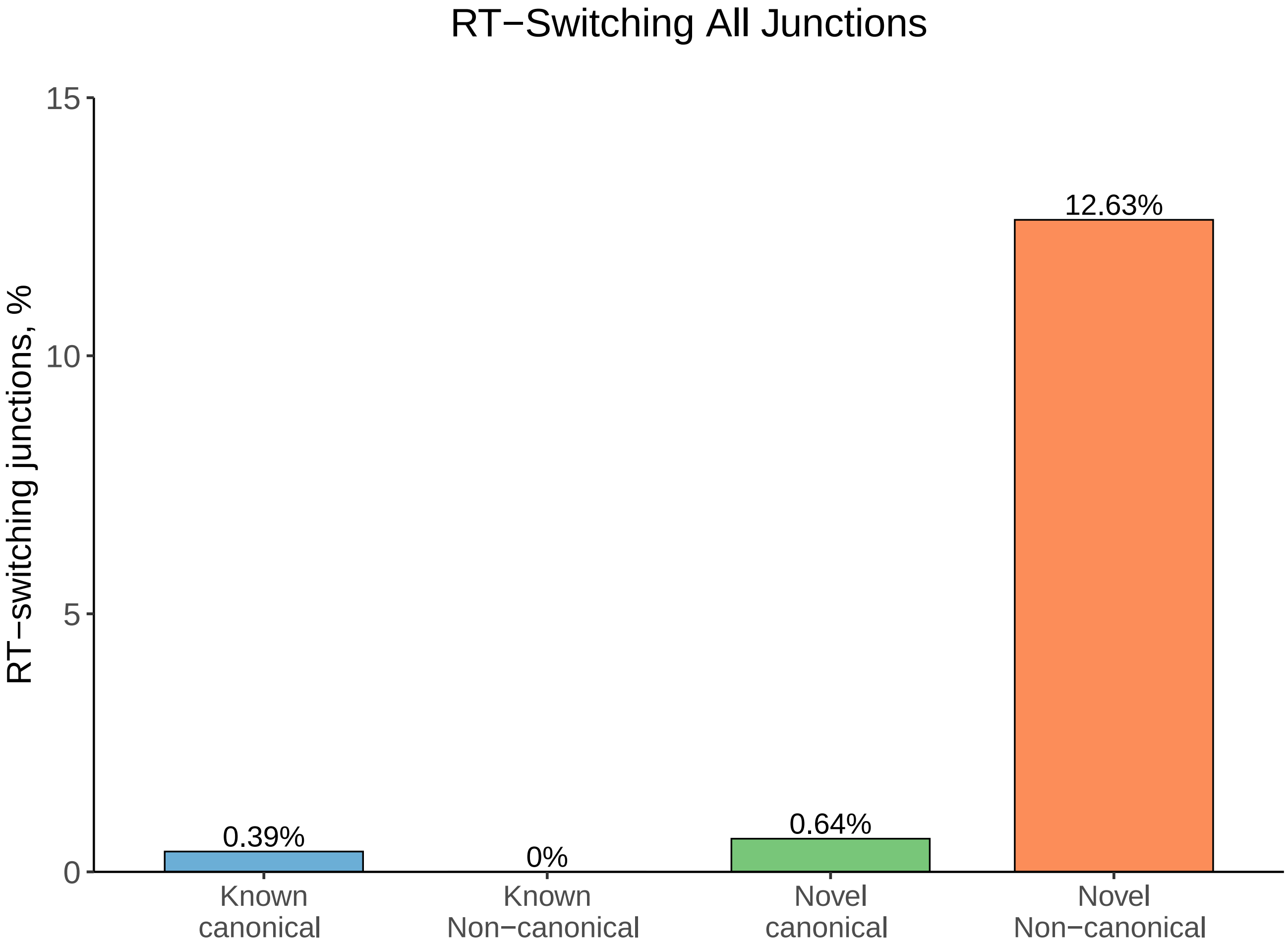

RT−Switching All Junctions
RT−switching junctions, %
15
10
5
0
12.63%
0.39%
0%
0.64%
Known canonical
Known Non−canonical
Novel canonical
Novel Non−canonical

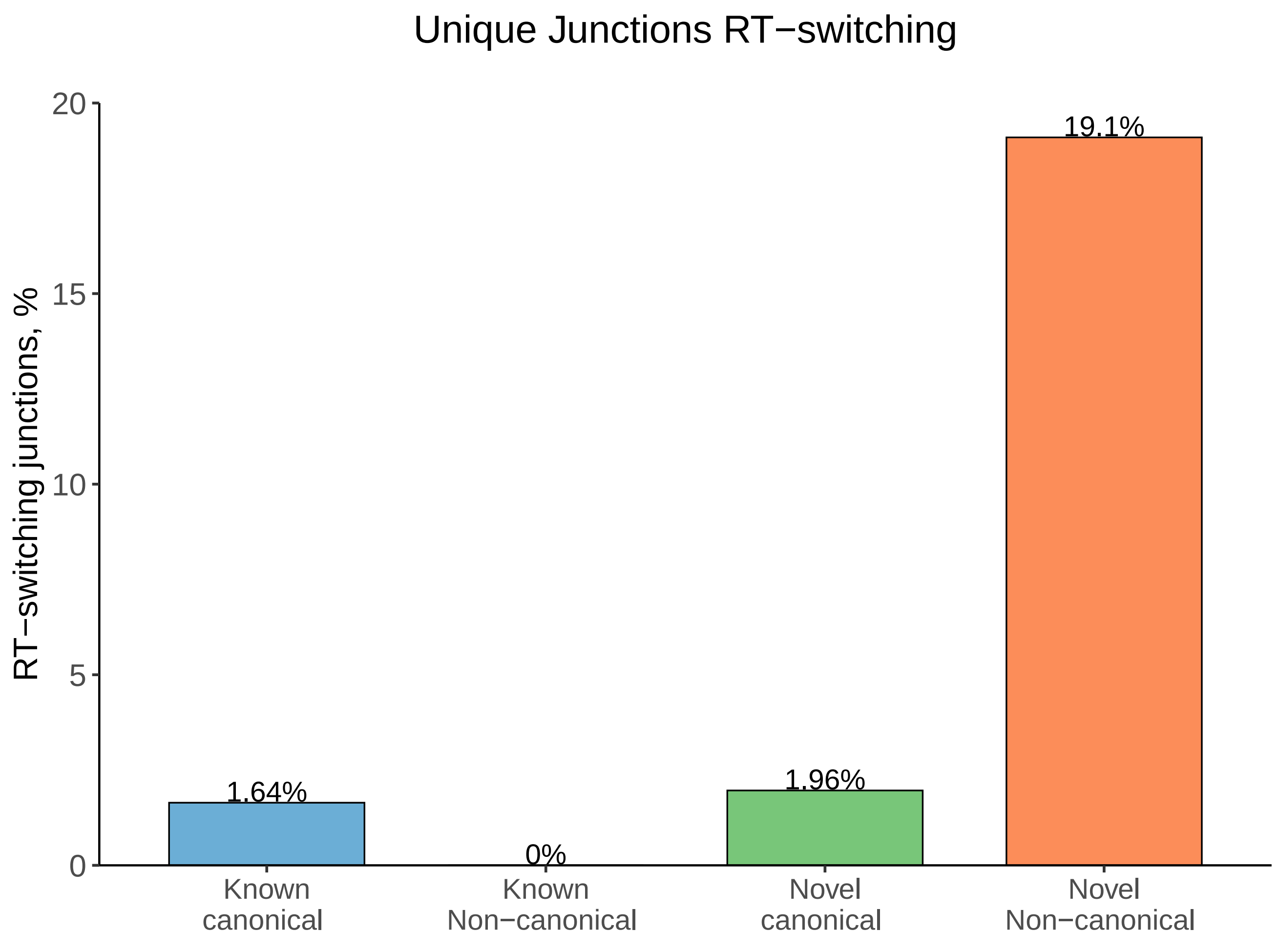

Unique Junctions RT−switching
RT−switching junctions, %
20
15
10
5
0
1.64%
0%
1.96%
19.1%
Known canonical
Known Non−canonical
Novel canonical
Novel Non−canonical

Comparison With Annotated TSS and TTS

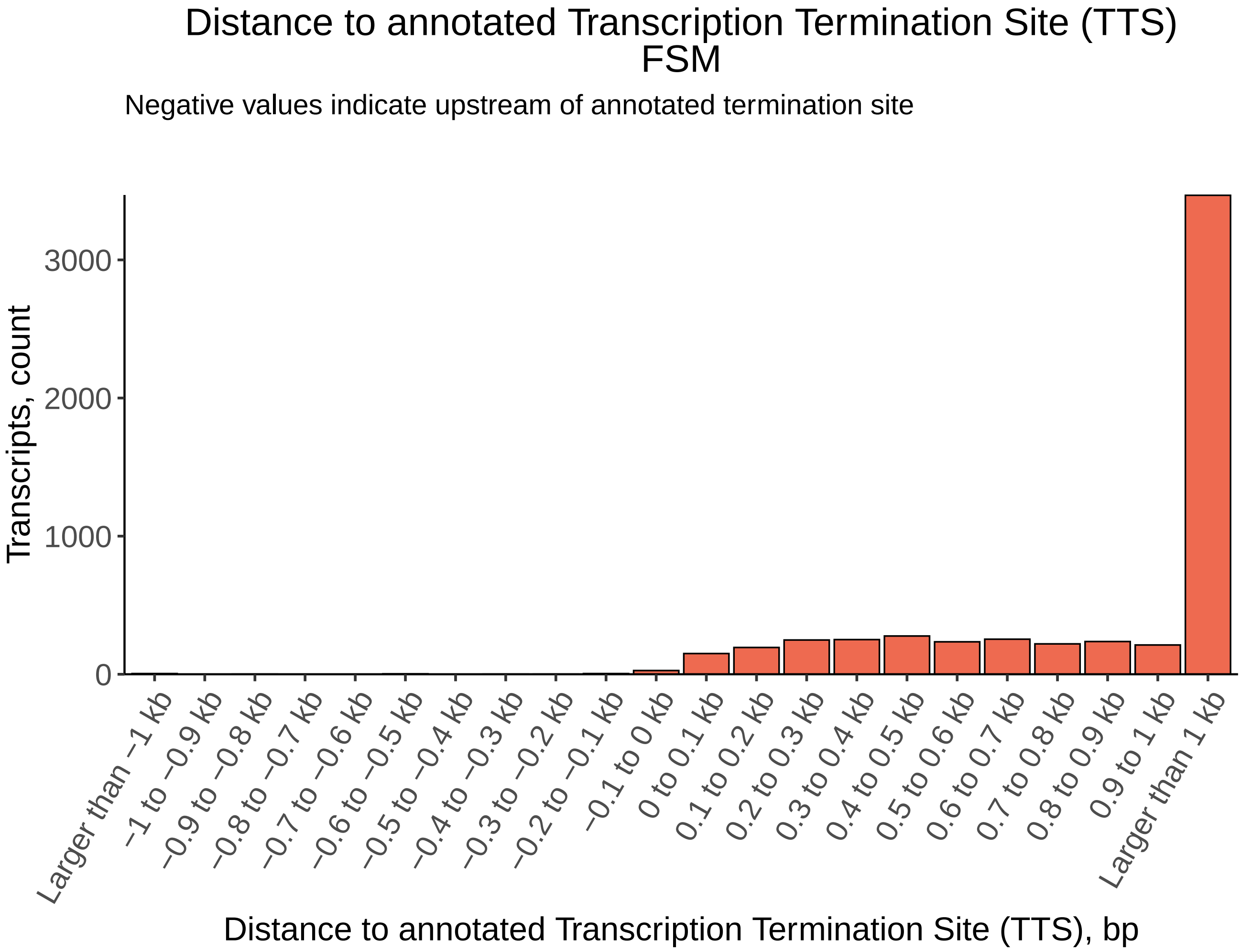
Distance to annotated Transcription Termination Site (TTS)
FSM
Negative values indicate upstream of annotated termination site
Transcripts, count
3000
2000
1000
0
Larger than −1 kb
−1 to −0.9 kb
−0.9 to −0.8 kb
−0.8 to −0.7 kb
−0.7 to −0.6 kb
−0.6 to −0.5 kb
−0.5 to −0.4 kb
−0.4 to −0.3 kb
−0.3 to −0.2 kb
−0.2 to −0.1 kb
−0.1 to 0 kb
0 to 0.1 kb
0.1 to 0.2 kb
0.2 to 0.3 kb
0.3 to 0.4 kb
0.4 to 0.5 kb
0.5 to 0.6 kb
0.6 to 0.7 kb
0.7 to 0.8 kb
0.8 to 0.9 kb
0.9 to 1 kb
Larger than 1 kb
Distance to annotated Transcription Termination Site (TTS), bp

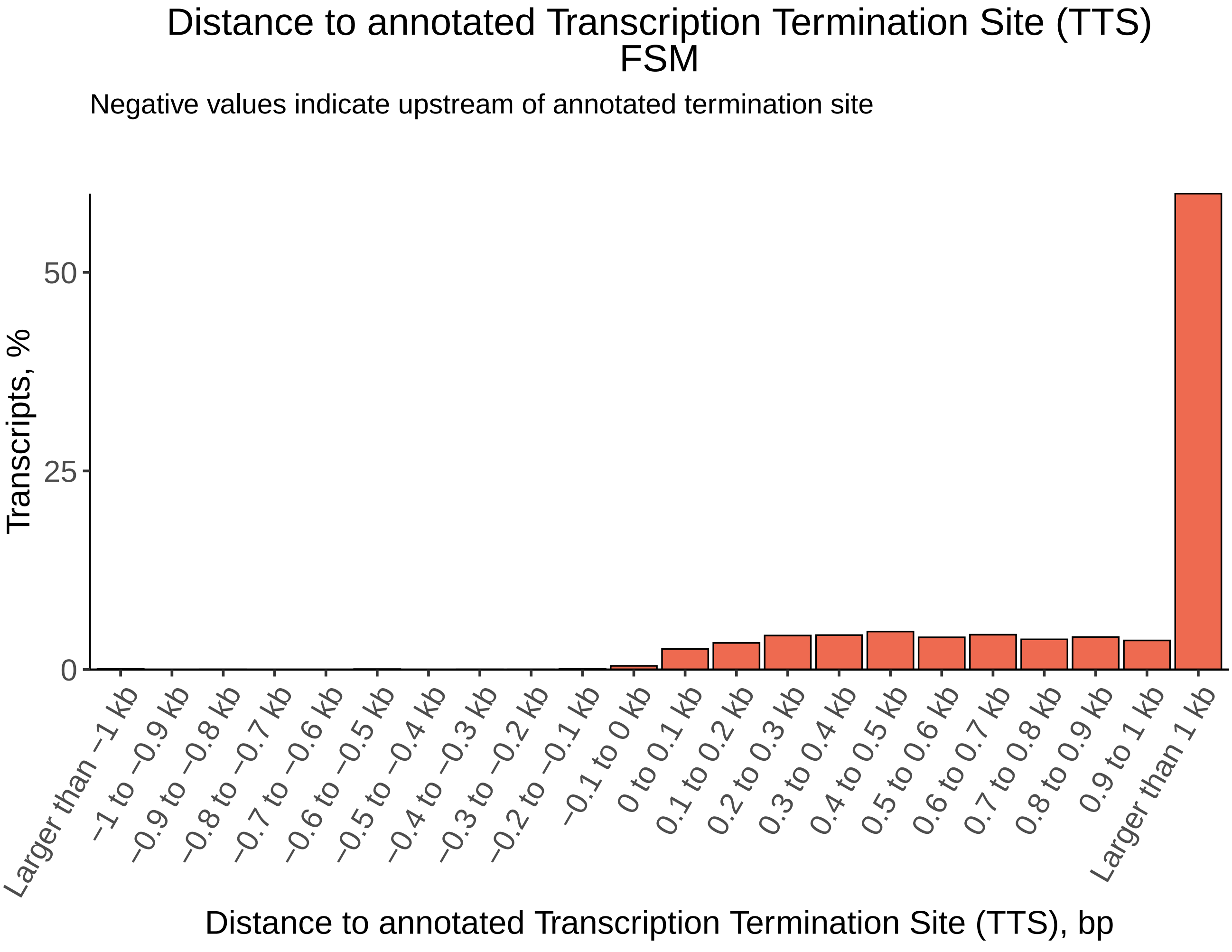
Distance to annotated Transcription Termination Site (TTS)
FSM
Negative values indicate upstream of annotated termination site
Transcripts, %
50
25
0
Larger than −1 kb
−1 to −0.9 kb
−0.9 to −0.8 kb
−0.8 to −0.7 kb
−0.7 to −0.6 kb
−0.6 to −0.5 kb
−0.5 to −0.4 kb
−0.4 to −0.3 kb
−0.3 to −0.2 kb
−0.2 to −0.1 kb
−0.1 to 0 kb
0 to 0.1 kb
0.1 to 0.2 kb
0.2 to 0.3 kb
0.3 to 0.4 kb
0.4 to 0.5 kb
0.5 to 0.6 kb
0.6 to 0.7 kb
0.7 to 0.8 kb
0.8 to 0.9 kb
0.9 to 1 kb
Larger than 1 kb
Distance to annotated Transcription Termination Site (TTS), bp

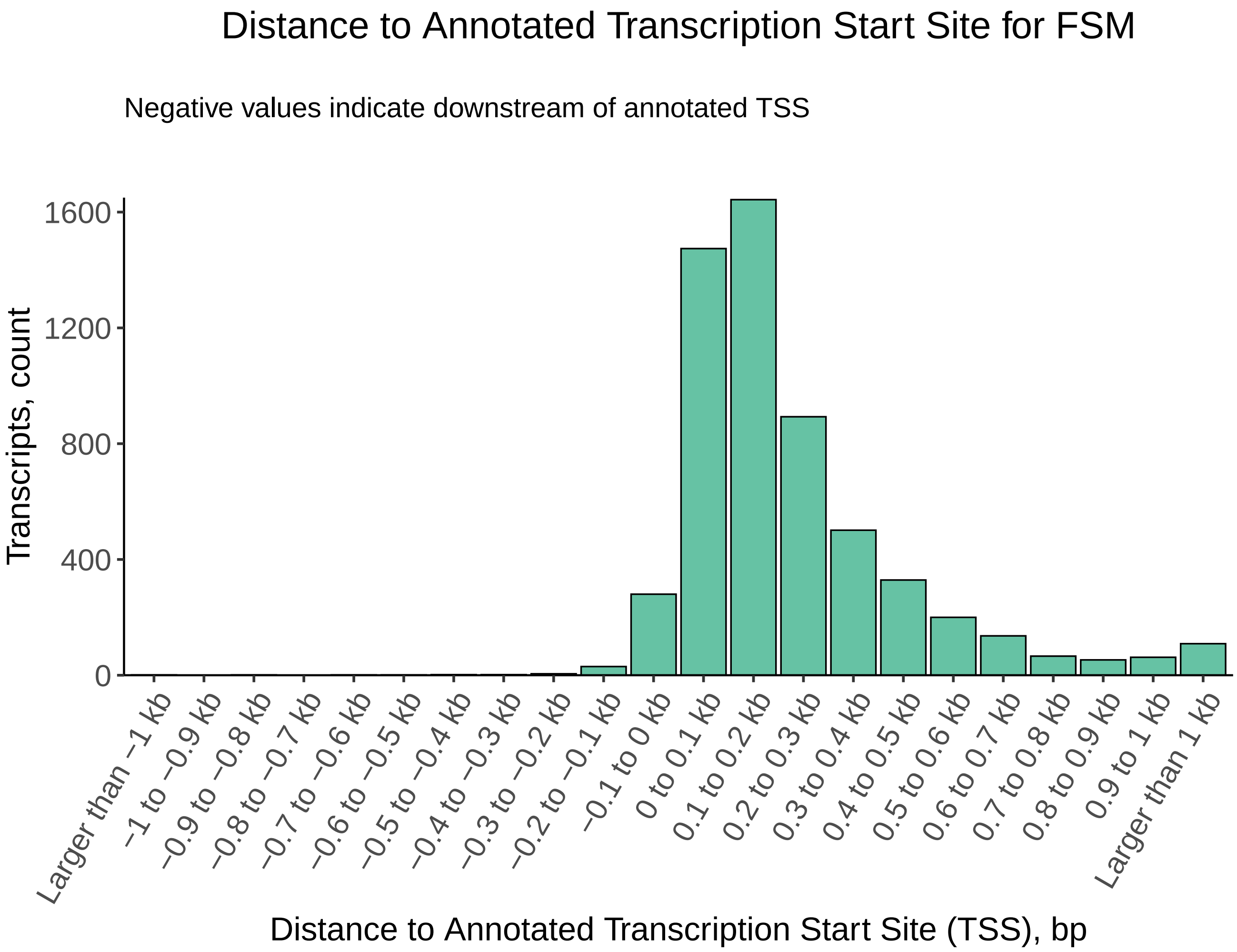
Distance to Annotated Transcription Start Site for FSM
Negative values indicate downstream of annotated TSS
Transcripts, count
0
400
800
1200
1600
Larger than −1 kb
−1 to −0.9 kb
−0.9 to −0.8 kb
−0.8 to −0.7 kb
−0.7 to −0.6 kb
−0.6 to −0.5 kb
−0.5 to −0.4 kb
−0.4 to −0.3 kb
−0.3 to −0.2 kb
−0.2 to −0.1 kb
−0.1 to 0 kb
0 to 0.1 kb
0.1 to 0.2 kb
0.2 to 0.3 kb
0.3 to 0.4 kb
0.4 to 0.5 kb
0.5 to 0.6 kb
0.6 to 0.7 kb
0.7 to 0.8 kb
0.8 to 0.9 kb
0.9 to 1 kb
Larger than 1 kb
Distance to Annotated Transcription Start Site (TSS), bp

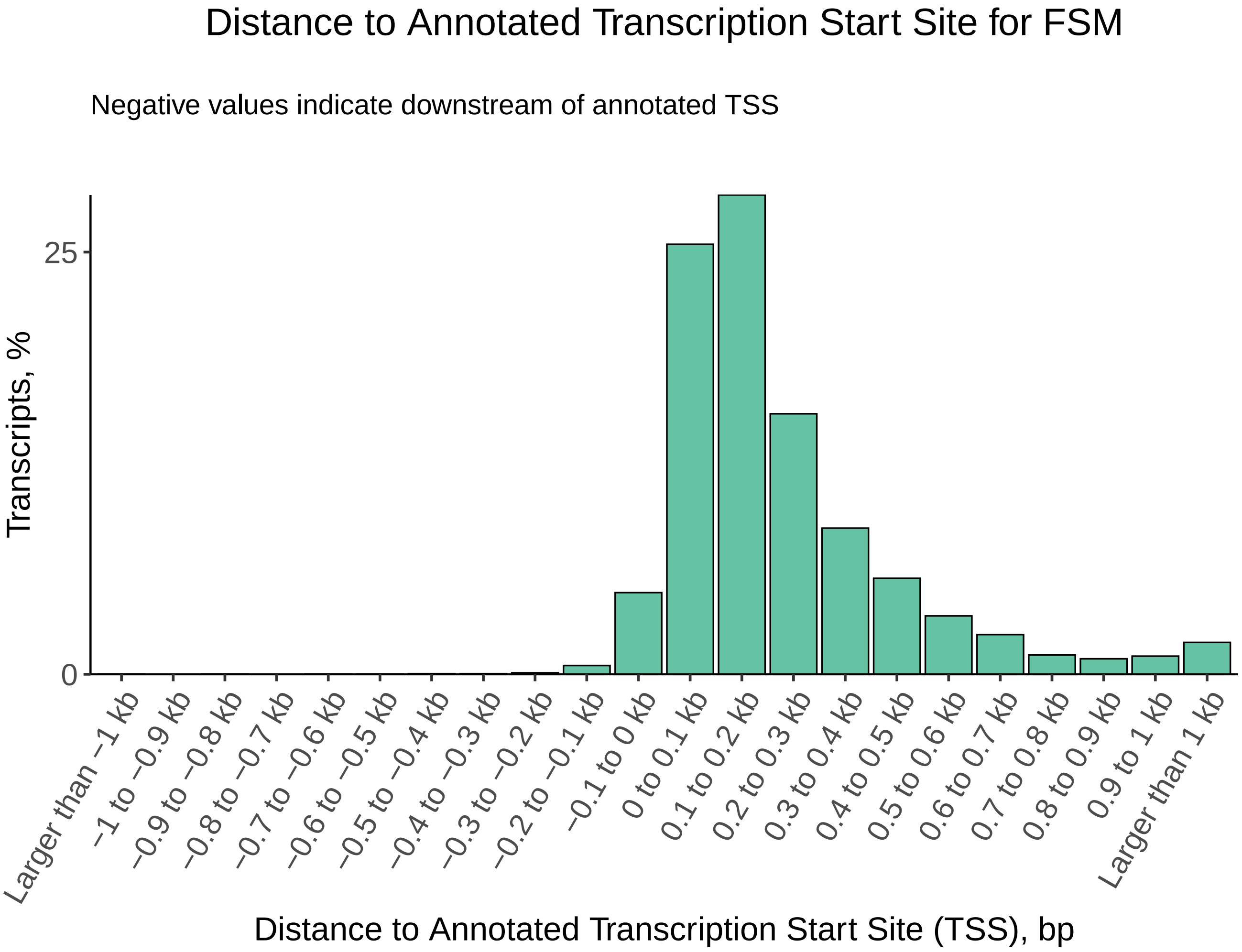
Distance to Annotated Transcription Start Site for FSM
Negative values indicate downstream of annotated TSS
Transcripts, %
25
0
Larger than −1 kb
−1 to −0.9 kb
−0.9 to −0.8 kb
−0.8 to −0.7 kb
−0.7 to −0.6 kb
−0.6 to −0.5 kb
−0.5 to −0.4 kb
−0.4 to −0.3 kb
−0.3 to −0.2 kb
−0.2 to −0.1 kb
−0.1 to 0 kb
0 to 0.1 kb
0.1 to 0.2 kb
0.2 to 0.3 kb
0.3 to 0.4 kb
0.4 to 0.5 kb
0.5 to 0.6 kb
0.6 to 0.7 kb
0.7 to 0.8 kb
0.8 to 0.9 kb
0.9 to 1 kb
Larger than 1 kb
Distance to Annotated Transcription Start Site (TSS), bp

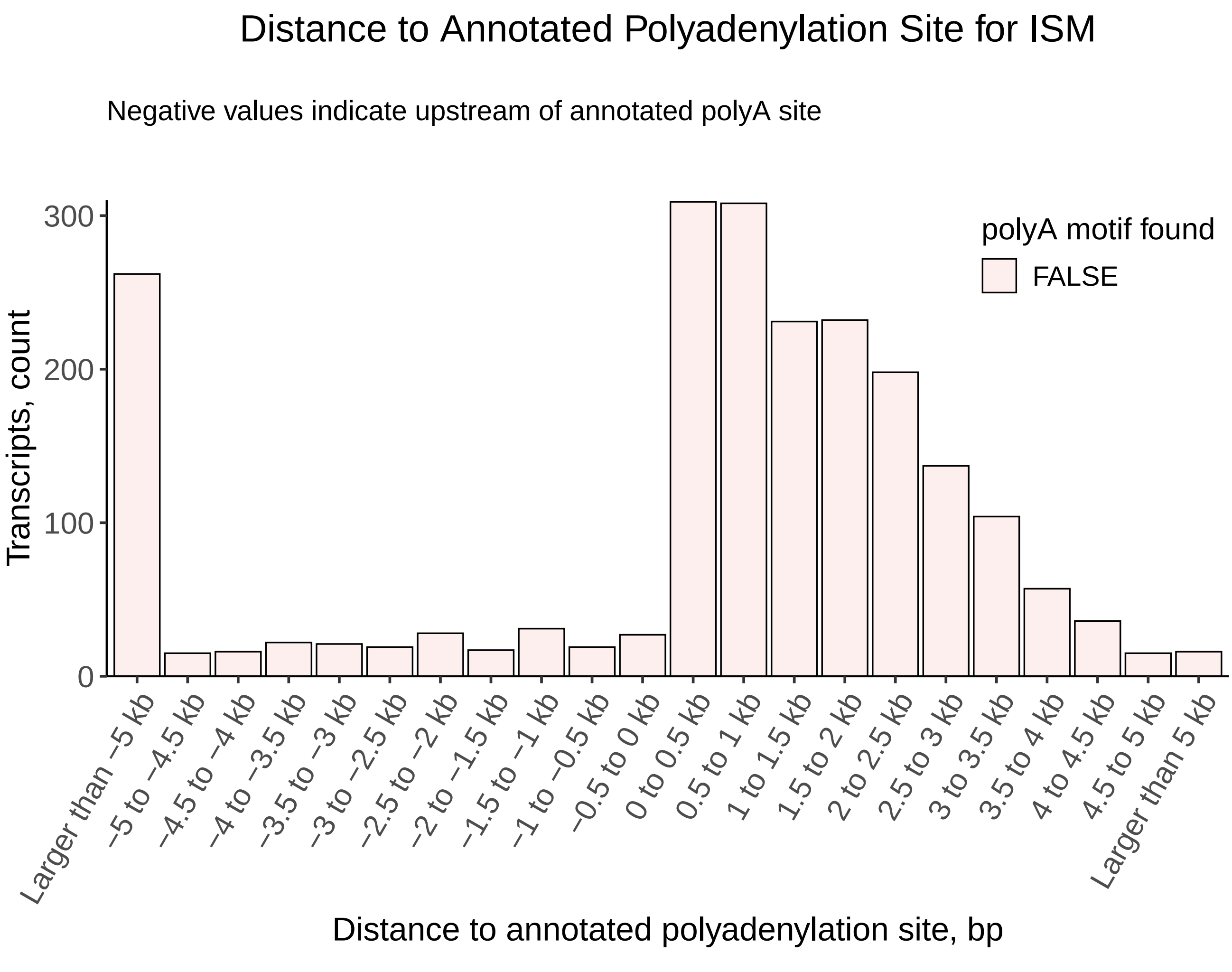

Distance to Annotated Polyadenylation Site for ISM
Negative values indicate upstream of annotated polyA site
Transcripts, count
300
200
100
0
polyA motif found
FALSE
Larger than −5 kb
−5 to −4.5 kb
−4.5 to −4 kb
−4 to −3.5 kb
−3.5 to −3 kb
−3 to −2.5 kb
−2.5 to −2 kb
−2 to −1.5 kb
−1.5 to −1 kb
−1 to −0.5 kb
−0.5 to 0 kb
0 to 0.5 kb
0.5 to 1 kb
1 to 1.5 kb
1.5 to 2 kb
2 to 2.5 kb
2.5 to 3 kb
3 to 3.5 kb
3.5 to 4 kb
4 to 4.5 kb
4.5 to 5 kb
Larger than 5 kb
Distance to annotated polyadenylation site, bp

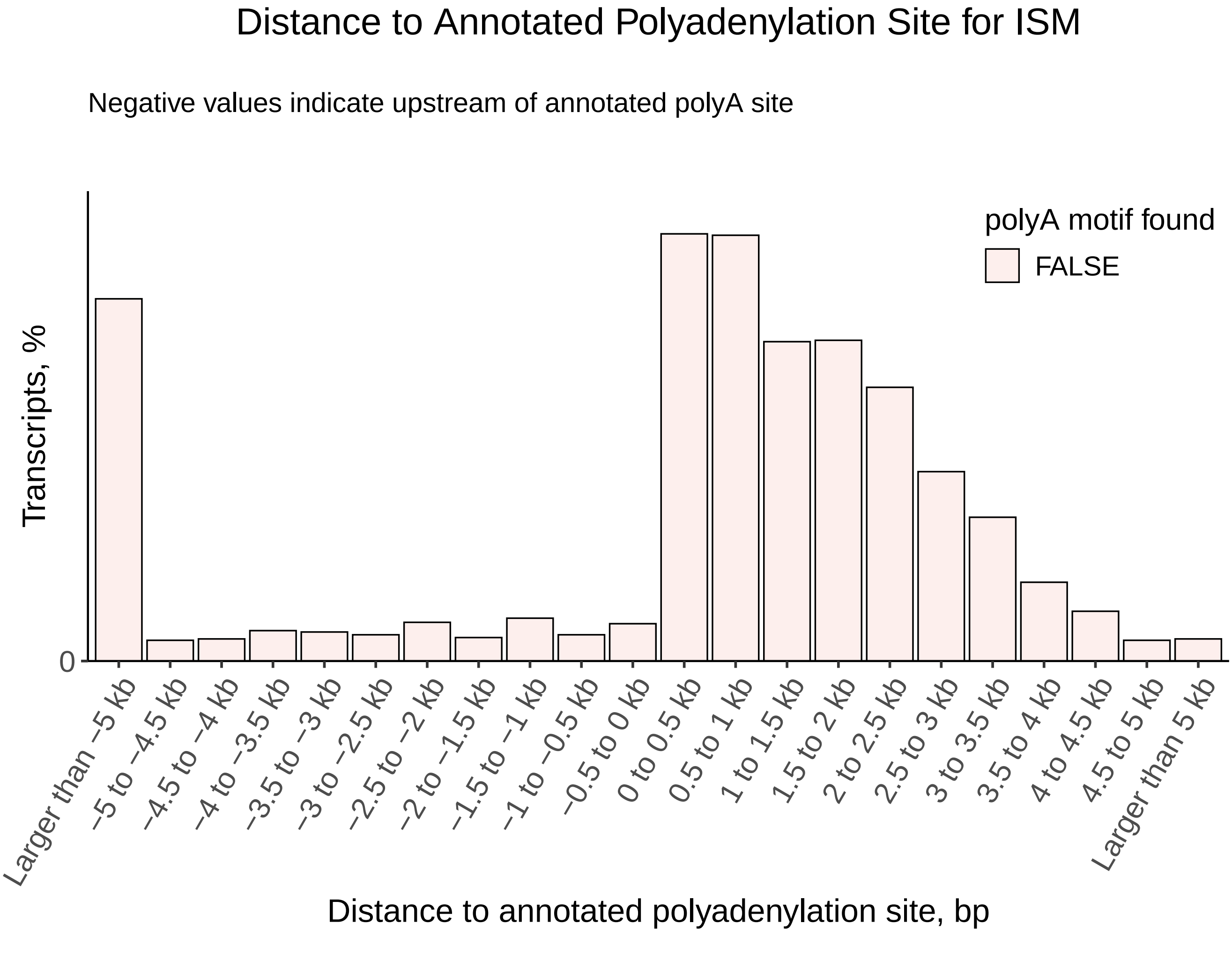

Distance to Annotated Polyadenylation Site for ISM
Negative values indicate upstream of annotated polyA site
polyA motif found
FALSE
Transcripts, %
0
Larger than −5 kb
−5 to −4.5 kb
−4.5 to −4 kb
−4 to −3.5 kb
−3.5 to −3 kb
−3 to −2.5 kb
−2.5 to −2 kb
−2 to −1.5 kb
−1.5 to −1 kb
−1 to −0.5 kb
−0.5 to 0 kb
0 to 0.5 kb
0.5 to 1 kb
1 to 1.5 kb
1.5 to 2 kb
2 to 2.5 kb
2.5 to 3 kb
3 to 3.5 kb
3.5 to 4 kb
4 to 4.5 kb
4.5 to 5 kb
Larger than 5 kb
Distance to annotated polyadenylation site, bp

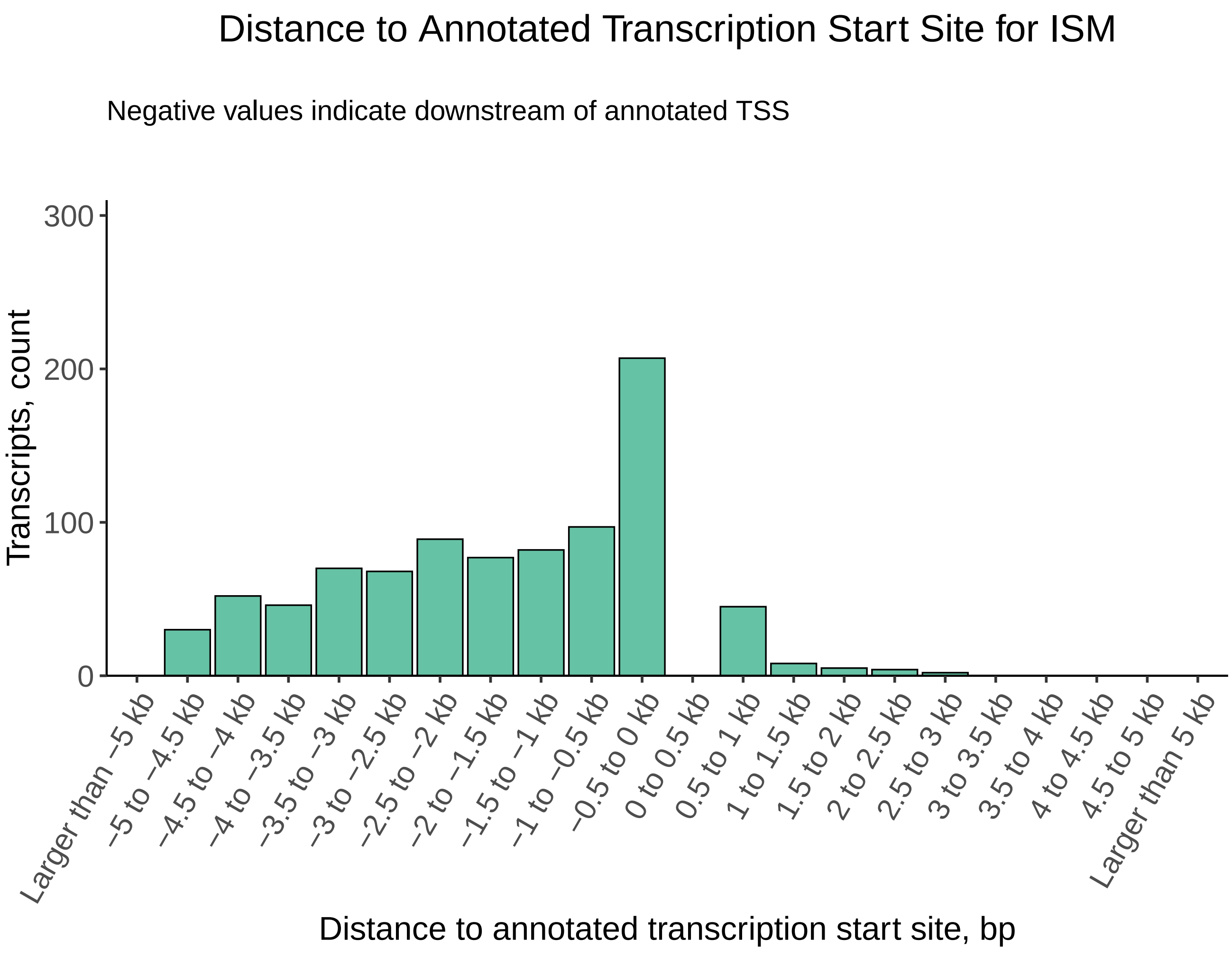

Distance to Annotated Transcription Start Site for ISM
Negative values indicate downstream of annotated TSS
Transcripts, count
300
200
100
0
Larger than −5 kb
−5 to −4.5 kb
−4.5 to −4 kb
−4 to −3.5 kb
−3.5 to −3 kb
−3 to −2.5 kb
−2.5 to −2 kb
−2 to −1.5 kb
−1.5 to −1 kb
−1 to −0.5 kb
−0.5 to 0 kb
0 to 0.5 kb
0.5 to 1 kb
1 to 1.5 kb
1.5 to 2 kb
2 to 2.5 kb
2.5 to 3 kb
3 to 3.5 kb
3.5 to 4 kb
4 to 4.5 kb
4.5 to 5 kb
Larger than 5 kb
Distance to annotated transcription start site, bp

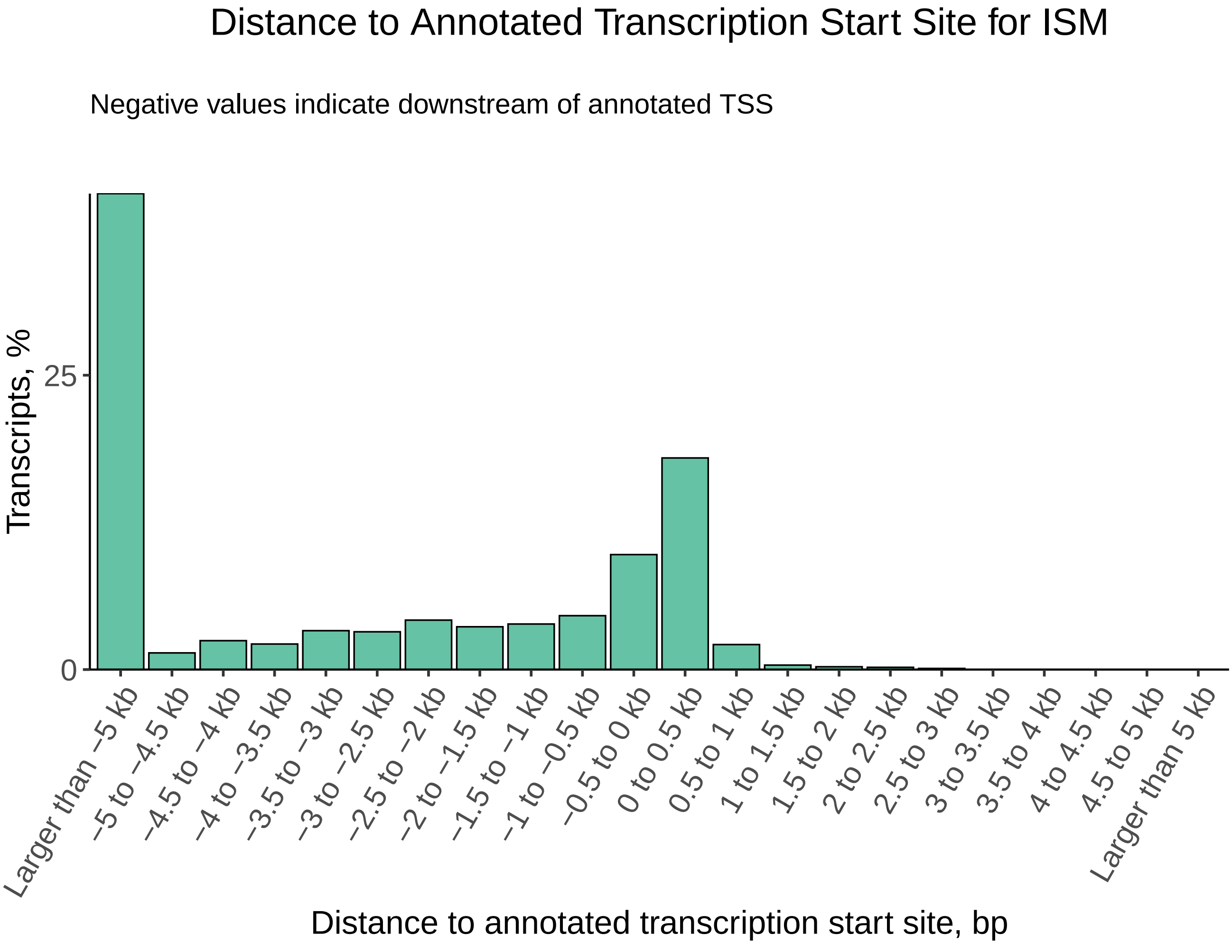
Distance to Annotated Transcription Start Site for ISM
Negative values indicate downstream of annotated TSS
Transcripts, %
25
0
Larger than −5 kb
−5 to −4.5 kb
−4.5 to −4 kb
−4 to −3.5 kb
−3.5 to −3 kb
−3 to −2.5 kb
−2.5 to −2 kb
−2 to −1.5 kb
−1.5 to −1 kb
−1 to −0.5 kb
−0.5 to 0 kb
0 to 0.5 kb
0.5 to 1 kb
1 to 1.5 kb
1.5 to 2 kb
2 to 2.5 kb
2.5 to 3 kb
3 to 3.5 kb
3.5 to 4 kb
4 to 4.5 kb
4.5 to 5 kb
Larger than 5 kb
Distance to annotated transcription start site, bp

Comparison With Annotated TSS and TTS

by Subcategories

PolyA Distance Analysis

Redundancy Analysis

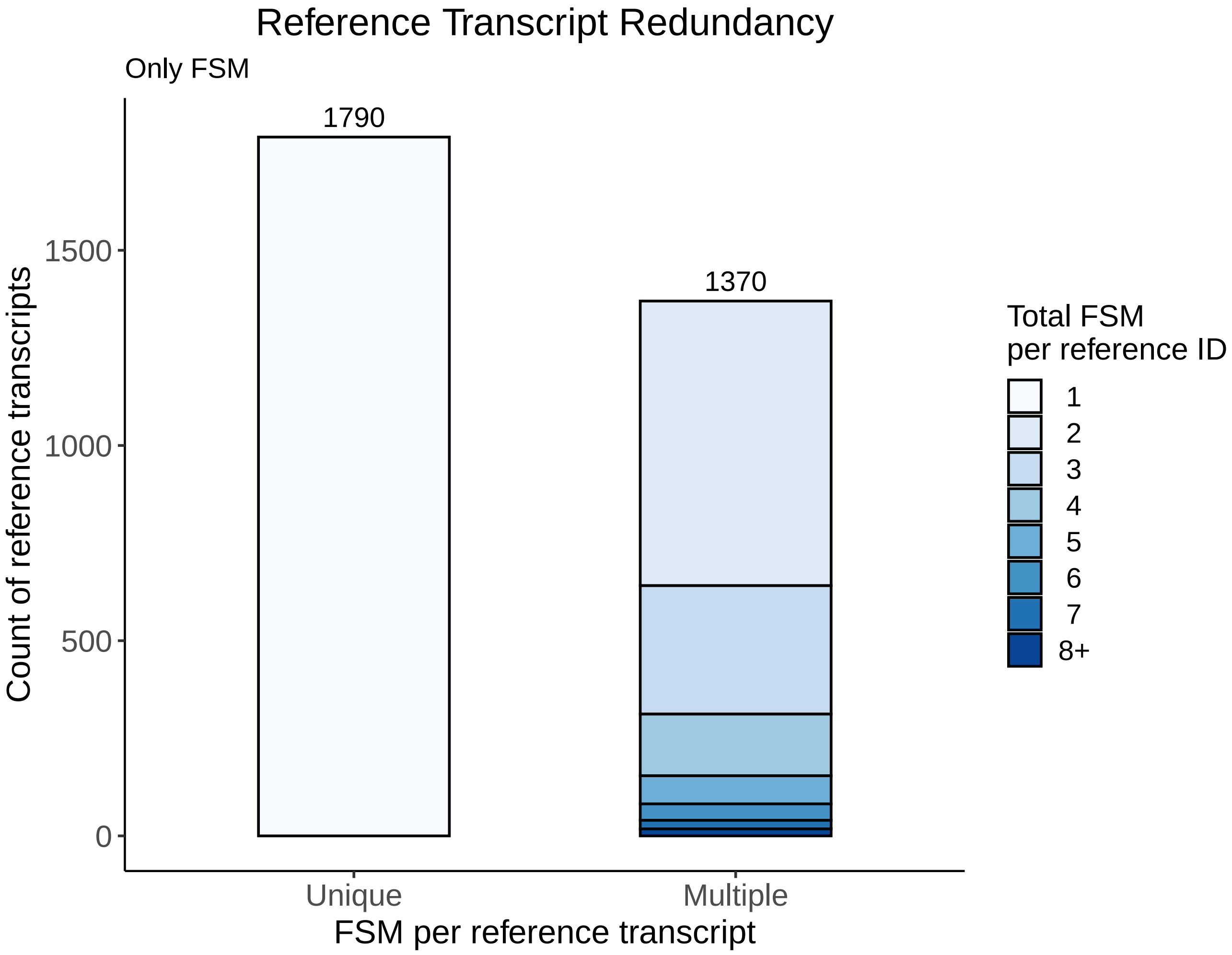
Reference Transcript Redundancy
Only FSM
1790
1370
Count of reference transcripts
1500
1000
500
0
Unique
Multiple
FSM per reference transcript
Total FSM
per reference ID
1
2
3
4
5
6
7
8+

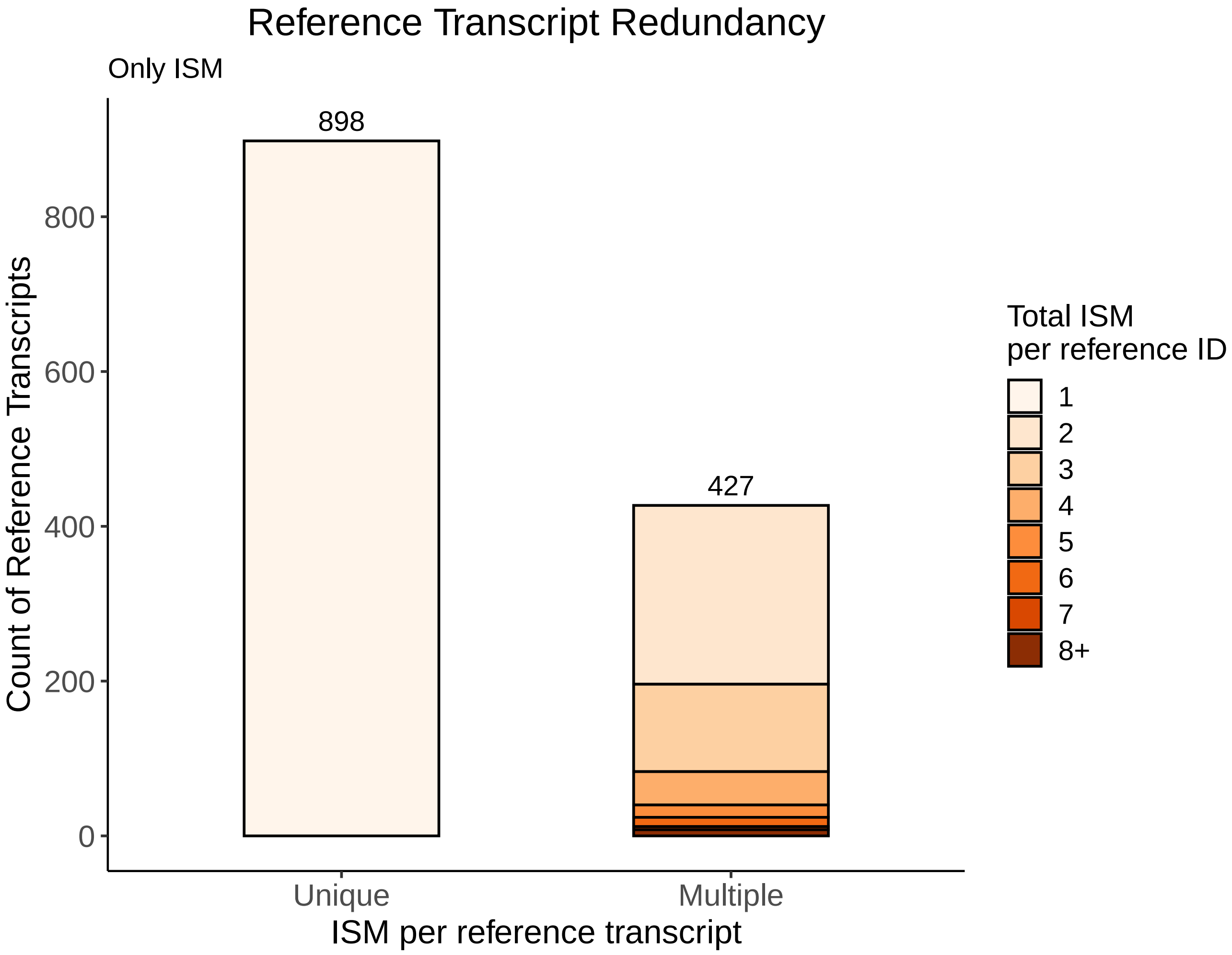
Reference Transcript Redundancy
Only ISM
898
427
800
600
400
200
0
Count of Reference Transcripts
Unique
Multiple
ISM per reference transcript
Total ISM
per reference ID
1
2
3
4
5
6
7
8+

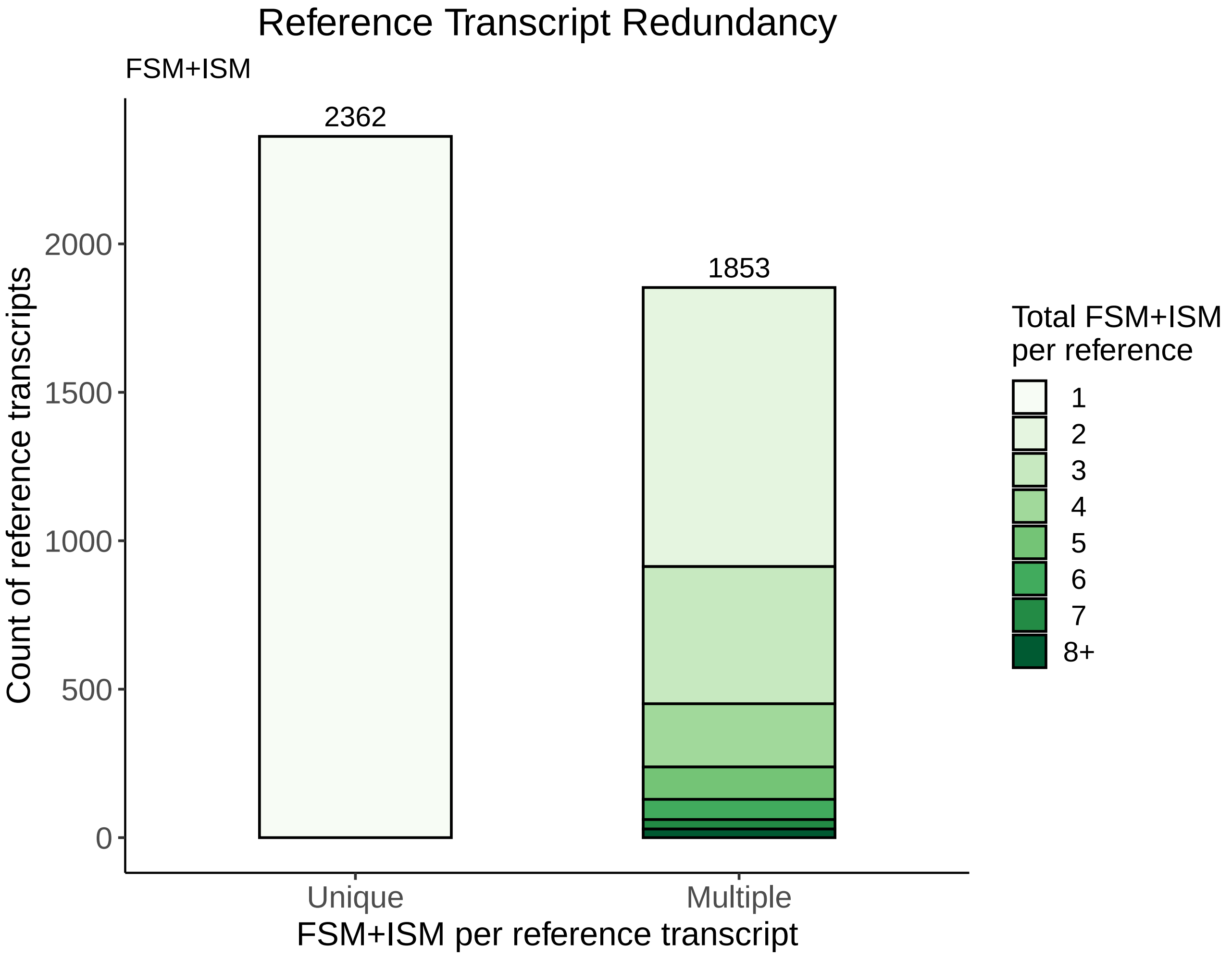
Reference Transcript Redundancy
FSM+ISM
2362
1853
Count of reference transcripts
0
500
1000
1500
2000
Unique
Multiple
FSM+ISM per reference transcript
Total FSM+ISM
per reference
1
2
3
4
5
6
7
8+

Intra−Priming Quality Check

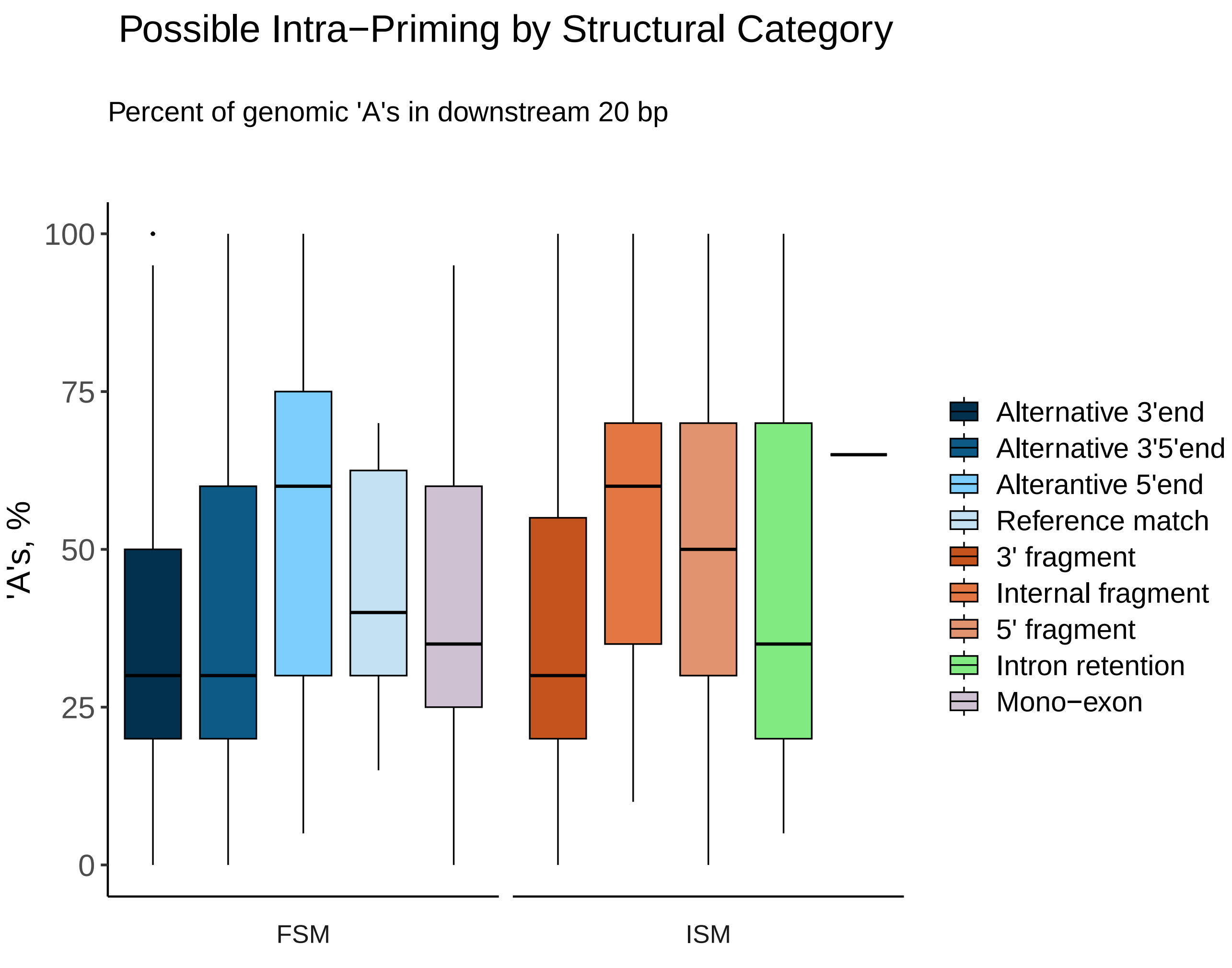
Possible Intra−Priming by Structural Category
Percent of genomic 'A's in downstream 20 bp
'A's, %
100
75
50
25
0
FSM
ISM
Alternative 3'end
Alternative 3'5'end
Alterantive 5'end
Reference match
3' fragment
Internal fragment
5' fragment
Intron retention
Mono−exon

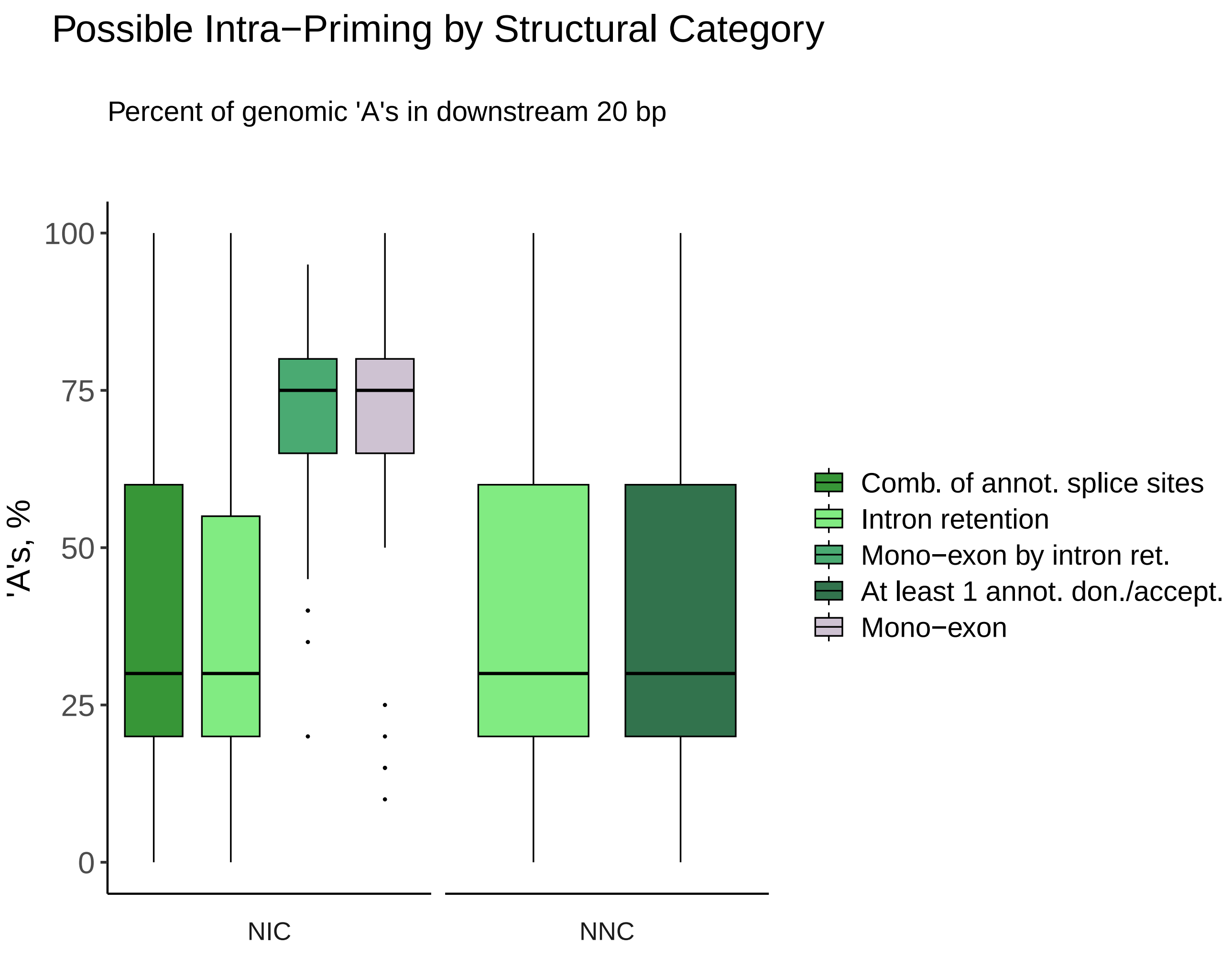

Possible Intra−Priming by Structural Category
Percent of genomic 'A's in downstream 20 bp
'A's, %
100
75
50
25
0
NIC
NNC
Comb. of annot. splice sites
Intron retention
Mono−exon by intron ret.
At least 1 annot. don./accept.
Mono−exon

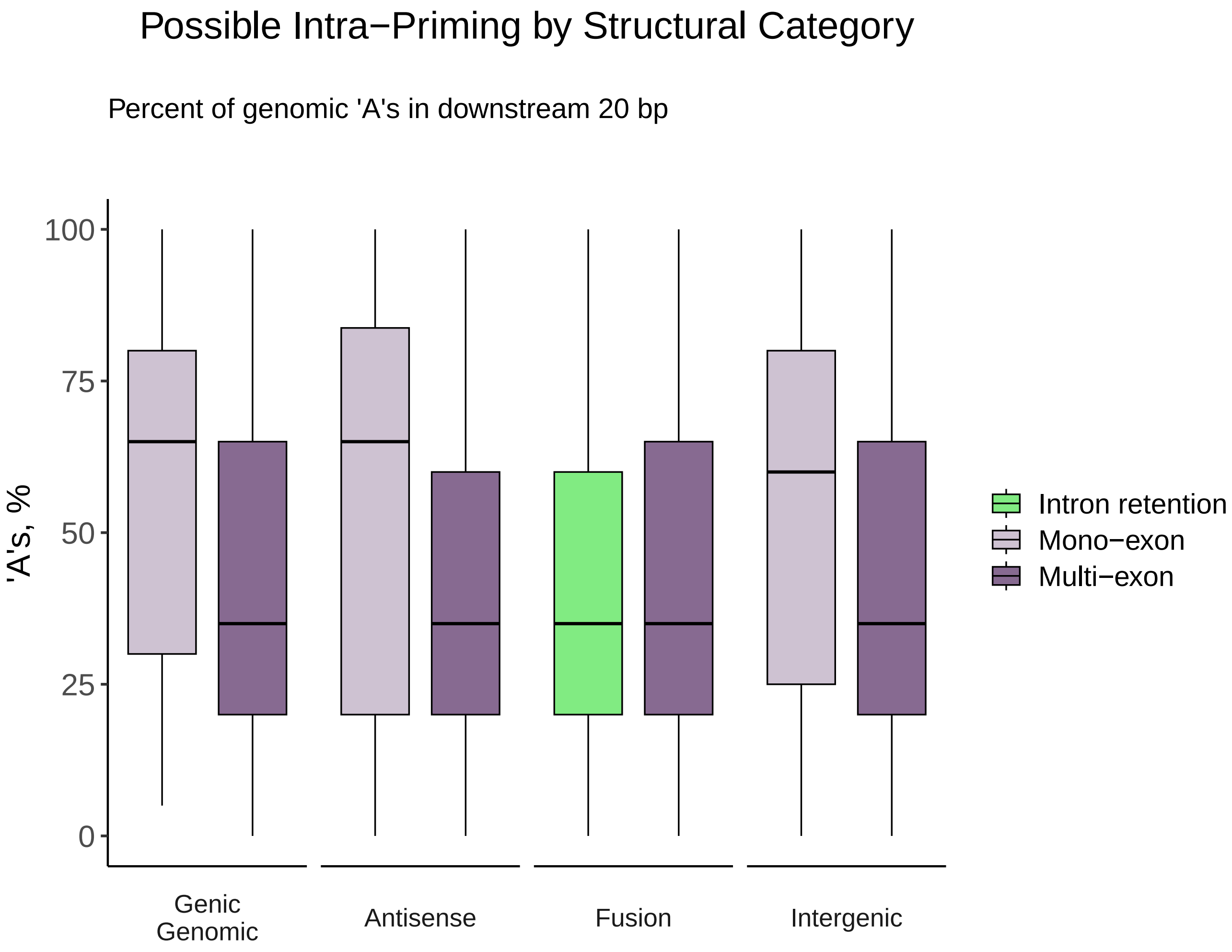

Possible Intra−Priming by Structural Category
Percent of genomic 'A's in downstream 20 bp
'A's, %
100
75
50
25
0
Genic
Genomic
Antisense
Fusion
Intergenic
Intron retention
Mono−exon
Multi−exon

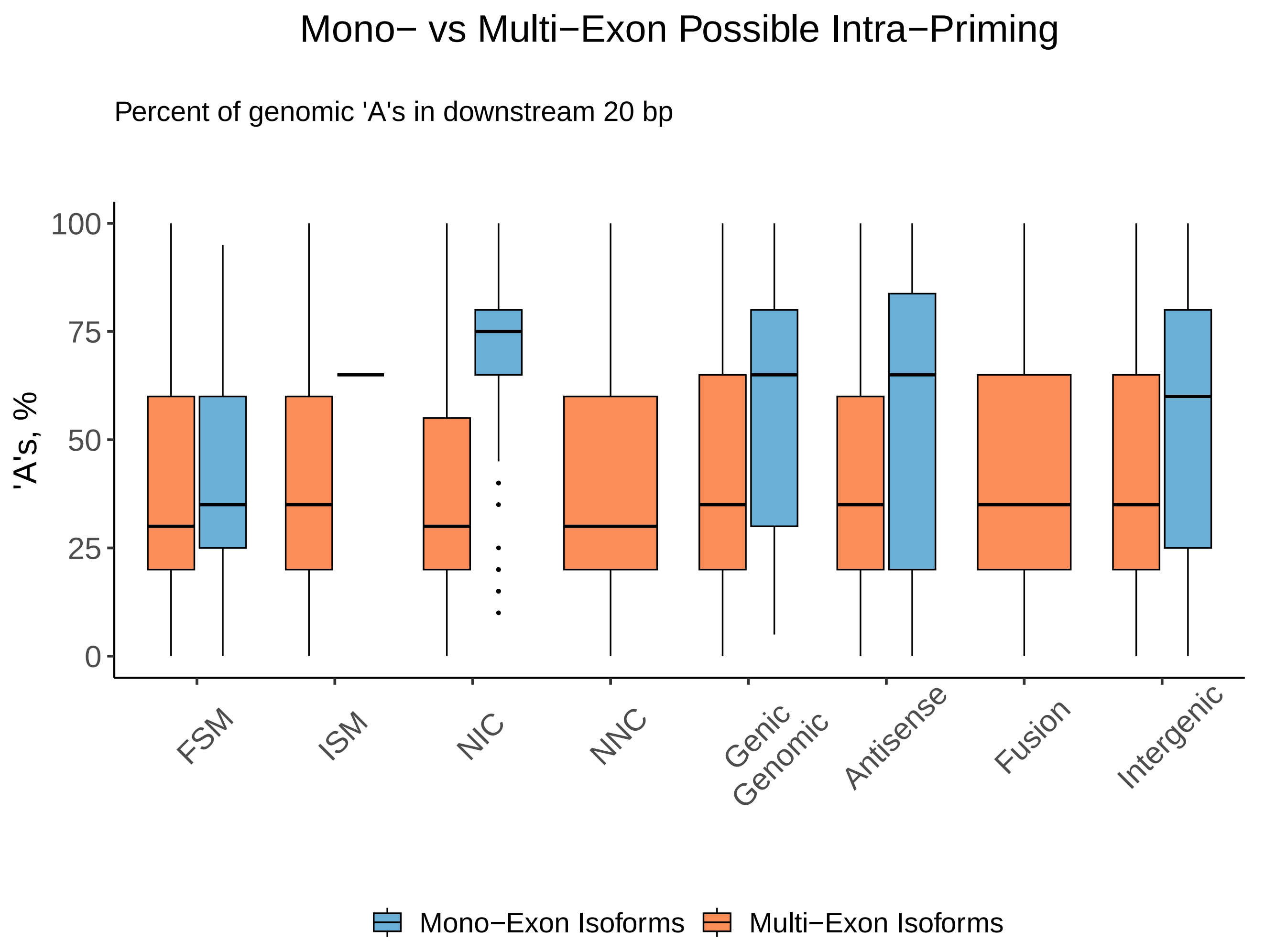
Mono− vs Multi−Exon Possible Intra−Priming
Percent of genomic 'A's in downstream 20 bp
'A's, %
100
75
50
25
0
FSM
ISM
NIC
NNC
Genic
Genomic
Antisense
Fusion
Intergenic
Mono−Exon Isoforms
Multi−Exon Isoforms

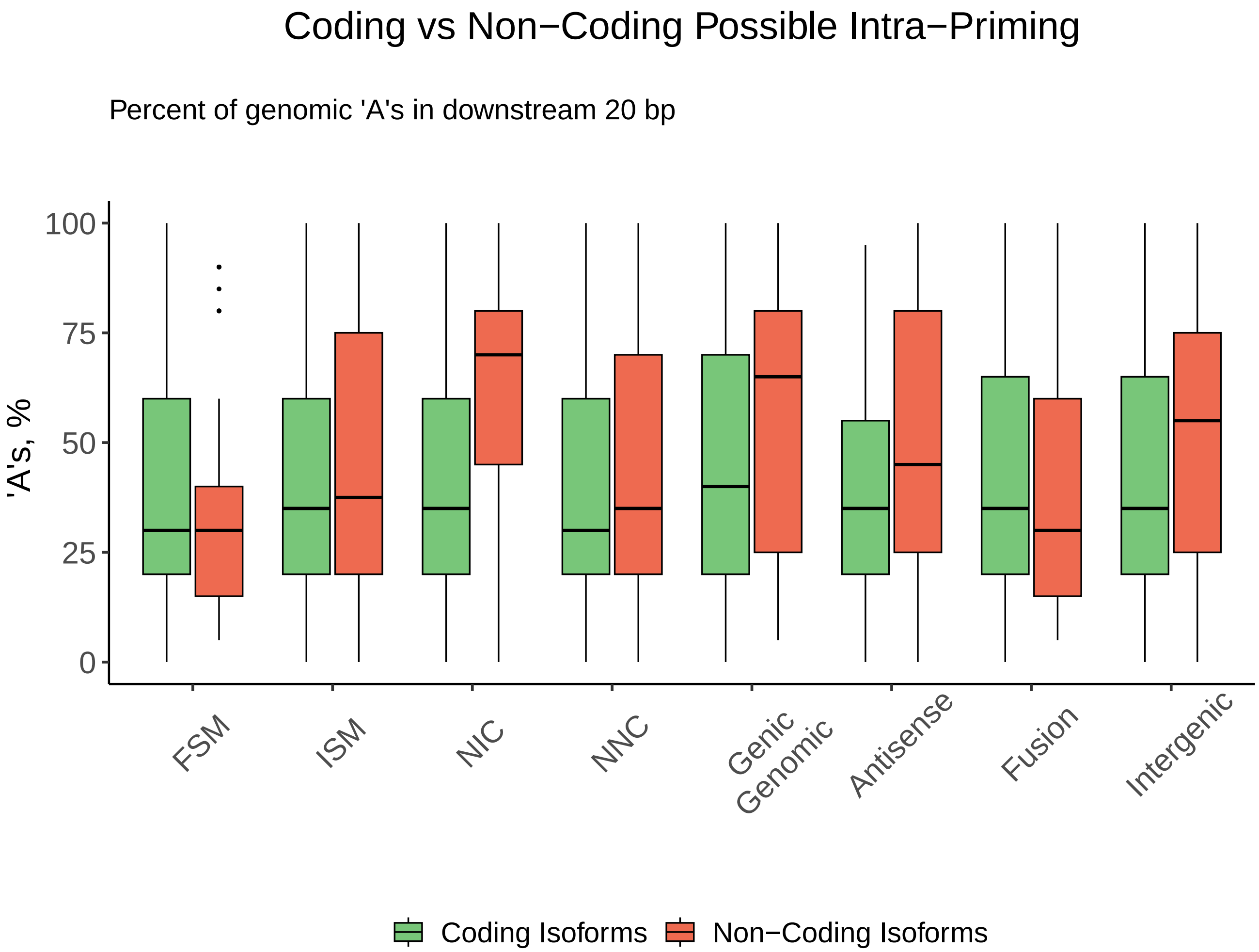
Coding vs Non−Coding Possible Intra−Priming
Percent of genomic 'A's in downstream 20 bp
'A's, %
100
75
50
25
0
FSM
ISM
NIC
NNC
Genic Genomic
Antisense
Fusion
Intergenic
Coding Isoforms
Non−Coding Isoforms

Features of Bad Quality

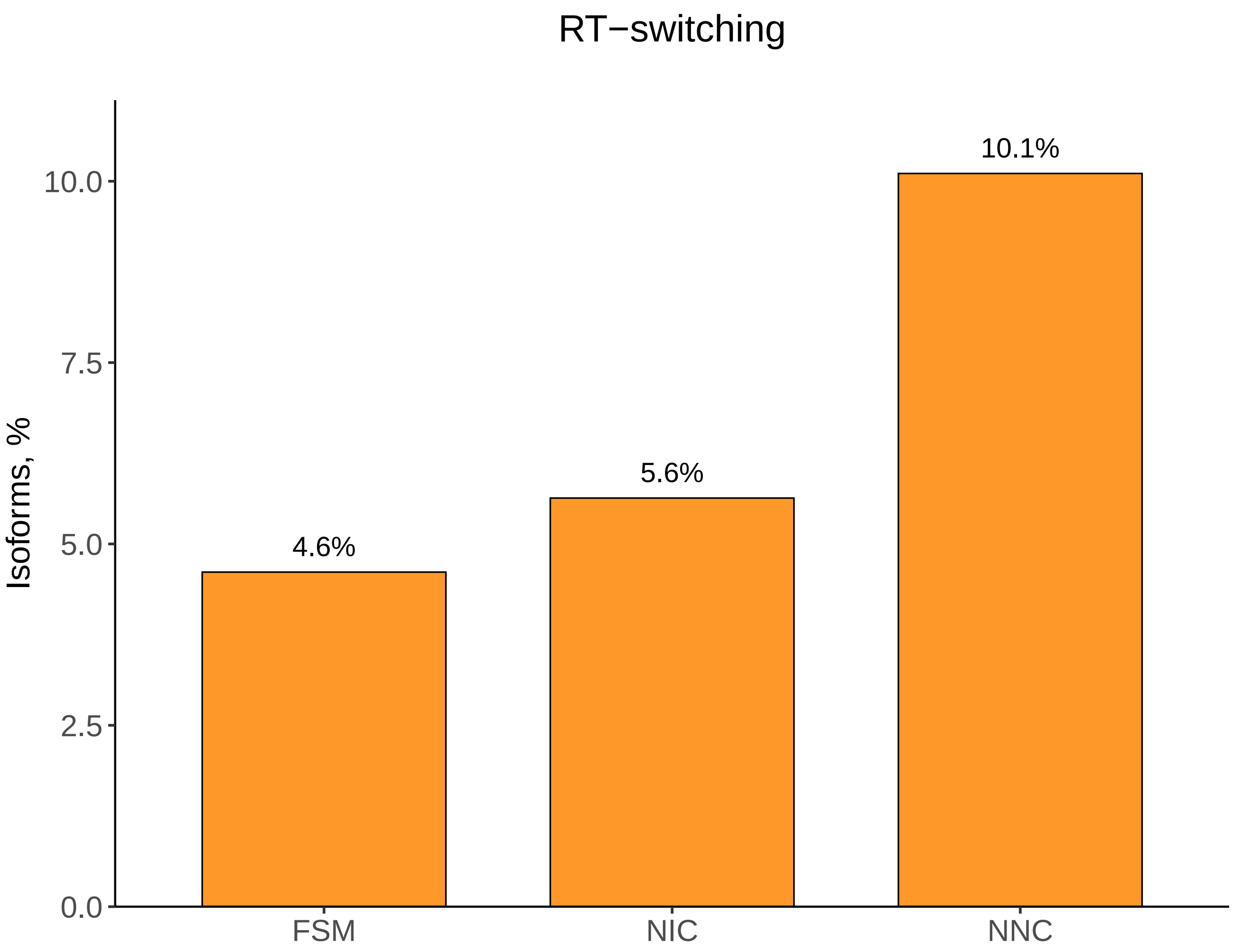
RT−switching
Isoforms, %
10.0
7.5
5.0
2.5
0.0
4.6%
5.6%
10.1%
FSM
NIC
NNC

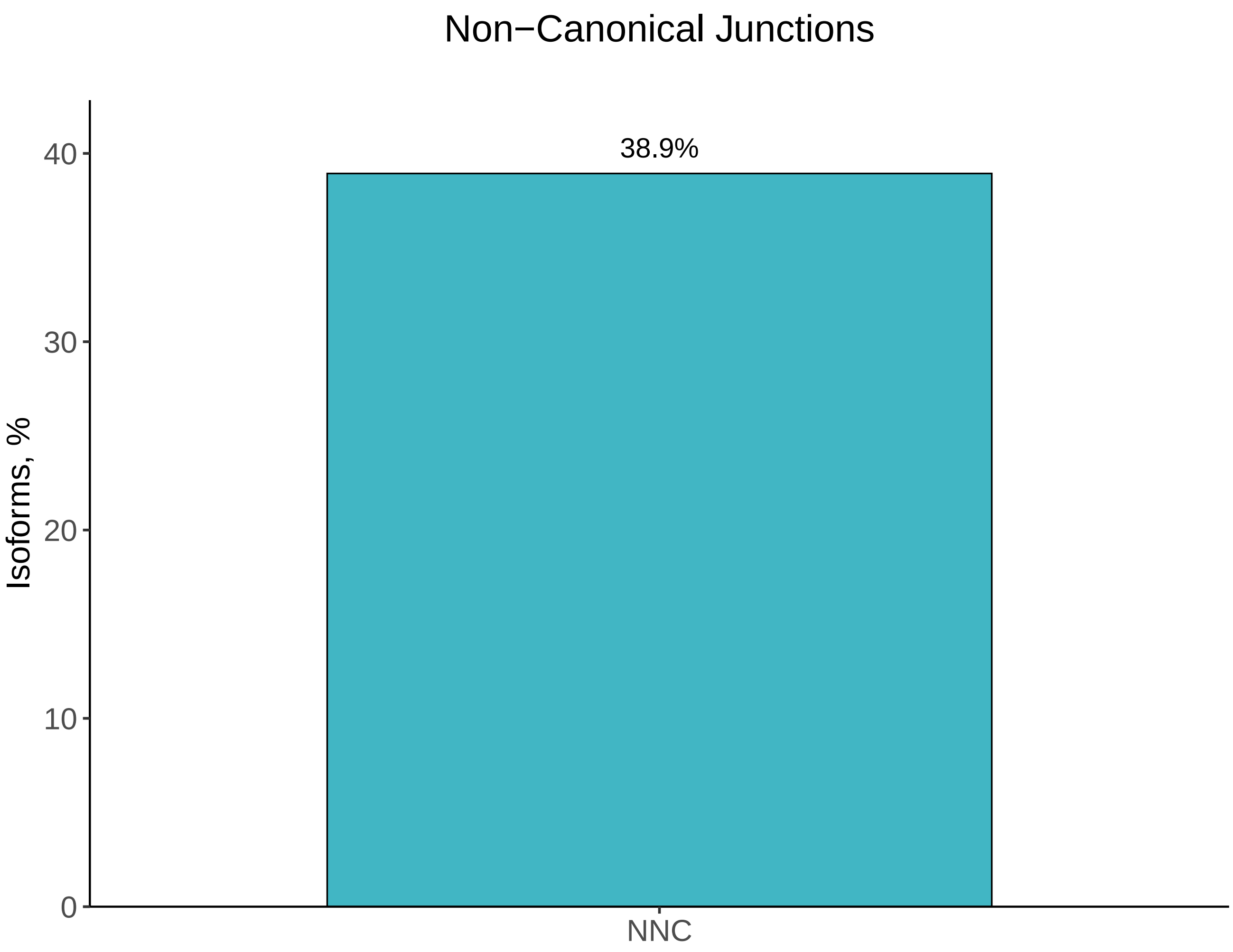
Non−Canonical Junctions
38.9%
Isoforms, %
0
10
20
30
40
NNC

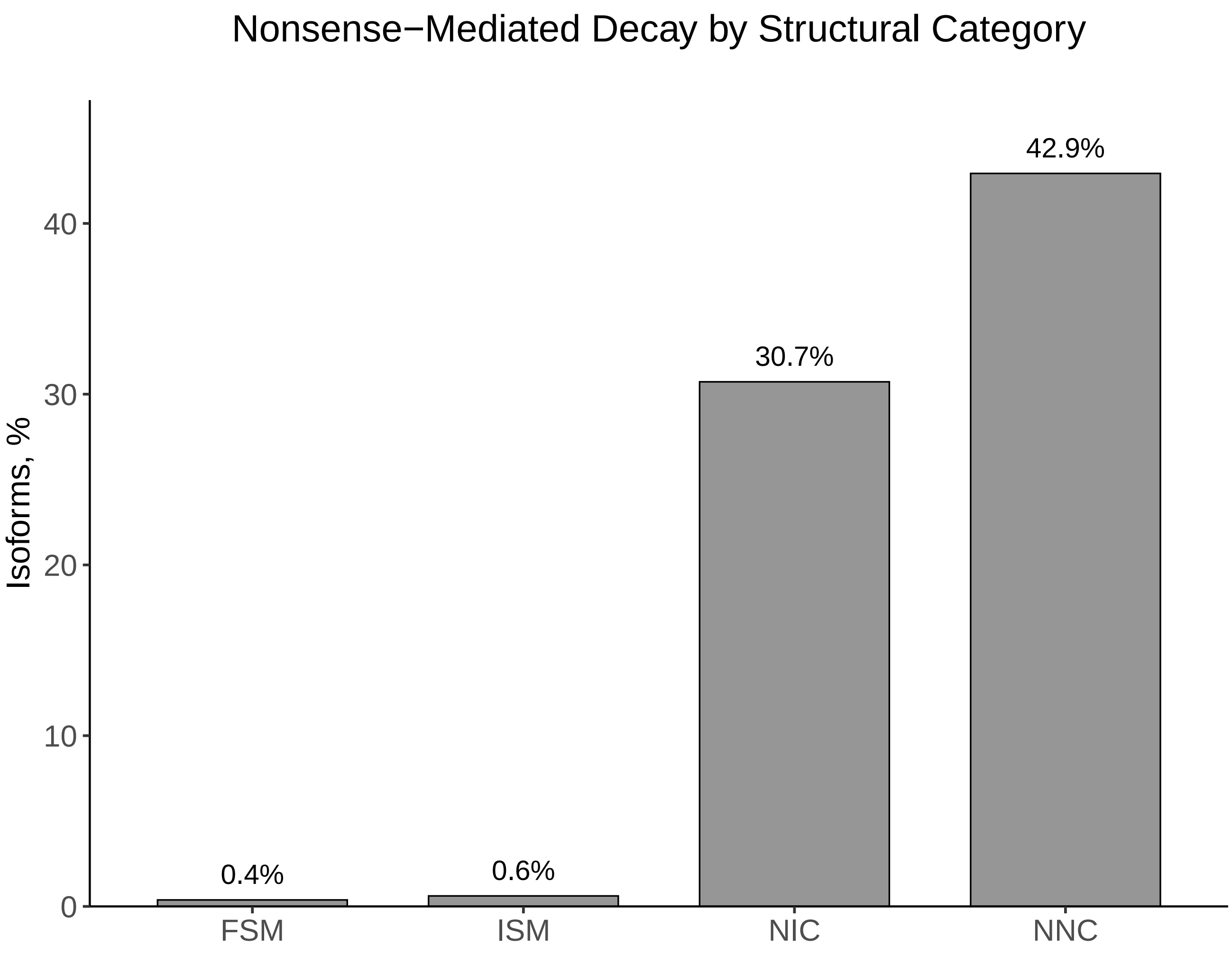
Nonsense−Mediated Decay by Structural Category
Isoforms, %
0
10
20
30
40
0.4%
0.6%
30.7%
42.9%
FSM
ISM
NIC
NNC

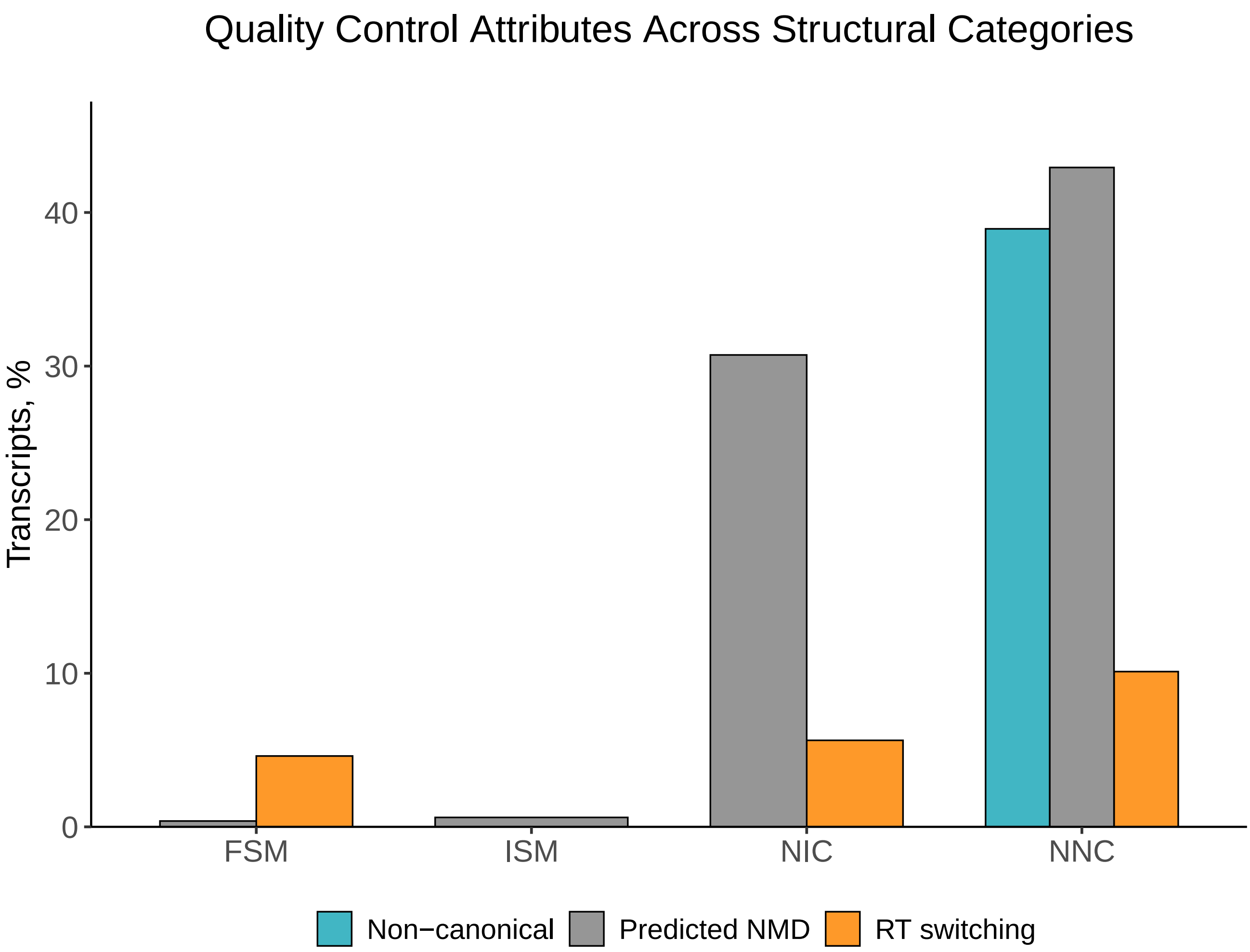
Quality Control Attributes Across Structural Categories
Transcripts, %
0
10
20
30
40
FSM
ISM
NIC
NNC
Non−canonical
Predicted NMD
RT switching

Features of Good Quality

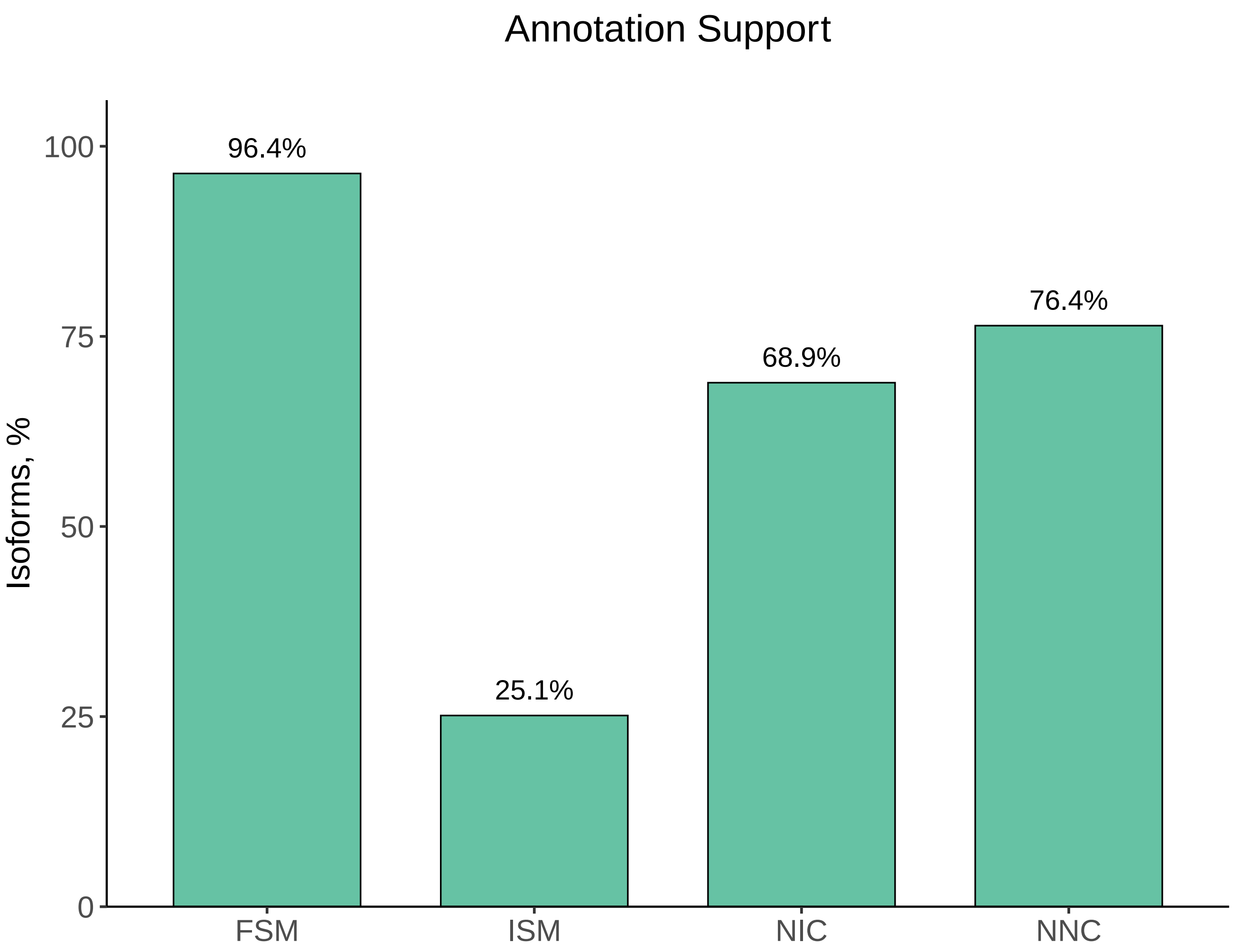
Annotation Support
Isoforms, %
100
75
50
25
0
96.4%
25.1%
68.9%
76.4%
FSM
ISM
NIC
NNC

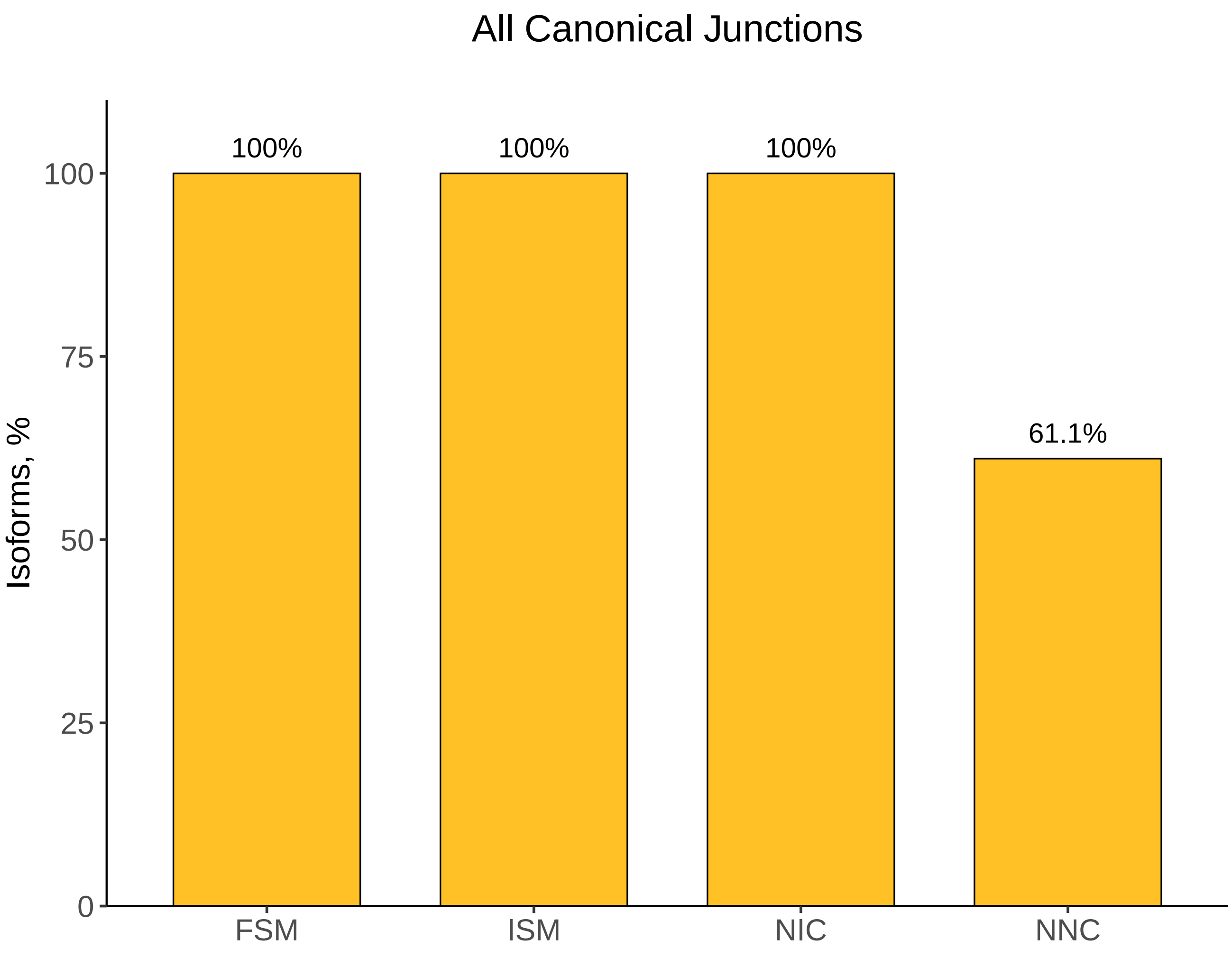
All Canonical Junctions
Isoforms, %
100%
100%
100%
61.1%
0
25
50
75
100
FSM
ISM
NIC
NNC

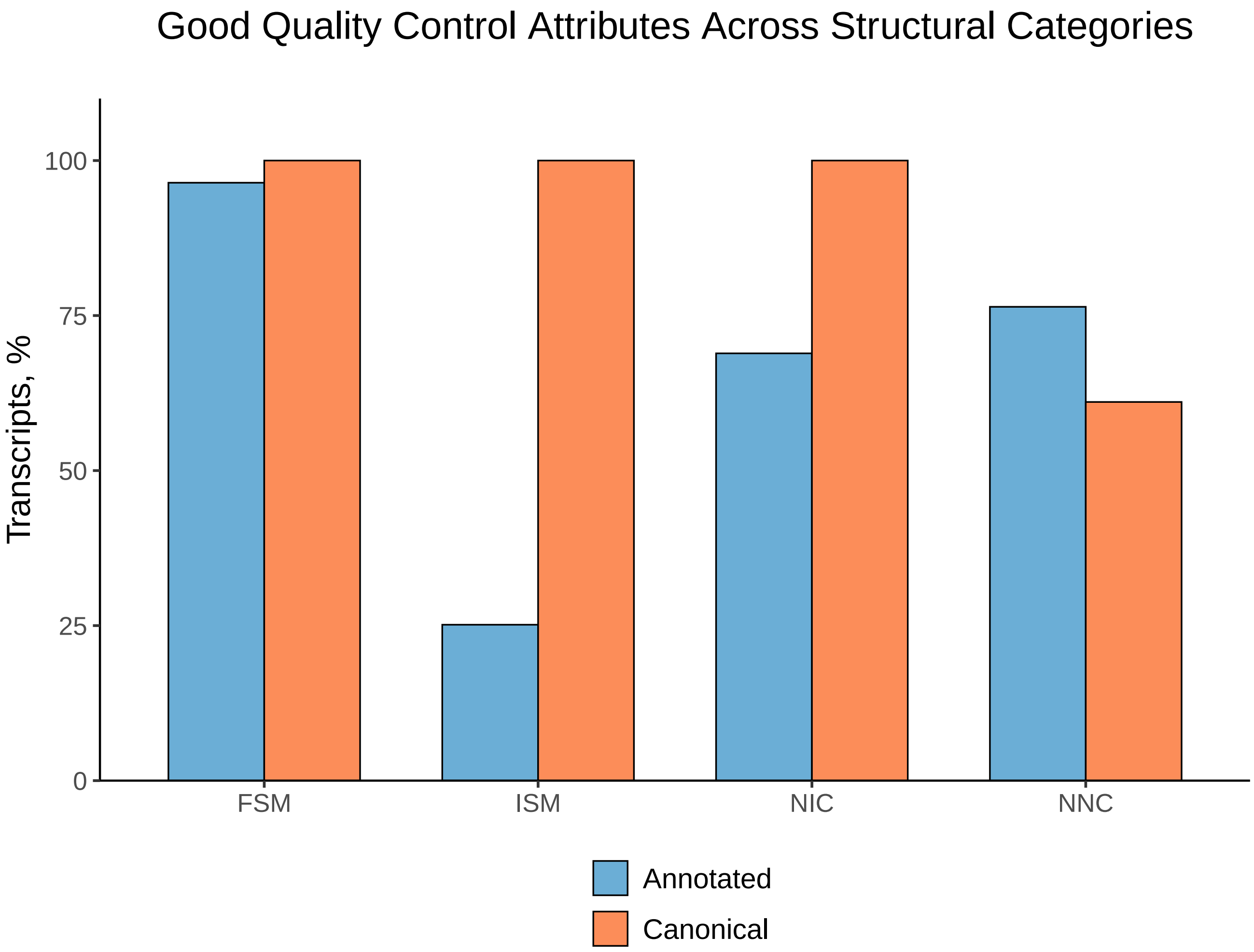
Good Quality Control Attributes Across Structural Categories
Transcripts, %
100
75
50
25
0
FSM
ISM
NIC
NNC
Annotated
Canonical
